# Novel Cu(ii) acidic deep eutectic solvent as an efficient and green multifunctional catalytic solvent system in base-free conditions to synthesize 1,4-disubstituted 1,2,3-triazoles†

**DOI:** 10.1039/d3ra06570g

**Published:** 2023-12-14

**Authors:** Nastaran Bagherzadeh, Mohammad Amiri, Ali Reza Sardarian

**Affiliations:** a Chemistry Department, College of Sciences, Shiraz University Shiraz Iran sardarian@shirazu.ac.ir +98 7136460788 +98 7136137107

## Abstract

A novel green Cu(ii)-acidic deep eutectic solvent (Cu(ii)-ADES) bearing copper salt, choline chloride, and gallic acid ([ChCl]_4_[2GA-Cu(ii)]) was synthesized and thoroughly specified by physicochemical approaches such as FT-IR, EDX, XRD, Mapping, ICP, and UV-Vis analyses and physicochemical properties. After the detection of authentic data, the central composite design (CCD) was utilized to accomplish the pertaining tests and develop the optimum condition, and, in the following, [ChCl]_4_[2GA-Cu(ii)] was applied as a green multifunctional catalytic solvent system in reducing agent-free and base-free condition for the three-component click reaction from sodium azide, alkyl, allyl, ester, and benzyl halide, and terminal alkyne made from amines and caprolactam as a cyclic amide to furnish a successful new library of 1,4-disubstituted 1,2,3-triazoles with a yield of up to 98%. The Cu(ii)-ADES is stable and can comfortably be recovered and reused without a considerable decline in its acting for seven cycles. This triazole synthesizing methodology is distinguished *via* its atom economy, operational facility, short reaction times, diverse substrate scope, and high performance for large-scale synthesis of the desired products including: –CN and –NO_2_ as efficient functional groups.

## Abstract 2

A novel Cu(ii)-acidic deep eutectic solvent was synthesized by the eco-friendly strategy and applied as multifunctional catalytic solvent system to furnish high yield of 1,2,3-triazoles in reducing agent- and base-free condition *via* CuAAC reaction.

## Graphical abstract

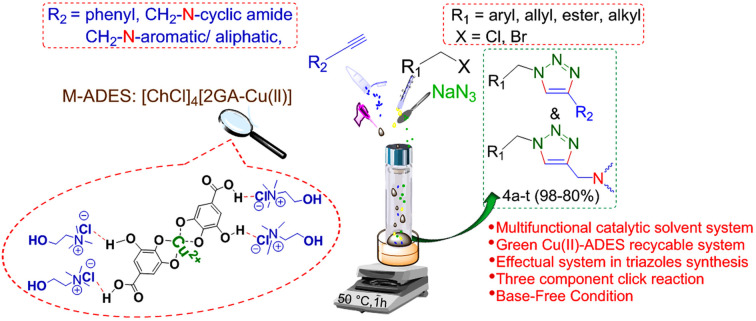

## 1. Introduction

**Fig. 1:**
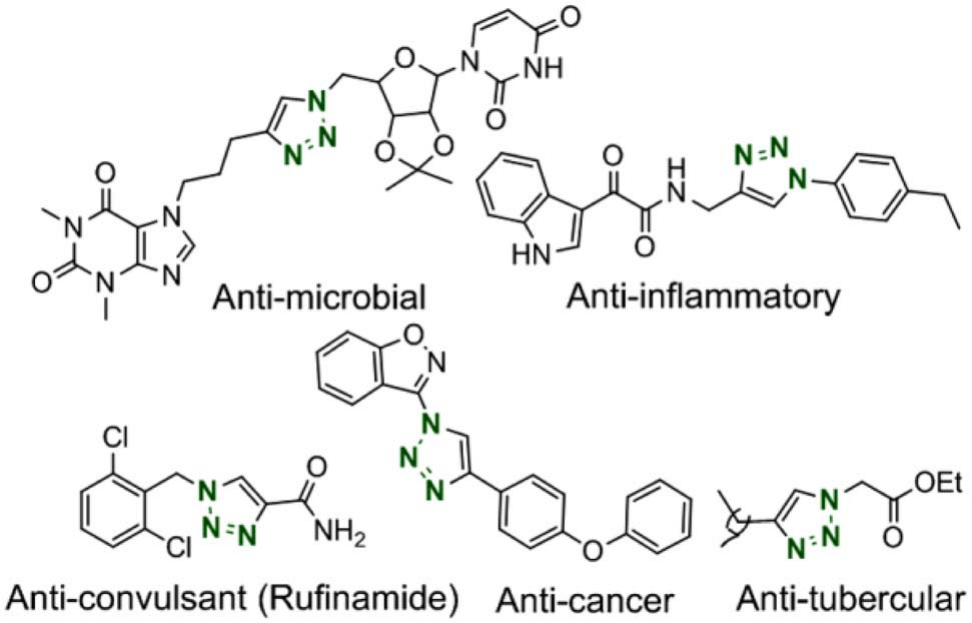
Some representative scaffolds of bioactive 1,4-disubstituted 1,2,3-triazoles.

Heterocyclic 1,2,3-triazoles are a privileged category of nitrogenous heterocyclic compounds attracting a high extent of consideration in industrial and academic areas. These critical structures have found an extensive range of applications in chemical biology,^1^ organic synthesis,^2^ supramolecular chemistry,^3^ materials science,^4^ and pharmaceuticals chiefly offered for their anti-tumor, anti-microbial, anti-viral, anti-cancer, anti-tubercular, anti-inflammatory.^5^ Some of the substantial scaffolds are exhibited in Fig. 1. Furthermore, these compounds have beneficial properties like hydrogen bonding ability, excellent chemical stability, intense dipole moment and π-stacking interaction, and aromatic character.^6^ Accordingly, the establishment of mild and convenient methodologies have been encouraged for synthesizing these compounds.

The most popular method was reported by Sharpless's laboratories, as the factual pioneers in this theme through the Cu(i)-catalyzed Huisgen cycloaddition, which is well known as Cu-catalyzed azide–alkyne cycloaddition (CuAAC).^7^

**Scheme 1:**
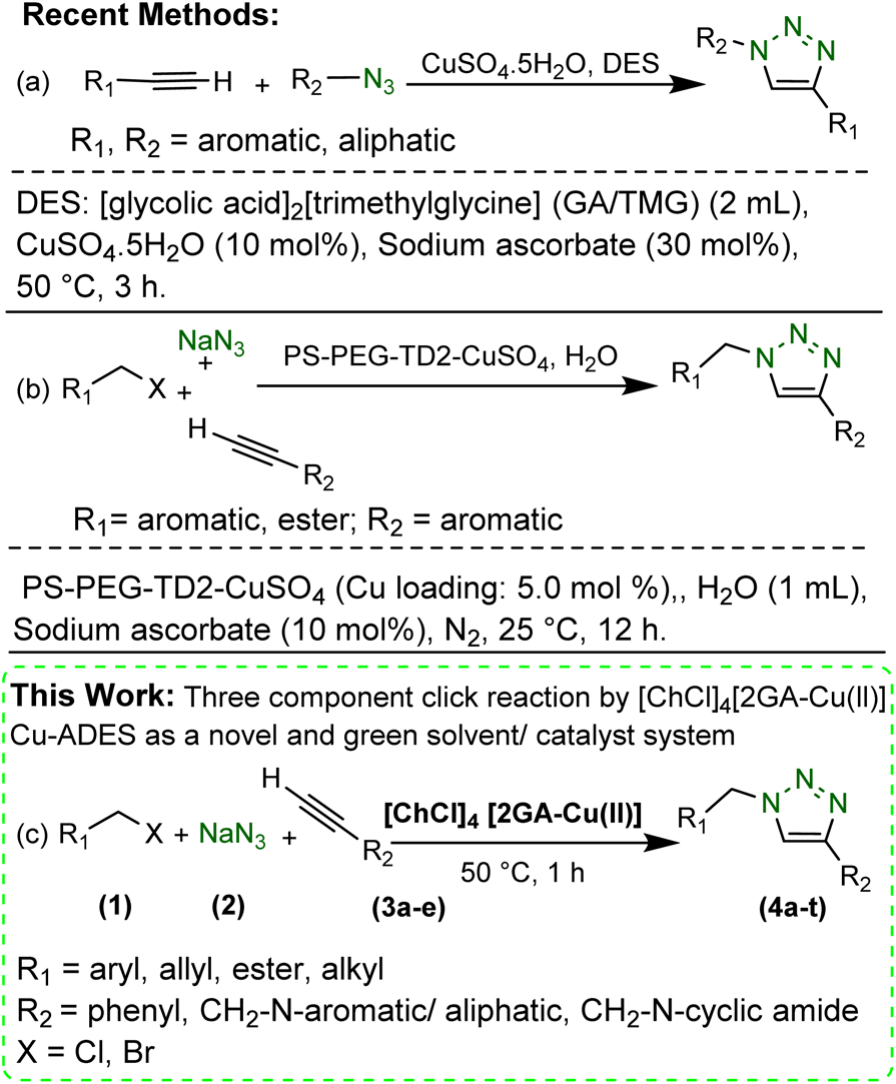
Preparation of 1,4-disubstituted 1,2,3-triazoles.

There are many reports in which the regioselective production of 1,4-disubstituted 1,2,3-triazoles has been carried out using the CuAAC strategy.^8–11^ There are several negative aspects in most of these reported methodologies, such as (a) utilization of non-reusable catalysts, (b) application of hazardous volatile organic solvents that are significantly problematic for the environment, (c) using prefabricated organic azides that are unstable, explosive, commercially unavailable, and (d) demanding various additives when Cu(ii) catalysts are utilized (*e.g.* large amount of reducing agent).^10,11^ Thus, many efforts have been taken to develop new CuAAC sustainable chemical protocols for the production of 1,4-disubstituted 1,2,3-triazoles using reusable catalysts and green solvents.^11*a*,*b*,12,13,14^ Among these methods, it can be pointed, for instance, to Giofrè *et al.*^12^ (Scheme 1a) and Uozumi *et al.*^13^ (Scheme 1b). Nonetheless, these methodologies have their merits as using deep eutectic solvents (DESs) or water as green solvent and reusable heterogeneous Cu catalysts however include one or several weak points as the application of (a) large amounts of sodium ascorbate for *in situ* reduction of Cu(ii) to Cu(i), and (b) expensive and multistep synthesized Cu(ii) heterogeneous catalyst. Consequently, there is still demand for developing new economical, benign, and simple handling methodologies for the preparation of the 1,2,3-triazoles.

Fascinating features of DESs as a specific type of ionic liquids (ILs) with their distinct superiorities *versus* ILs,^15^ such as simplicity in preparation of ideal atom economy synthesis, non-toxicity, non-volatility, non-flammability, easy recyclability, thermal stability, and most importantly, susceptibility to dissolve polar and nonpolar reagents,^16,17^ motivate us to design a deep eutectic solvent as a novel dual solvent/catalyst for the CuAAC reaction.

Thus, we report the manufacture and characterization of a novel copper acidic deep eutectic solvent (Cu-ADES), bearing choline chloride (ChCl), gallic acid (GA), and CuCl_2_, which are non-toxic, inexpensive, and available chemicals. Then this DES ([ChCl]_4_[2GA-Cu(ii)]) is applied as an efficient, reusable, and sustainable dual solvent and catalyst system in the base-free three-component click reaction NaN_3_, benzyl, alkyl halides, and terminal alkynes constructed of amine and cyclic amide resources as abundant azide precursors for the regioselective synthesis of novel 1,4-disubstituted 1,2,3-triazole frameworks, Scheme 1c.

## 2. Results and discussion

**Scheme 2:**
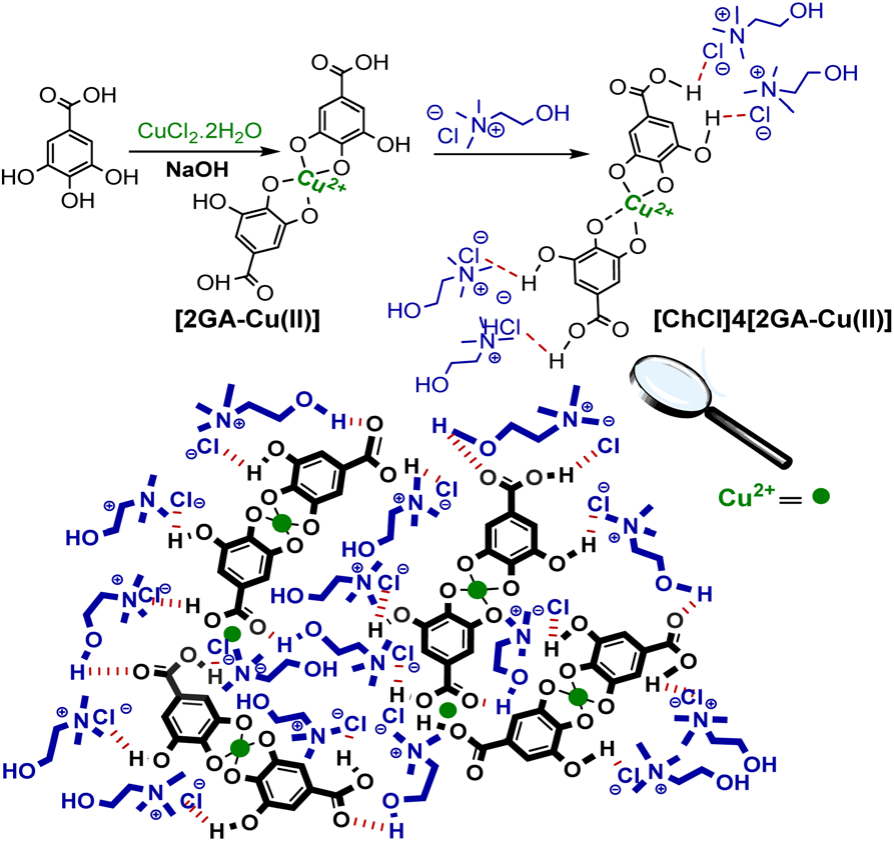
The suggested illustration of molecular structure of [2GA-Cu(ii)] complex and also possible interactions between [2GA-Cu(ii)] complex and choline chloride (ChCl).

In this work, the [2GA-Cu(ii)] complex was formed by mixing GA with CuCl_2_·2H_2_O with a molar ratio of 2 : 1 in an aqueous solution at room temperature in pH 7.5.^18,19^ Then the desired novel DES was formed as a dark brown liquid system using the previous procedure reported.^20^ A possible illustration of coordination between copper ion (Cu^2+^) and gallic acid (GA), in order to prepare Cu complex and interactions between [2GA-Cu(ii)] complex and choline chloride (ChCl) in the structure of the desired Cu(ii)-AEDS exhibited in Scheme 2.

### 2.1 Characterization of the [2GA-Cu(ii)] complex and [ChCl]_4_[2GA-Cu(ii)] as Cu(ii)-AEDS

**Fig. 2:**
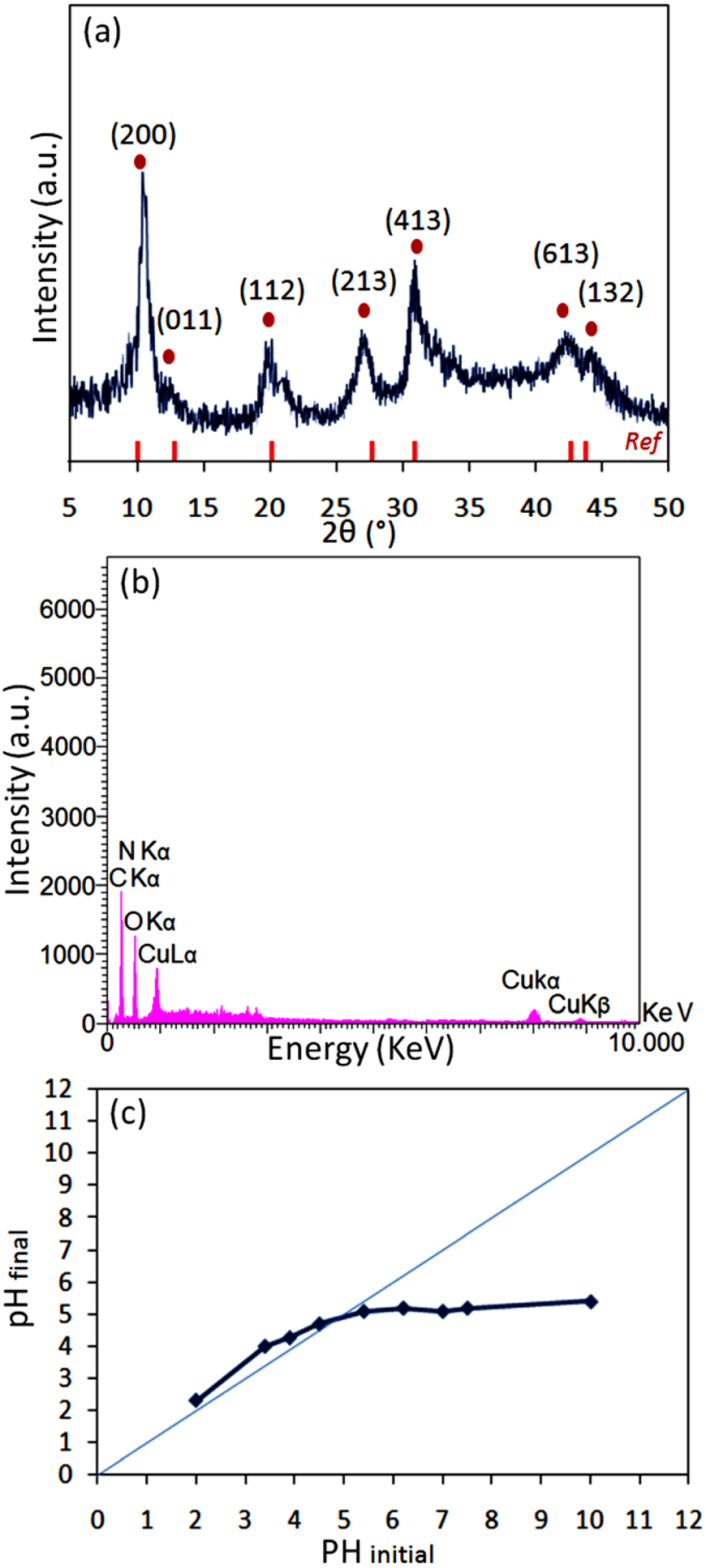
(a) Powder X-ray diffraction of [2GA-Cu(ii)]; (b) EDX analysis of [2GA-Cu(ii)]; (c) pH_pzc_ determination graph of [2GA-Cu(ii)].

Several physicochemical measurements were accomplished to illustrate the specifications of the [2GA-Cu(ii)] complex and [ChCl]_4_[2GA-Cu(ii)] as Cu(ii)-ADES. The prepared [2GA-Cu(ii)] and Cu(ii)-ADES were identified by Energy-dispersive X-ray (EDX), Mapping, XRD pattern, Fourier transform infrared (FTIR), ICP (Inductively coupled plasma), and Ultraviolet-Visible spectroscopy (UV-Vis) analyses. As shown in Fig. 2a, the XRD spectrum of [2GA-Cu(ii)] showed a close resemblance to the diffraction spectrum reported in the literature for this complex,^18,19^ specifically at 2*θ* = 10.1°, 13.2°, 20.1°, 28.0°, 31.1°, and 42.8°, and is an indication for successful production of the related copper complex.

EDX, as a helpful technique for the determination of constituent atomic components of a compound, was applied on [2GA-Cu(ii)]. The EDX spectrum revealed the presence of C, N, O, and Cu elements and also the absence of Cl element in the structure of [2GA-Cu(ii)] (Fig. 2b) and therefore confirms the synthesis of this metal-phenolic network.

The negative surface charge of [2GA-Cu(ii)] was proved *via* the pH_pzc_ value (Fig. 2c). This analysis was performed with the method recorded by Angkawijaya *et al.*^21^ in earlier reports. A series of 5 mL NaCl solutions (0.1 M) in covered vials were provided at an adjusted pH of 2 to 10. Afterward, in each vial, 15 mg of the present DES was added and allowed to be mixed in a shaking incubator with 200 rpm at 30 °C for 48 h. After passing the desired time, the final pH was measured for each sample with a pH meter. Then, the related pH_final_ was plotted against the pH_initial_ for each case, and the intersection of the pH_initial_ = pH_final_ linear graph with the experimental curve was distinguished as pH_pzc_, which is equal to 4.61. The dissociation of several proton (H^+^) ions from the functional groups –COOH and –OH cause the surface charge [2GA-Cu(ii)] to be negative, which is given the fact that the pH of a solution is higher than pH_pzc_. But while a series of [2GA-Cu(ii)] particles at pH ≥ 6 have a negative surface charge, may some of their functional groups stay protonated. Since the type of interaction between the DES components has not been clearly defined in the literature so far, it is assumed that these protonated groups of GA take part in multiple possible interactions consisting of dipole–dipole H-bonding, electrostatic H-bonding, electrostatic interaction between charged components,^20*b*^ also H-bonding of the carbonyl group of GA and the hydroxyl group of choline chloride when it interacts with choline chloride to form a eutectic mixture.

**Fig. 3:**
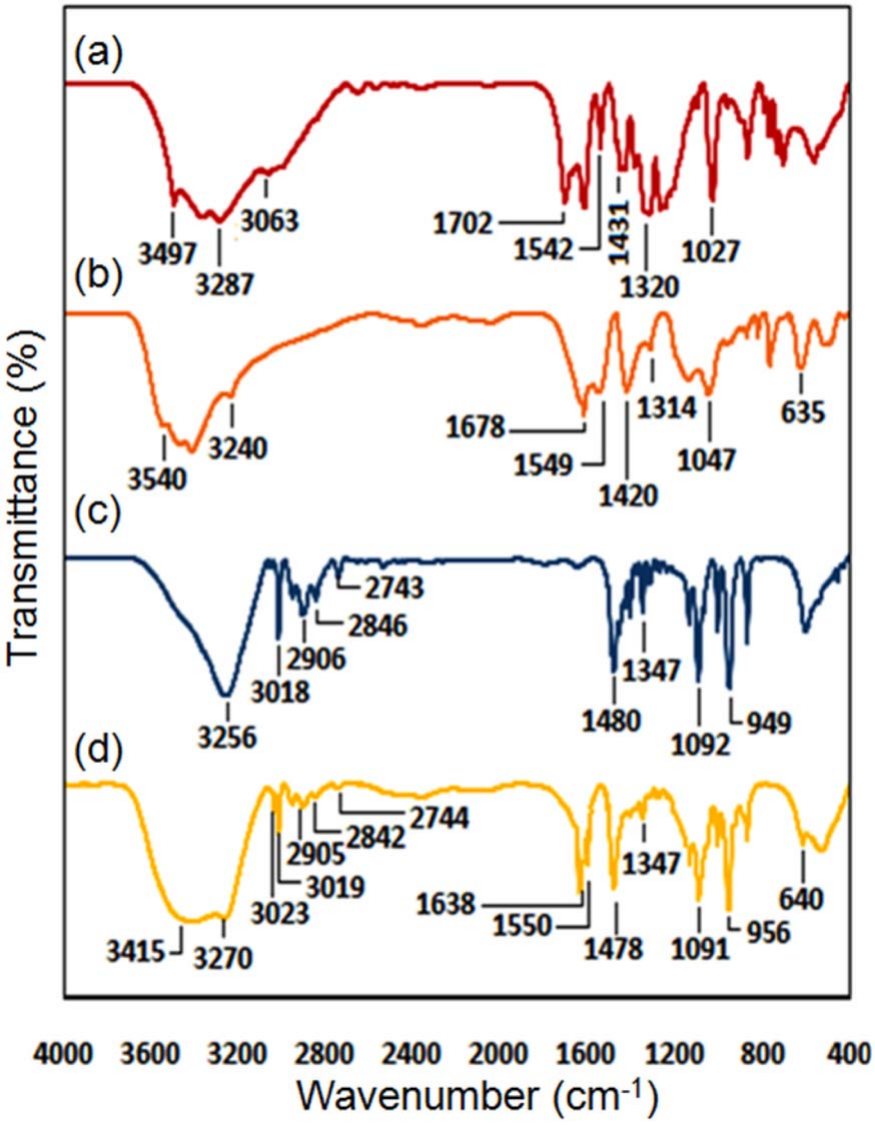
FT-IR spectra of (a) GA, (b) 2GA-Cu(ii), (c) ChCl, and (d) [ChCl]_4_[2GA-Cu(ii)].

The following investigation was done by FT-IR analysis for the characterization of functional groups in [2GA-Cu(ii)], and [ChCl]_4_[2GA-Cu(ii)] (Cu(ii)-ADES) and the FT-IR spectra of the parent components gallic acid (GA) and choline chloride (ChCl) were also given for comparison, which can be observed in the Fig. 3a–d and Table 1. As presented in (Fig. 3a, b, and d), the characteristic peaks of gallic acid can be observed in the FT-IR spectra of [2GA-Cu(ii)] and [ChCl]_4_[2GA-Cu(ii)], especially the acidic index peaks (2500–3600 cm^−1^), aromatic stretching of C=C (1540–1550 cm^−1^), C=O stretching (1635–1700 cm^−1^), bending vibrations of C–O (1420–1480 cm^−1^), and C–O bonds stretching peaks in the region of 1020–1090 cm^−1^. Further, in the [2GA-Cu(ii)] chromatogram, several band shifts can be perceived as a shift was observed from 1431 cm^−1^ in GA to 1420 cm^−1^ in [2GA-Cu(ii)] of the C–O vibration stretching band. The peak shifts related to phenol-C–O vibration in the GA spectrum at 1027 and 1250 cm^−1^, respectively, to 1047 and 1140 cm^−1^ in the [2GA-Cu(ii)] spectrum have been observed. The band shifts for these groups indicate a change in the bond length, which can be attributed to the coordination between Cu and GA. Moreover, the vibration of the Cu–O group at 635 cm^−1^ appears in the [2GA-Cu(ii)] spectrum, confirming the participation of the Cu–O group in the formation of [2GA-Cu(ii)] and is explicitly observed in the M-ADES spectrum (Fig. 3d) that elucidates the stability of Cu–O band formation in all synthetic steps.^21^ In continue, the incorporation of ChCl to the [2GA-Cu(ii)] and formation of eutectic brown liquid of Cu(ii)-ADES was confirmed by the shift of the (OH)–hydroxyl and (OH)–carboxyl stretching bands to lower wavenumbers in the Cu(ii)-ADES compared to those in the gallic acid due to formation of hydrogen bonding of these functional groups and several possible interactions, in addition to shifting in the (OH)–alcohol band (at ChCl) and C=O stretching (at GA) from 3256 to 3270, and 1702 to 1638 in Cu(ii)-ADES, respectively (Fig. 3d and Table 1). As well as peak shift at 949 cm^−1^ in the ChCl spectrum to 956 cm^−1^ [ChCl]_4_[2GA-Cu(ii)] spectrum is related to the C–N stretching in the ammonium structure. Also, the presence of bands at the round of 2740–3020 cm^−1^ specified the attendance of (–CH_2_–), (–CH_3_) symmetric and asymmetric stretching vibrations in the choline chloride and Cu(ii)-ADES^22^ (Fig. 3c and d). Eventually, the comparison study of FT-IR spectra of choline chloride, [2GA-Cu(ii)], and [ChCl]_4_[2GA-Cu(ii)] demonstrate that Cu(ii)-ADES formation does not lead to the establishment of any new functional groups in the final mixture.

**Table 1:** Selected FT-IR peaks of the GA, [2GA-Cu(ii)], ChCl, and [ChCl]_4_[2GA-Cu(ii)]

GA^a^	FT-IR spectra (cm^−1^)			Types of bond vibration of functional groups
	GA-Cu(ii)	ChCl^b^	[ChCl]_4_[2GA-Cu(ii)]	
1027, 1250	1047, 1140	1092	1091	*ῡ* (C–O)
1320	1314	1347	1347	*ῡ* (OH)-bending
1431	1420	1480	1478	*ῡ* (C–O) stretching
1542	1549	—	1550	*ῡ* (C=C) stretching
1702	1678	—	1638	*ῡ* (C=O) stretching
3287	3240	—	—	*ῡ* (OH)–carboxyl
—	—	—	Overlap in 3270	*ῡ* (OH)–carboxyl with hydrogen bonding
3497	3540	—	—	*ῡ* (OH)–hydroxyl
—	—	—	3415	*ῡ* (OH)–hydroxyl with hydrogen bonding
—	—	3256	3270	*ῡ* (OH)–alcohol
—	—	949	956	*ῡ* (C–N) stretching-ammonium structure
3063	Overlap in 3050	—	3023	*ῡ* (CH) stretching
—	—	2743–3018	2744–3019	*ῡ* (–CH_2_–), (–CH_3_) stretching
—	635	—	640	*ῡ* (Cu–O)

a Gallic acid.

b Choline chloride.

**Fig. 4:**
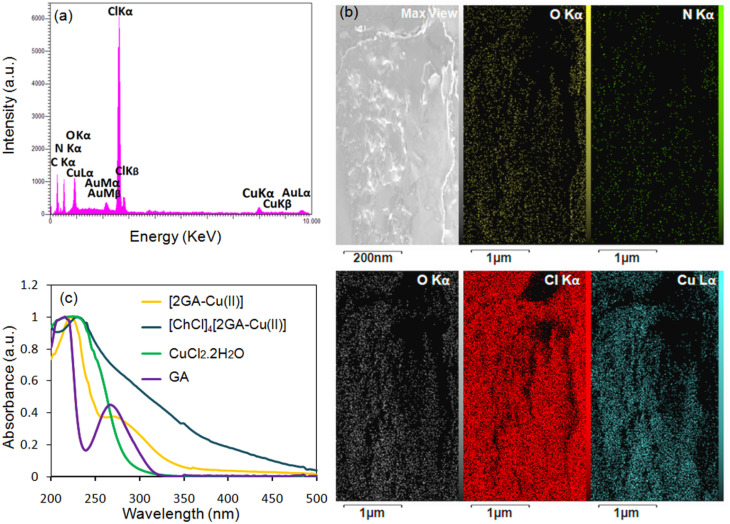
(a) EDX analysis; (b) mapping images of the constructing elements of [ChCl]_4_[2GA-Cu(ii)]; (c) UV-Vis normalized spectra of CuCl_2_·2H_2_O, GA, [2GA-Cu(ii)], and [ChCl]_4_[2GA-Cu(ii)] in deionized water (0.0005 M).

In addition, the EDX pattern and mapping analyses of [ChCl]_4_[2GA-Cu(ii)] were taken and illustrated precisely the presence of all constructing elements in the novel catalytic solvent structure, especially Cl and the Cu elements (Fig. 4a and b). Accordingly, this analysis provides a further sign for the production of Cu(ii)-ADES, as a novel multifunctional catalytic solvent system, in which Cu(ii) is responsible for the Lewis acidity property of this system.

The UV-Visible absorption spectra of CuCl_2_·2H_2_O, GA, [2GA-Cu(ii)], and [ChCl]_4_[2GA-Cu(ii)] were studied by UV-Vis spectrophotometer (Fig. 4c). The presence of the eminent absorption band at around 250 nm can be attributed to π → π* transitions from the full orbital of the phenolic oxygen to the d empty orbital of Cu(ii) ions, indicating the prosperous coordination of Cu(ii) ion and the ligand.^23^ Also, this peak in 2GA-Cu(ii) and [ChCl]_4_[2GA-Cu(ii)] spectra confirm that the valence of copper after immobilization on the gallic acid and finally in the related prepared Cu(ii)-ADES remains +2 and has not changed (Fig. 4c).

### 2.2 Physicochemical properties of [ChCl]_4_[2GA-Cu(ii)] DES

#### 2.2.1 Viscosity (*μ*)

**Fig. 5:**
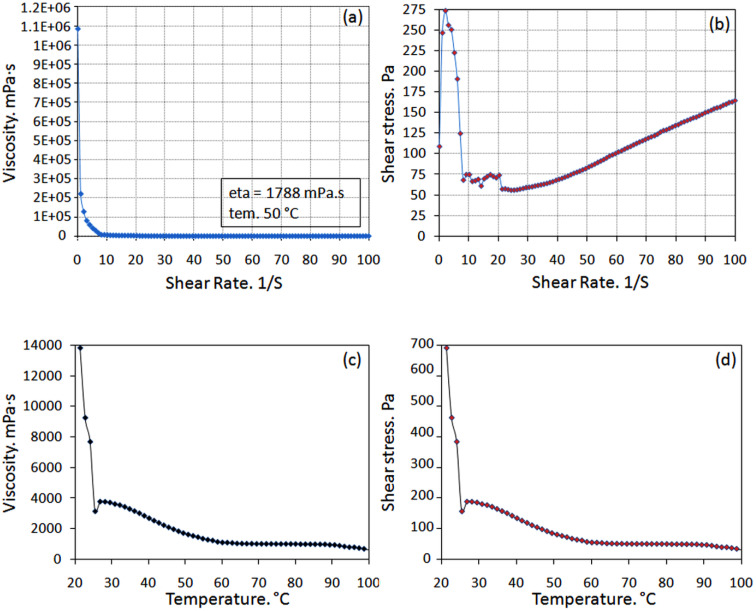
(a) Viscosity *versus* shear rate at 50 °C and (b) shear stress *versus* shear rate at 50 °C of [ChCl]_4_[2GA-Cu(ii)]; (c) dependence viscosity *versus* temperature, and (d) dependence shear stress *versus* temperature at shear rate 50 s^−1^ of [ChCl]_4_[2GA-Cu(ii)].

Similar to most ILs, the viscosity of DESs is a significant issue that should be noted. The chemical nature of the DES components, molar ratio, and temperature are generally affected by the viscosities of eutectic mixtures.^20*b*^ Almost all DESs represent high viscosities at r.t. which is often related to a vast hydrogen bonding network among components, resulting in lower mobility of free components within the DES. The forces such as electrostatic or van der Waals interactions and the large ion size and minimal void volume of most DESs may contribute to the high viscosity of DES. Fig. 5 reveals the viscosity data of [ChCl]_4_[2GA-Cu(ii)] at different temperatures and dependence viscosity *versus* temperature. The viscosity (*μ*) of most eutectic mixtures changes significantly due to the temperature. According to the obtained information on the viscosity–temperature profile, as a result of the temperature increases, the viscosity decreases (Arrhenius-like behavior) the viscosity of 3144 mPa s at 25 °C and 1788 mPa s at 50 °C for desired DES was measured (Table 2).

**Table 2:** Physicochemical properties of [ChCl]_4_[2GA-Cu(ii)] DES^a^

	Physicochemical properties					
	Density (*ρ*, g cm^−3^)	Viscosity (*μ*, mPa s)	Ionic conductivity (*k*, mS cm^−1^)	Refractive index^b^ (*n*_D_)	Melting point (°C)	pH^b^
Data	1.31	3144 (25 °C)	1.87 (25 °C)	1.3439	8	5.32
		1788 (50 °C)				

a Salt = ChCl, HBD = [ChCl]_4_[2GA-Cu(ii)], molar ratio of salt : HBD = 4 : 1.

b In solution (0.0025 M) of deionized water at 16.5 °C.

#### 2.2.2 Density (*ρ*)

The density was measured at 1.31 g cm^−3^ as one of the most significant physical properties of the DES as a solvent.

#### 2.2.3 Ionic conductivity (*K*)

Considering that the molar ratio of the HBD/organic salt dramatically affects the viscosities of DES, this parameter also significantly influences the conductivities of DESs.^17^ Also, with increasing the ChCl content, the conductivity of DES rises according to this evidence; it was measured as 1.87 mS cm^−1^.^24^

#### 2.2.4 Refractive index (*n*_D_)

The refractive index of [ChCl]_4_[2GA-Cu(ii)] solution (0.0025 M) in deionized water at 16.5 °C was determined at 1.3439 by refractometer, which means the speed of light in this solution is 1.3439 times lesser than the speed of light in a vacuum.

#### 2.2.5 Melting point (*m*)

The possible interactions occurring between ChCl and [2GA-Cu(ii)] lead to a dramatic reduction in the melting point at a scale of 8 °C of the mixture, an eutectic point at a determined molar ratio and thus formed a dark brown liquid system.

#### 2.2.6 pH

The pH value of [ChCl]_4_[2GA-Cu(ii)] DES in solution 0.0025 M of deionized water at 25 °C was measured at 5.32.

Finally, evaluating the exact content of decorated Cu particles in the [ChCl]_4_[2GA-Cu(ii)] ADES structure was taken through inductively coupled plasma atomic emission spectrometry (ICP-MS) analysis. As a result, the loading Cu particles were measured to be 9 mol% in 2 mL of ADES.

### 2.3 Evaluation of the effect [ChCl]_4_[2GA-Cu(ii)] as a M-ADES in the synthesis of 1,4-disubstituted 1,2,3-triazoles

For exploring the effect of the novel synthesized Cu(ii)-ADES as a multifunctional catalytic solvent system in the synthesis of 1,2,3-triazoles, the one-pot three-component click reaction benzyl bromide (1), sodium azide (2), and phenylacetylene (3a) were elected as the model reaction in selected reaction condition (catalyst (5 mol%), ADES (2 mL), temperature (55 °C), time (0.5 h)) to provide 1-benzyl-4-phenyl-1*H*-1,2,3-triazole (4a). The results, which have been registered in (Table 3), a range of catalysts, and various ADESs were employed, and all experiments were accomplished using stoichiometric amounts of the starting materials under atmospheric conditions.

**Table 3:** Evaluating of the effect catalyst, and comparison of the activity of diverse ADESs for the furnish 1-benzyl-4-phenyl-1*H*-1,2,3-triazole (4a)^a^

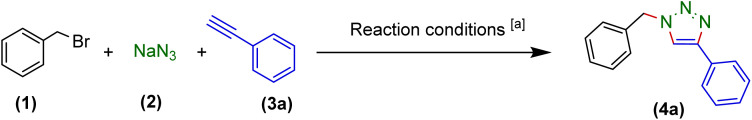			
Entry	Catalyst	ADES/(mL; *ρ* (g cm^−3^))	Yield^b^ (%)
1	CuCl_2_	[ChCl]_2_[GA]/(2; 1.14)	80
2	Ni(OAc)_2_	[ChCl]_2_[GA]/(2; 1.14)	70
3	ZnCl_2_	[ChCl]_2_[GA]/(2; 1.14)	68
4	None	[ChCl]_2_[GA]/(2; 1.14)	55
5	CuCl_2_	None	22
6	None	None	Trace
7	None	[ChCl]_4_[2GA-Cu(ii)]/(2; 1.31)	90
8	None	[ChCl]_4_[2GA-Ni(ii)]/(2; 1.37)	75
9	None	[ChCl]_4_[2GA-Zn(ii)]/(2; 1.34)	74

a Reaction conditions: benzyl bromide (1 mmol), sodium azide (1.5 mmol), phenylacetylene (1.2 mmol), catalyst (5 mol%), ADES (2 mL), temperature (55 °C), time (0.5 h).

b Isolated yield.

To begin the reaction optimization process, the model reaction was run in the presence of Ni(ii), Cu(ii), and Zn(ii) salts separately, and [ChCl]_2_[GA] as an ADES (Table 3, entries 1–3), which the best efficiency was observed with CuCl_2_ as Lewis acid (Table 3, entry 1). The reaction was then performed in the presence of [ChCl]_2_[GA] and the absence of any Lewis acid (Table 3, entry 4) to disclose the demand of the reaction to Cu(ii) species. The model reaction resulted in just 22% yield of the title product when the reaction was performed in the presence of CuCl_2_ without using [ChCl]_2_[GA] (Table 3, entry 5). Concerning the results gained from previous responses for the increase of ADES [ChCl]_2_[GA] performance and avoidance of direct use of metal salts and running the reaction in the heterogeneous media, we established a novel type of M-ADES as [ChCl]_4_[2GA-Cu(ii)] afterward, the model reaction was carried out in the presence of [ChCl]_4_[2GA-Cu(ii)] (Table 3, entry 7). It is clear that the present reactivity systems, compared to entry 1, approximately created no significant change in the desired product yield, Albeit the acceptable conversion was attained by executing the model reaction in the presence of [ChCl]_4_[2GA-Cu(ii)] without the utilization of any common solvents (Table 3, entry 7). Due to the excellent result obtained subsequently, the performance of [ChCl]_4_[2GA-Cu(ii)] was evaluated as a solvent-catalytic system and compared with other M-ADESs prepared from ChCl and [2GA-Ni(ii)], and [2GA-Zn(ii)] complexes (Table 3, entries 8 and 9). The results of these experiments revealed the supremacy of [ChCl]_4_[2GA-Cu(ii)] over the other M-ADESs synthesized (Table 3, entry 7). Thereupon, by discovering the best catalytic solvent system ([ChCl]_4_[2GA-Cu(ii)]) for the synthesis of 1-benzyl-4-phenyl-1*H*-1,2,3-triazole, we used the response surface methodology (RSM) for optimization. RSM is a method to evaluate the optimal situations in a multivariate system to achieve a maximum response rate. The most significant benefits of RSM can be included: (a) reduction in the number of tests and less time-consuming and save costs, (b) increase the accuracy and repeatability of tests, and (c) investigate the effect of different factors on each other and changing tendencies. Accordingly, to optimize the reaction conditions, RSM was used. To design experiments, changes in the input variables are made and then, the number of changes in the output response through the software is checked.^25^

### 2.4 Experimental design by central composite design (CCD)

Therefore, after determining the effectiveness of [ChCl]_4_[2GA-Cu(ii)] M-ADES (Table 3, entry 7), a central composite design (CCD) was applied to investigate the effects of experiential factors and their significant interactions exhibited in the experiments.^26^ So, a CCD was utilized for three parameters to design experiments. As summarized in Table 4, three-level of three independent process variables such as [ChCl]_4_[2GA-Cu(ii)] (mL), temperature, and time were selected by Box–Behnken. Next, to design production by choosing the target amount of factors and the highest yield of the desired product, also introducing two repetitions from the central point as the input into the Design-Expert software (version 13) eventually designed for us fourteen experiments as the output (Table 5).

**Table 4:** Experimental range and levels of independent process variables by Box–Behnken

Variables	Unit	Symbol coded	Levels		
			−1	0	+1
Time	h	A	0.75	1	1.25
[ChCl]_4_[2GA-Cu(ii)]	mL	B	1	2	3
Temperature	°C	C	40	50	60

**Table 5:** Optimization of parameters using response surface methodology for the synthesis of 1-benzyl-4-phenyl-1*H*-1,2,3-triazole (4a)^a^

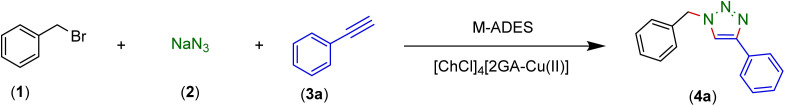				
Run	A: time (h)	B: [ChCl]_4_[2GA-Cu(ii)] (mL)/(mol% of Cu)	C: temperature (°C)	Yield^b^ (%)
1	0.75	2/9	40	82
2	1	3/13.5	60	84
3	0.75	1/4.5	50	70
4	0.75	3/13.5	50	86
**5**	**1**	**2/9**	**50**	**98**
6	1	1/4.5	60	75
7	0.75	2/9	60	88
8	1.25	2/9	60	95
9	1	3/13.5	40	82
10	1	1/13.5	40	69
11	1.25	1/4.5	50	78
**12**	**1**	**2/9**	**50**	**98**
13	1.25	2/9	40	89
14	1.25	3/13.5	50	89

a Reaction conditions: benzyl bromide (1 mmol), sodium azide (1.5 mmol), phenylacetylene (1.2 mmol).

b Isolated yield.

**Fig. 6:**
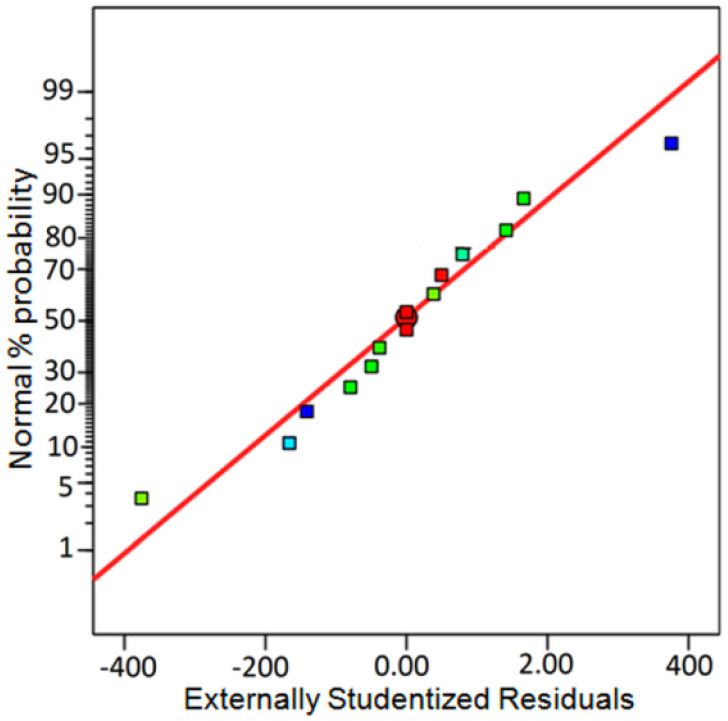
A normal plot of residuals for the reaction optimization.

Experiments were performed, and the desired product efficiency was calculated in each of the conditions; the fit of the results obtained in the laboratory scale with the results predicted by the software (adjusted *R*_2_ = 0.9959, sequential *p*-value < 0.0001) is shown in Fig. 6. The best yield for the model reaction was obtained in [ChCl]_4_[2GA-Cu(ii)] (2 mL), time (1 h), and temperature 50 °C (entries 5 and 12) and was considered as the best reaction condition.

**Fig. 7:**
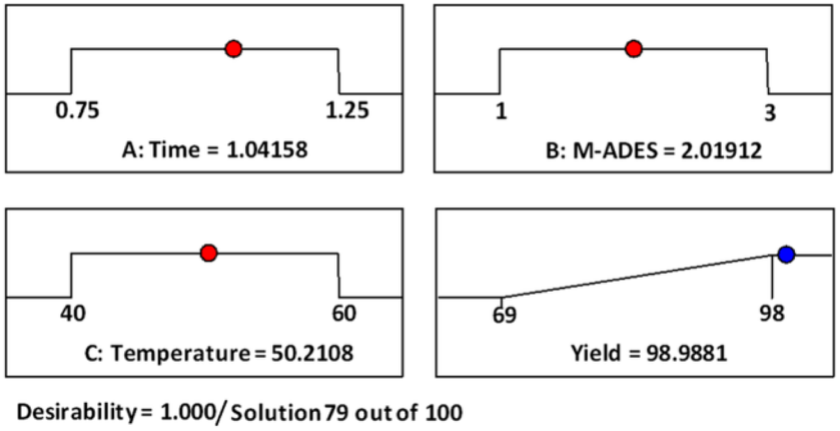
Adjusted numerical optimization for maximizing the desired product production by Design-Expert software.

In fact, ramps are preferred to visualize the optimization results by researchers, as shown in Fig. 7 these are a sample of numerical optimization for maximizing desired product production. Accordingly, by accepting the values of the factors to the laboratory conditions and performing the test, no exceptional differences were found between experimental data and predicted data by Design-Expert software, and can be reliably expressed simulation results. As a result, the conditions time (1 h), [ChCl]_4_[2GA-Cu(ii)] (2 mL), 50 °C with two repetitions entries were recorded as optimal conditions for the continuing process. Due to the excellent result observed from the [ChCl]_4_[2GA-Cu(ii)] catalytic solvent system to enhance the scope of reactants and the synthesis of novel high-performance triazole compounds, at first, we decided to synthesize a series of terminal alkynes of aromatic/aliphatic amines and caprolactam. In this way, we applied an impressive K_2_CO_3_-promulgated method for the *N*-alkynation of 5*H*-dibenzo[*b*,*f*]azepine and *N*-ethylaniline (3b and 3d) as well as *N*-alkynation of diphenylamine and caprolactam (3c and 3e) by NaNH_2_ as a strong base by propargyl bromide in dry *N*,*N*-dimethylformamide (DMF) at room temperature for 4 to 5 hours to procuring desired compounds in good yields (Table S1 in ESI,†3b–e).

**Scheme 3:**
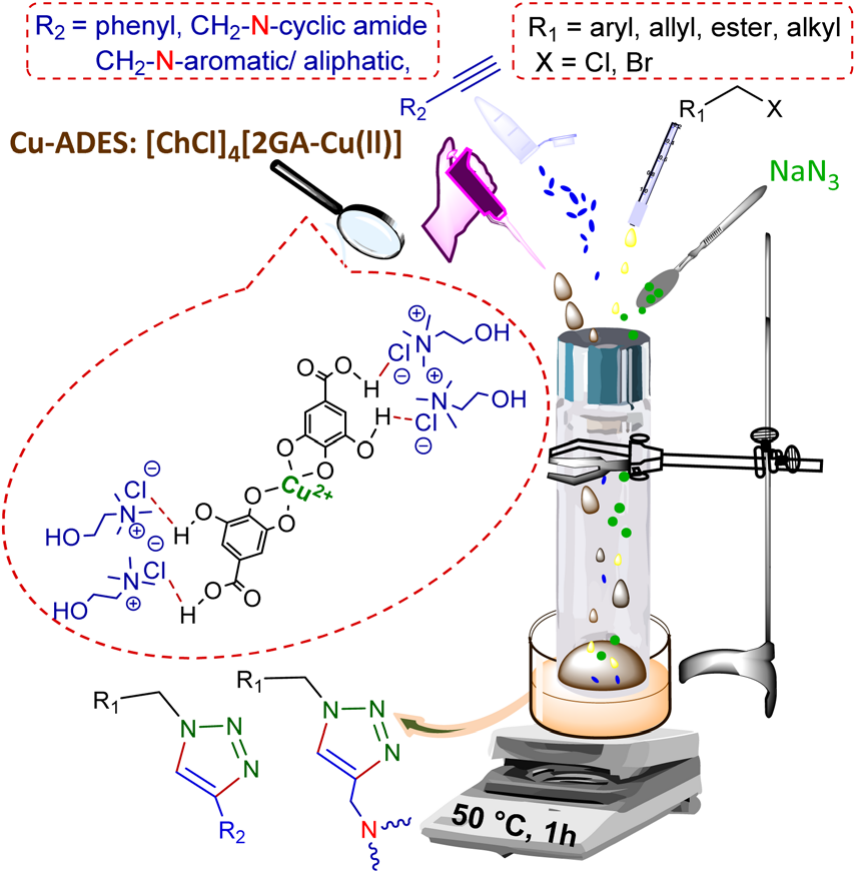
A general illustration of preparation novel 1,4-disubstituted 1,2,3-triazole frameworks.

Therefore, phenylacetylene (3a) and synthesized terminal *N*-alkynes (3b–e) in the presence of NaN_3_ and aryl/alkyl/allyl and ester halides provided the novel various 1,4-disubstituted 1,2,3-triazoles preparation under the optimized reaction conditions (M-ADES: 2 mL, time: 1 h, temperature: 50 °C) in the present [ChCl]_4_[2GA-Cu(ii)] M-ADES as solvent/catalyst system (Table 6 and Scheme 3).

**Table 6:** The synthesis of novel 1,4-disubstituted 1,2,3-triazoles by [ChCl]_4_[2GA-Cu(ii)]^a^

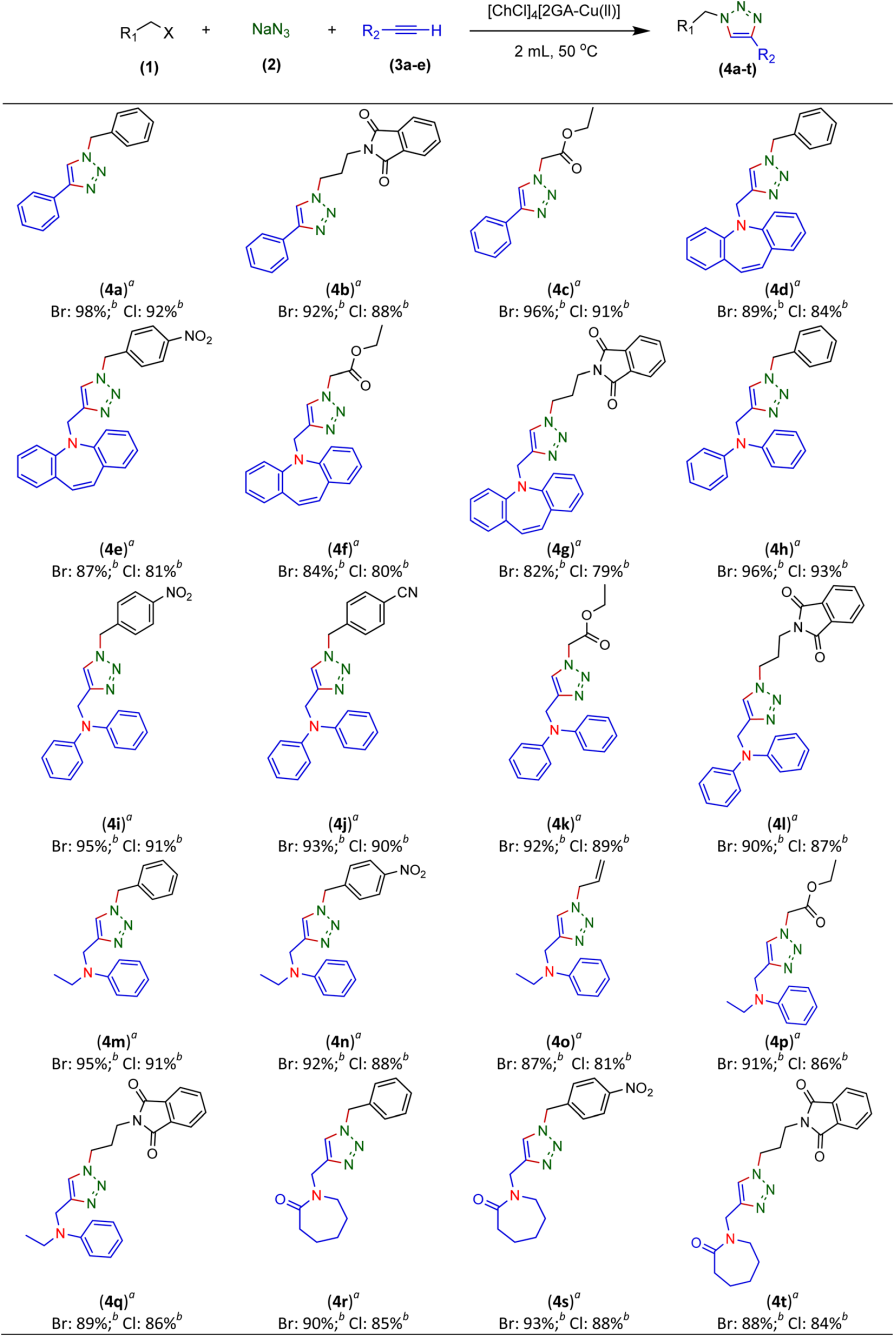

a Reaction conditions: terminal alkyne (1.2 mmol), benzyl, allyl, and alkyl halides (1 mmol), sodium azide (1.5 mmol), [ChCl]_4_[2GA-Cu(ii)] (2 mL), 50 °C.

b Isolated yield.

With the optimized conditions in hand and various types of terminal alkynes, for the finding of the potential scope and versatility of reagents for this CuAAC one-pot three-component procedure, under very favorable situations, different types of benzyl, allyl, alkyl, and ester halides were used as the substrates for the production of 1,4-disubstituted 1,2,3-triazoles which the outcomes are rendered in Table 6. In all cases, expected triazoles 4a–t were acquired in high yields (up to 98%) after an ordinary purification process exhibiting an extensive scope and high endurance of functional groups in the reaction media. We first treated structurally diverse halides with phenylacetylene and NaN_3_, which reacted with excellent efficiency (Table 6, 4a–4c in 92–98% yields), as bromine halides behaved very excellent, while the idem triazoles 4a–4c were generated in 88–91% yields with the less active chlorine halides. Afterward, to exhibit reaction generality, the terminal *N*-alkynes derived (Table S1 in ESI,†3b–e) were investigated to give all the desired triazoles (Table 6, 4d–t) in good to high yields regardless of different functional groups. On the other hand, ester and allyl functional groups sustained allowed us to acquire the desired products (4c, f, k, o, and p), which can be benefited as scaffolds for complex structures. Besides, the existence of benzyl halides with electron-withdrawing (–NO_2_, –CN) groups (4e, i, j, and n) led to better efficiency in comparison with the propyl-isoindoline-1,3-dione group (4b, f, k, o, and p) (Table 6).

Notwithstanding the above impressive results, the utilization of the presented efficacious strategy was examined for the preparation of *N*-((1-(4-nitrobenzyl)-1*H*-1,2,3-triazole-4-yl)methyl)-*N*-phenylaniline and 4-((4-((diphenylamino)methyl)-1*H*-1,2,3-triazole 1-yl)methyl)benzonitrile in several scales-up (1, 10, and 50 mmol) comprising a nitro/nitrile group that can be transformed into an amine functional group with high performance in organic synthesis and pharmaceutical industries also as appropriate functional groups in other organic reactions. Furthermore, diphenylamine derivatives and concatenation of cyclic triazole into diphenylamine would modify the physicochemical functions and acquire applications in the scope of biology and material science.^27^ It is noteworthy that the registered outputs in Table 7 manifested the supreme capacity and ability of [ChCl]_4_[2GA-Cu(ii)] to be applied in chemical industrial manufacturers in the future.

**Table 7:** Evaluation of the proposed strategy in several scales-up

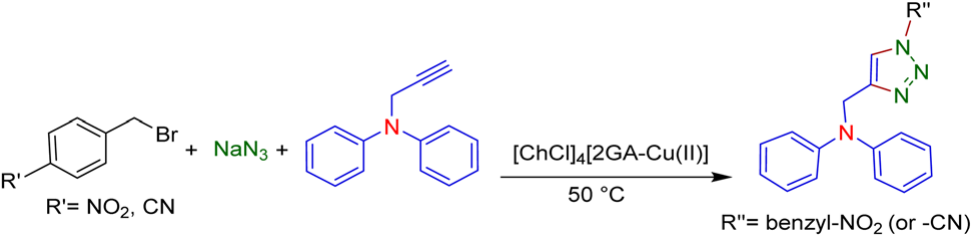				
Substrates	Aryl halide	Amount of aryl halide (mmol)	ADES (mL)	Yield^d^ (%)
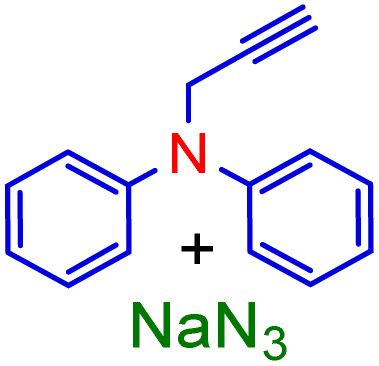	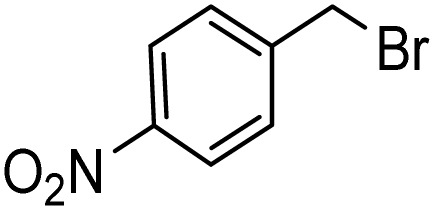	1	2	95^a^
		10	5	92^b^
		50	15	89^c^
	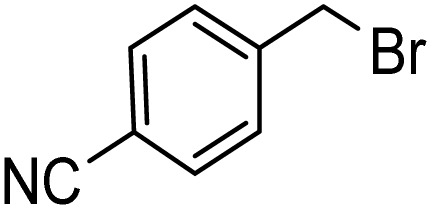	1	2	93^a^
		10	5	90^b^
		50	15	88^c^

a Reaction conditions: *N*-phenyl-*N*-(prop-2-yn-1-yl)aniline (1.2 mmol), sodium azide (1.5 mmol).

b Reaction conditions: *N*-phenyl-*N*-(prop-2-yn-1-yl)aniline (12.5 mmol), sodium azide (15.5 mmol).

c Reaction conditions: *N*-phenyl-*N*-(prop-2-yn-1-yl)aniline (62.5 mmol), sodium azide (77.5 mmol).

d Isolated yield.

**Fig. 8:**
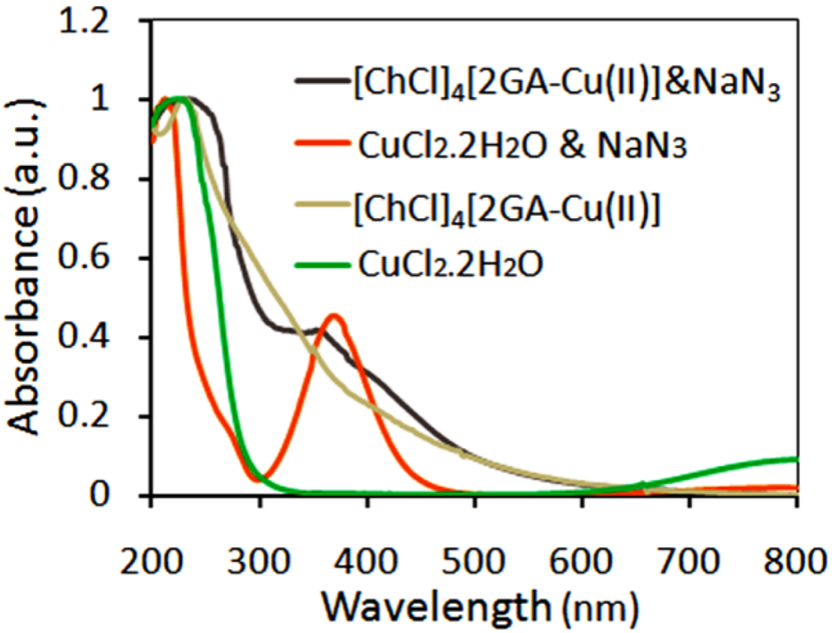
UV-Vis normalized spectra of CuCl_2_ & NaN_3_, [ChCl]_4_[2GA-Cu(ii)] & NaN_3,_ [ChCl]_4_[2GA-Cu(ii)], and CuCl_2_·2H_2_O in deionized water (0.0005 M).

To prove the Cu(ii)-ADES performance in this process, we have recorded in Fig. 8 the UV-Vis spectra for fresh [ChCl]_4_[2GA-Cu(ll)] and CuCl_2_ treated with NaN_3_ to ascertain confirmation of the oxidation state of Cu(ii) to Cu(i) by sodium azide in the present protocol. In both UV-Vis spectra, the corresponding absorptions of Cu(i) in 300–425 nm wavelength and maximum intensity in wavelength of 270 nm appeared, which demonstrates Cu(i) is present in both spectra, according to the results collected from the UV absorption spectrum was determined that after treatment with NaN_3,_ Cu(ii) gets reduced to Cu(i) state.^28^

**Scheme 4:**
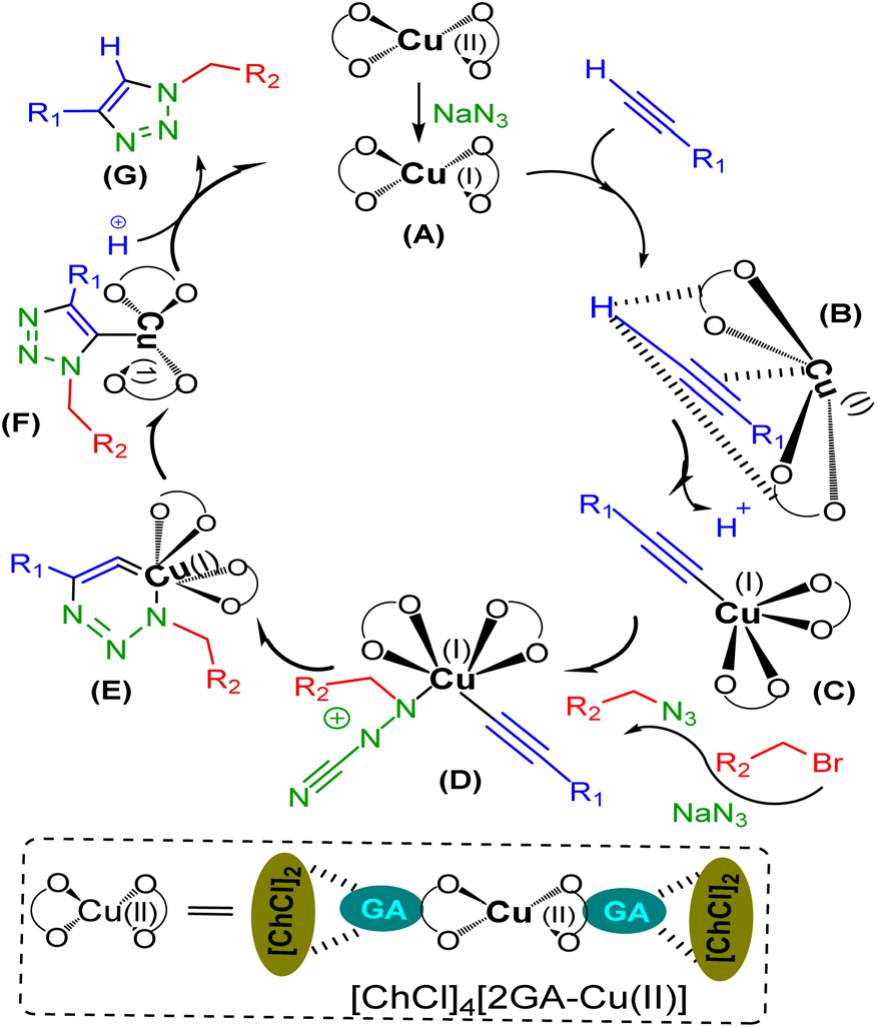
Proposed mechanism of one-pot CuAAC reaction using [ChCl]_4_[2GA-Cu(ii)] for the regioselective formation of 1,4-disubstituted 1,2,3-triazoles.

It is not possible to present the reaction mechanism in detail according to the complexity and novelty of the catalytic effect of DESs, but considering the experiments done and the prior reports in the literature, a plausible mechanism has been proposed for the above-mentioned construction of 1,4-disubstituted 1,2,3-triazoles of aryl alkyl amines *via* CuAAC without using an extra reducing agent, as has been illustrated in Scheme 4. In primary, reducing Cu(ii) to Cu(i) of catalyst will occur by sodium azide, as one of the required starting materials to provide A.^26^ Then, the acetylene-activated complex B is formed through the interaction with the hydrogen bond acceptors groups of DES to loss a proton in the absence of base and provide the copper complex C.^12^ The nucleophilic attack on C by the *in situ* generated organic azide is led to the intermediate D, which undergoes an intermolecular cycloaddition reaction to give the isomer E.^29^ Protonolysis of F, which is formed in turn *via* the rearrangement reaction in E, creates the desire triazole H and regenerates Cu(i) species.

**Fig. 9:**
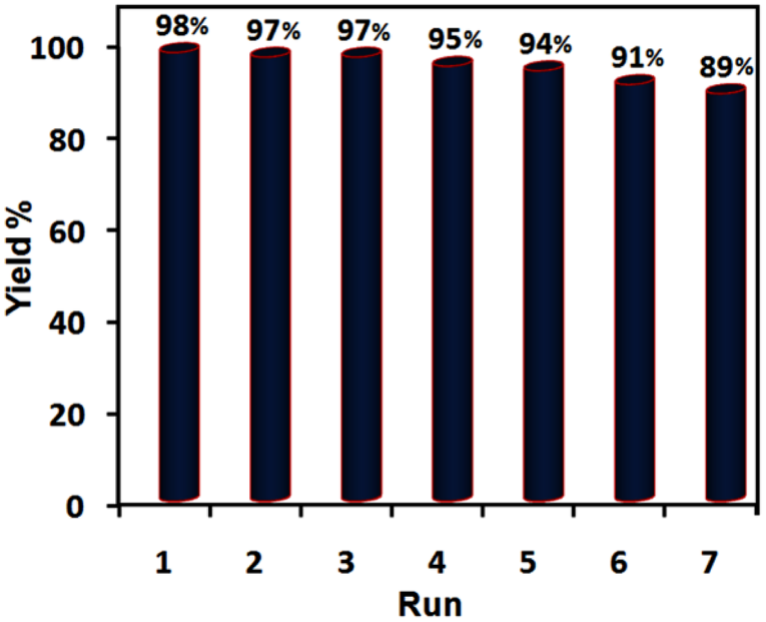
Evaluating recyclability and reusability of [ChCl]_4_[2GA-Cu(ii)] in the synthesis of 1-benzyl-4-phenyl-1*H*-triazole under the optimized conditions.

Confidently, the durability and recyclability of the catalytic systems are very significant in terms of being eco-friendly and economically at the industrial level and drafting efficient and green synthetic processes due to lessening detrimental environmental and cost-effectiveness impacts. For this purpose, we evaluated these factors of [ChCl]_4_[2GA-Cu(ii)] as a solvent/catalyst system in the optimized reaction conditions, as shown in Fig. 9.

Therefore, in the [ChCl]_4_[2GA-Cu(ii)] recycling experiment, after completing the reaction in each round, diethyl ether (2 × 15 mL) was added to the reaction media to extract all remaining precursors and products. Then [ChCl]_4_[2GA-Cu(ii)] dissolved in 10 mL water and was simply recovered by extraction with ethyl acetate. Finally, Cu-ADES was dehumidified by evaporation of the organic layer under vacuum conditions at 70 °C for 1 h and then reused in the next run. According to the related outputs illustrated in Fig. 9 an insignificant decrease in the intended product yield after seven successive cycles. So, these data indicate that the catalytic activity of [ChCl]_4_[2GACu(ii)] has been maintained after seven consecutive cycles.

## 3. Experimental section

All the information is perfectly presented in the ESI file.†

## 4. Conclusions

In summary, a novel and green three-component metal acidic deep eutectic solvent, Cu(ii)-ADES, bearing copper salt, gallic acid, and choline chloride, was synthesized and fully identified by physicochemical techniques as a solvent/catalyst system. This benign and cost-effective M-ADES exhibited excellent reactivity when it was served for creating a novel library of 1,4-disubstituted 1,2,3-triazoles *via* three-component click reactions in base-free and reducing agent-free conditions from a variety of benzyl, allyl, ester, and alkyl halides, sodium azide, and terminal alkynes, which mainly were manufactured of various aromatic and aliphatic amines as well as caprolactam. This catalyst/solvent system, [ChCl]_4_[2GA-Cu(ii)], was also recyclable and reusable without considerable loss in its activity after seven consecutive runs.

Consequently, herein, in addition to the establishment of a novel family of DES, it has been possible to find a synthetic methodology using this M-ADES which satisfies most of the economic and environmental aspects in the green chemical industry by including these reliable features: (a) inexpensive, reusable, and ecological friendliness multifunctional M-ADES in base-free condition, (b) easily accessible aryl/alkyl amines and cyclic amide as highly beneficial precursors for terminal alkynes, and ultimately, (c) development of practical extensive scale-up synthesis to procure 1,4-disubstituted 1,2,3-triazoles with nitro, nitrile, allyl, and ester functional groups that will be useful for subsequent synthetic applications.

## Author contributions

Nastaran Bagherzadeh: conceptualization, methodology, software, validation, formal analysis, investigation, resources, data curation, writing-original draft, visualization. Mohammad Amiri: investigation, resources. Ali Reza Sardarian: conceptualization, project administration, resources, funding acquisition, writing-review & editing.

## Conflicts of interest

There are no conflicts to declare.

## Supplementary Material

RA-013-D3RA06570G-s001
