# Supplementary material for: Novel Cu(ii) acidic deep eutectic solvent as an efficient and green multifunctional catalytic solvent system in base-free conditions to synthesize 1,4-disubstituted 1,2,3-triazoles
Source: RSC Adv. 2023 Dec 14;13(51):36403–15. doi: 10.1039/d3ra06570g (PMC10719904; doi:10.1039/d3ra06570g)
Supplement: RA-013-D3RA06570G-s001 [file RA-013-D3RA06570G-s001.pdf]

Electronic Supplementary Material (ESI) for RSC Advances.
This journal is © The Royal Society of Chemistry 2023

Supporting Information for

Novel Cu(II) Acidic Deep Eutectic Solvent as an Efficient and Green Multifunctional Catalytic Solvent System in Base-Free Conditions to Synthesize 1,4-Disubstituted 1,2,3-Triazoles

Nastaran Bagherzadeh, [a] Mohammad Amiri [a] and Ali Reza Sardarian *[a]

[a] *Chemistry Department, College of Sciences, Shiraz University, Shiraz, Iran.*

*Corresponding author Fax number: +987136460788; Tel.: +987136137107

*E-mail: sardarian@shirazu.ac.ir

Experimental section

Chemical and instrumentation

All commercial chemical substances were purchased with high purity from Sigma-Aldrich and Merck Chemical firms. Designation of the progress of the reactions and the purity of the compounds was attained by thin-layer chromatography (TLC) on silica gel polygram SILG/UV 254 plates. Silica gel (254 mesh) was utilized for column chromatography. The desired products by comparing their spectral and physical data with existing data in the literature were recognized, which included FT-IR, NMR, melting point, UV–Vis, and EDX. XRD pattern through an X-ray Diffractometer Bruker D2 Phaser with Cu-Kα radiation (λ = 1.54184 A) was recorded. FT-IR spectral data were obtained by model Shimadzu FT-IR 8300 using a KBr pellet. NMR spectra were recorded in $CDCl_3$ using Bruker Avance at 400 MHz (^1H) and 101 MHz (^{13}C {^1H}) at 25 °C. A micro melting point apparatus (electrothermal, BUCHI 510) was applied to measure melting points. UV–Vis spectra were recorded on a PerkinElmer Lambda 25 UV–Vis spectrometer. The chemical composition and elements in the $[ChCl]_4$[2GA-Cu(ll)] were identified using an energy dispersive X-ray (EDX) spectroscopy paired with the Philips scanning electron microscopy (SEM). The determination of pH was performed using the DT-PH1100 Benchtop pH meter. The viscosity was estimated using a Modular Compact Rheometer (MCR 302) Anton Paar. The refractive index was obtained by A. *Krüss Optronic* GmBh refractometer. The measure of ionic conductivity was carried out using a conductivity meter 712.

Synthesis of the [2GA-Cu(II)] complex:

According to the literature report, [1] a 50mL round-bottomed flask was charged with the aqueous mixture (10 mL) of GA (10 mmol, 1.70 g) and then 10 mL of the aqueous solution of $CuCl_2.2H_2O$ (5 mmol, 0.85 g) at room temperature. Then NaOH (0.1 M) was added slowly to the mixture until it turned black and reached pH 7.5. After 4 h of constant stirring, the synthesized [2GA-Cu(II)] complex was collected from the reaction by centrifugation, washed with ethanol (10 mL × 1) to remove waste materials, and dried at 50 °C under vacuum. The mol% amount of copper in 2 mL of $[ChCl]_4$[2GA-Cu(II)] was measured by inductively coupled plasma (ICP, Varian, Vista- pro).

Preparation of $[ChCl]_4$[2GA-Cu(II)] as a metal acidic deep eutectic solvent:

For the preparation of this novel metal acidic deep eutectic solvent, using the previous procedure reported for the synthesis of choline chloride-based DES, [2a-c] in a flask, choline chloride (40 mmol, 5,56 g) and [2GA-Cu(II)] (10 mmol, 4 g) were mixed and next heated at 100 °C until forming a dark brown liquid. Cooling down the mixture to room temperature provides the desired pure M-ADES without requiring further purification.

Synthesis of starting materials

Synthesis of 5-(prop-2-yn-1-yl)-5*H*-dibenzo[b,f]azepine (3b) and *N*-ethyl-*N*-(prop-2-yn-1-yl)aniline (3d):

To the solution of an amine (10 mmol) in dry dimethylformamide (20 mL) was added K_2CO_3 (20 mmol) slowly under nitrogen atmosphere at 0 °C and let the mixture stir for 0.5 h at room temperature. Afterward, propargyl bromide (1.34 mL, 15 mmol) was slowly added to the reaction

media, and the resultant mixture was stirred continuously at room temperature for 4h. After which, the reaction mixture with 10 mL of water was quenched and ethyl acetate (3 × 10 mL) was extracted then the organic layer over anhydrous Na_2SO_4 was dried. Finally, the pure desired product was afforded after solvent evaporation and purification by silica gel column chromatography (n-hexane: ethyl acetate/ (30: 1)).

Synthesis of *N*-phenyl-*N*-(prop-2-yn-1-yl) aniline (3c) and 1-(prop-2-yn-1-yl)azepan-2-one (3e):

To a solution of 10 mmol of Caprolactam (or diphenylamine) in dry dimethylformamide (20 mL) was added slowly, $NaNH_2$ (12 mmol) at 0 °C under atmosphere nitrogen, and the suspension was let to be stirred for 0.5 h at the same temperature. Then propargyl bromide (1.07 mL, 12 mmol) was added dropwise to the reaction media at 0 °C. Afterward, the resultant mixture to room temperature was warmed and stirred constantly for 4 h. The reaction mixture by NH_4Cl saturated aqueous solution (10 mL) was quenched, extracted with ethyl acetate (3 × 40 mL), finally dried over anhydrous Na_2SO_4.

Table S1 The synthetic pathway terminal alkynes of amines precursors.

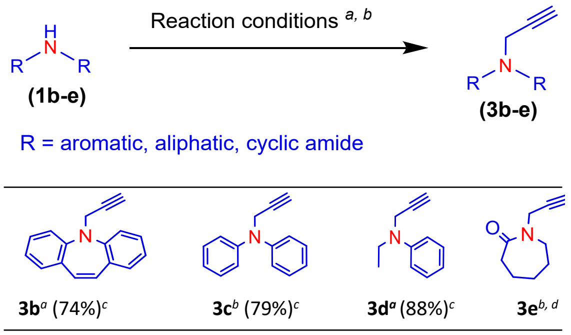

[a] Reaction conditions: DMF, K_2CO_3, 0 °C, 0.5 h; propargyl bromide, r.t. [b] Reaction conditions: DMF, $NaNH_2$, 0 °C, 0.5 h; propargyl bromide, r.t. [c] Isolated yield. [d] The desired product was considered without purification.

General procedure for preparation of 1,4-disubstituted 1,2,3-triazoles:

A 15 mL flask was charged with terminal alkyne (1.2 mmol), sodium azide (1.5 mmol), aliphatic halide (1 mmol), and $[ChCl]_4[2GA\text{-}Cu(II)]$ (2 mL). The resultant for an appropriate time at 50 °C was stirred, which is determined by TLC analysis. After completing the reaction, the mixture was extracted with ethyl acetate (2 × 15 mL). Then residue was diluted with water(10mL) and extracted with ethyl acetate (10 mL) to separate the remained product, and behind it $[ChCl]_4[2GA\text{-}Cu(II)]$ was isolated. The ethyl acetate extracts were combined and dried with Mg_2SO_4 and filtered. Afterward, the filtrate was concentrated by a rotatory evaporator, and the residue on silica gel using n-hexane/ethyl acetate (10: 4) as the eluent, by column chromatography was purified.

Physical data

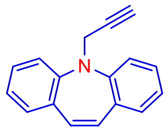

5-(Prop-2-yn-1-yl)-5*H*-dibenzo[b,f]azepine (**3b**)

(Yield: 74%; 0.171 mg; Yellowish green solid), m.p. 88 °C (Literature report [3], 88-90 °C); ^{1}H NMR ($CDCl_3$, 400 MHz): δ 2.33 (t, J = 4 Hz, CH), 4.54 (d, J = 4 Hz, CH_2), 6.82 (s, 2H, aromatic ring), 7.12 (t, J = 6 Hz, 2H, aromatic ring), 7.17 (d, J = 8 Hz, 2H, aromatic ring), 7.29 (d, J = 8 Hz, 2H, aromatic ring), 7.36 (t, J = 8 Hz, 2H, aromatic ring) (ppm); ^{13}C{1H} NMR ($CDCl_3$, 101 MHz): δ 150.21, 133.52, 132.29, 129.47, 128.95, 124.05, 120.28, 80.87, 73.02, 41.42 (ppm); Anal. Calcd for $C_{17}H_{13}N$, C, 88.28; H, 5.67; N, 6.06%; Found C, 88.27; H, 5.65; N, 6.09%.

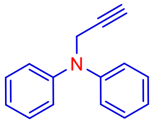

N-Phenyl-*N*-(prop-2-yn-1-yl)aniline (**3c**)

(Yield: 79%; 0.164 mg; Pale yellow oil); 1H NMR ($CDCl_3$, 400 MHz): δ 2.30 (t, J = 4 Hz, CH), 4.49 (d, J = 4 Hz, CH_2),7.10 (t, J = 8 Hz, 2H, aromatic ring), 7.18 (d, J = 8 Hz, 4H, aromatic ring), 7.38 (t, J = 8 Hz, 4H, aromatic ring) (ppm); ^{13}C{^{1}H} NMR ($CDCl_3$, 101 MHz): δ 147.47, 129.37, 122.30, 121.32, 79.99, 72.40, 42.16 (ppm) [4]; Anal. Calcd for $C_{15}H_{13}N$, C, 86.92; H, 6.32; N, 6.76%; Found C, 86.90; H, 6.30; N, 6.80%.

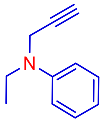

N-Ethyl-*N*-(prop-2-yn-1-yl)aniline (**3d**)

(Yield 88%; 0.140 mg; Pale yellow oil); ^{1}H NMR ($CDCl_3$, 400 MHz): δ 1.15 (t, J = 8 Hz, CH_3), 2.11 (t, J = 4 Hz, CH), 3.38 (q, J = 8 Hz, CH_2), 3.96 (d, J = 4 Hz, CH_2), 6.71 (t, J = 8 Hz, 1H, aromatic ring), 6.79 (d, J = 4 Hz, 2H, aromatic ring), 7.17 (t, J = 8 Hz, 2H, aromatic ring) (ppm); ^{13}C{^{1}H} NMR ($CDCl_3$, 101 MHz): δ 147.85, 129.20, 122.85, 113.93, 45.60, 39.58, 31.44, 30.19, 12.41(ppm) [5]; Anal. Calcd for $C_{11}H_{13}N$, C, 82.97; H, 8.23; N, 8.80%; Found C, 82.94; H, 8.20; N, 8.86%.

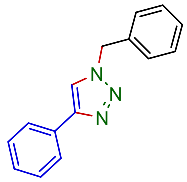

1-Benzyl-4-phenyl-1*H*-1,2,3-triazole (**4a**)

(Yield: 98%; 0.231 mg; White solid), m.p. 129-130 °C (Literature report [6], 128-130 °C); 1H NMR ($CDCl_3$, 400 MHz): δ 5.80 (s, CH_2), 7.34 (t, J = 6 Hz, 2H, aromatic ring), 7.40 (m, 5H, aromatic ring), 7.89 (s, 3H, aromatic ring) (ppm); $^{13}C\{^1H\}$ NMR ($CDCl_3$, 101 MHz): δ 134.66, 129.17, 128.87 (2C), 128.84 (2C), 128.20 (3C), 128.13 (4C), 125.62, 54.49 (ppm); FT-IR (KBr): $\bar{\upsilon}$ 3441 (w), 3123-3038 (w), 2951 (vw), 2432 (vw), 2274 (vw), 1960 (vw), 1601 (w), 1467-1443 (m), 1353 (w), 1334 (w), 1280 (vw), 1263 (vw), 1224 (s), 1206 (m), 1191-1138 (w), 1076 (m), 1050 (m), 975 (w), 827 (w), 780 (w), 767 (vs), 729 (vs), 695 (vs), 581 (w), 510 (m), 474 (w) (cm^{-1}); Anal. Calcd for $C_{15}H_{13}N_3$, C, 76.57; H, 5.57; N, 17.86%; Found C, 76.55; H, 5.55; N, 17.90%.

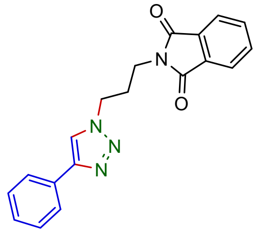

2-(3-(4-Phenyl-1*H*-1,2,3-triazol-1-yl)propyl)isoindoline-1,3-dione (**4b**)

(Yield 92%; 0.306 mg; White solid), m.p 144-145 °C (Literature report [7], 144-146 °C); ^1H NMR ($CDCl_3$, 400 MHz): δ 2.33 (q, J = 8 Hz, CH_2), 3.73 (t, J = 8 Hz, CH_2),4.40 (t, J = 8 Hz, CH_2), 7.19 (s, 1H, aromatic ring), 7.26 (t, J = 6 Hz, 1H, aromatic ring), 7.36 (t, J = 8 Hz, 2H, aromatic ring), 7.67 (m, 2H, aromatic ring), 7.78 (m, 2H, aromatic ring), 7.93 (s, 1H, aromatic ring) (ppm); $^{13}C\{^1H\}$ NMR ($CDCl_3$, 101 MHz): δ 168.46 (2C), 147.73, 134.26 (2C), 131.92, 128.83 (2C), 128.16, 125.77 (2C), 123.46 (2C), 120.32, 47.91, 35.07, 29.45 (ppm); FT-IR (KBr): $\bar{\upsilon}$ 3454 (w), 3143 (vw), 3125 (vw), 3091 (w), 3064 (vw), 3037 (vw), 2936 (w), 2855 (vw), 2372 (vw), 1768 (w), 1715 (vs), 1702 (vs), 1443 (w), 1432 (w), 1399 (s), 1370 (m), 1340 (w), 1188-1048 (w), 1028 (m), 978 (vw), 887 (w), 813 (vw), 784 (vw), 767 (m), 716 (m), 696 (w), 614 (vw), 531 (w), 512 (w) (cm^{-1}); Anal. Calcd for $C_{19}H_{16}N_4O_2$, C, 68.66; H, 4.85; N, 16.86%; Found C, 68.65; H, 4.82; N, 16.87%.

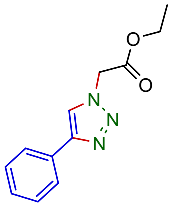

Ethyl 2-(4-phenyl-1*H*-1,2,3-triazol-1-yl)acetate (**4c**)

(Yield 96%; 0.222 mg; White solid), m.p. 104-106 °C (Literature report [8], 102-104 °C); ^{1}H NMR ($CDCl_3$, 400 MHz): δ 1.34 (t, *J* = 8 Hz, CH_3), 4.31 (q, *J* = 8 Hz, CH_2), 5.23 (s,CH_2), 7.37 (t, *J* = 8 Hz, 1H, aromatic ring), 7.46 (t, *J* = 8 Hz, 2H, aromatic ring), 7.87 (d, *J* = 8 Hz, 2H, aromatic ring), 7.94 (s, 1H, aromatic ring) (ppm); $^{13}C\{^1H\}$ NMR ($CDCl_3$, 101 MHz): δ 166.28, 148.30, 130.36, 128.86 (2C), 128.31, 125.84 (2C), 120.93, 62.49, 50.98, 14.09 (ppm); FT-IR (KBr): $\bar{\upsilon}$ 3473 (w), 3418 (m), 3136 (w), 3104 (vw), 2993 (vw), 2971 (vw), 2949 (vw), 2374 (vw), 1756 (vs), 1628 (w), 1468 (m), 1443 (m), 1414-1266 (w), 1216 (vs), 1200 (vs), 1146 (m), 1099 (w), 1077 (m), 1051 (w), 1017 (m), 978 (w), 874 (w), 826 (w), 767 (s), 710 (w), 695 (m), 673 (vw), 578 (w), 513 (w), 463 (w) (cm^{-1}); Anal. Calcd for $C_{12}H_{13}N_3O_2$, C, 62.33; H, 5.67; N, 18.17%; Found C, 62.31; H, 5.65; N, 18.18%.

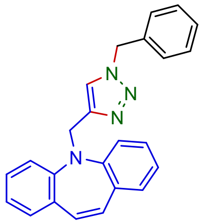

5-((1-Benzyl-1*H*-1,2,3-triazol-4-yl)methyl)-5*H*-dibenzo[b,f]azepine (**4d**)

(Yield 89%; 0.324 mg; Cream solid), m.p. 129-139 °C; 1H NMR ($CDCl_3$, 400 MHz): δ 5.02 (s, CH_2), 5.29 (s, CH_2), 6.63 (s, 2H, aromatic ring), 6.81 (m, 2H, aromatic ring), 6.89 (t, *J* = 6 Hz, 2H, aromatic ring), 6.97 (d, *J* = 8 Hz, 2H, aromatic ring), 7.00 (d, *J* = 4 Hz, 2H, aromatic ring), 7.15 (m, 5H Hz, aromatic ring) (ppm); $^{13}C\{^1H\}$ NMR ($CDCl_3$, 101 MHz): δ 134.93 (2C), 133.45, 132.02 (2C), 129.15, 129.05 (2C), 128.90 (2C), 128.29 (2C), 127.19 (4C), 123.85, 123.82, 123.02 (2C), 120.57 (2C), 53.80, 47.42 (ppm); FT-IR (KBr): $\bar{\upsilon}$ 3060 (w), 3018 (s), 2906 (vs), 2774 (vw), 2422 (vw), 1957 (vw), 1591 (m) 1483 (vs), 1459 (vs), 1433 (s), 1320 (s), 1228 (vs), 1198 (s), 1169 (m), 1129 (s), 1117 (vs), 1062 (w), 1047 (s), 1028 (w), 944 (w), 906 (w), 875 (w), 797-698 (s), 768 (vs), 674 (m), 655 (w), 617-569 (vw), 513 (w), 475 (w), 458 (m) (cm^{-1}); Anal. Calcd for $C_{24}H_{20}N_4$, C, 79.10; H, 5.53; N, 15.37%; Found C, 79.08; H, 5.50; N, 15.42%.

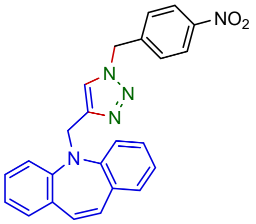

5-((1-(4-Nitrobenzyl)-1*H*-1,2,3-triazol-4-yl)methyl)-5*H*-dibenzo[b,f]azepine (**4e**)

(Yield 87%; 0.356 mg; Cream solid), m.p. 128-130 °C; ^{1}H NMR ($CDCl_3$, 400 MHz): δ 5.06 (s, CH_2), 5.48 (s, CH_2), 6.64 (s, 2H, aromatic ring), (m, 2H, aromatic ring), 6.86 (d, J = 12 Hz, 2H, aromatic ring), 6.92 (t, J = 8 Hz, 2H, aromatic ring), 6.98 (d, J = 8 Hz, 2H, aromatic ring), 7.00 (d, J = 4 Hz, 2H, aromatic ring), 7.16 (m, 3H, aromatic ring), 8.01 (d, J = 12 Hz, 2H, aromatic ring) (ppm); $^{13}C\{^1H\}$ NMR ($CDCl_3$, 101 MHz): δ 142.12, 133.50, 132.06 (2C), 131.50 (2C) , 129.44, 129.18 (2C), 129.09 (4C), 127.60 (2C), 124.12 (2C), 123.82 , 120.48 (2C), 120.27 (2C), 52.81, 47.35 (ppm); FT-IR (KBr): $\bar{\upsilon}$ 3074 (w), 3019 (w), 2926 (m), 2855 (m), 2378 (w), 1926 (vw), 1703 (w), 1604 (m), 1522 (vs), 1485 (s), 1459 (s), 1376 (vw), 1346 (vs), 1294 (m), 1222 (s), 1160 (m), 1116 (s), 1045 (s), 1015-859 (w), 791 (s), 769 (s), 635-592 (vw), 511 (w), 454 (w) (cm^{-1}); Anal. Calcd for $C_{24}H_{19}N_5O_2$, C, 70.40; H, 4.68; N, 17.10%; Found C, 70.38; H, 4.65; N, 17.11%.

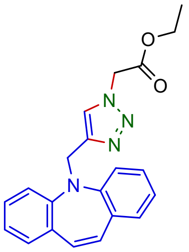

Ethyl 2-(4-((5*H*-dibenzo[b,f]azepin-5-yl)methyl)-1*H*-1,2,3-triazol-1-yl)acetate (**4f**)

(Yield 84%; 0.303 mg; Cream solid), m.p. 78-80 °C; ^{1}H NMR ($CDCl_3$, 400 MHz): δ 1.13 (t, J = 8 Hz, CH_3), 4.09 (q, J = 8 Hz, CH_2), 4.91(s, CH_2), 5.07 (s, CH_2), 6.70 (s, 2H, aromatic ring), 6.91 (t, J = 8 Hz, aromatic ring), 7.01 (m, 4H, aromatic ring), 7.15 (t, J = 8 Hz, aromatic ring), 7.29 (s, 1H, aromatic ring) (ppm); $^{13}C\{^1H\}$ NMR ($CDCl_3$, 101 MHz): δ 166.10, 133.50 (2C), 132.10 (2C), 129.46, 129.11 (2C), 129.06 (4C), 123.80, 123.76 (2C), 120.34 (2C), 62.24, 50.93, 47.29, 14.00 (ppm); FT-IR (KBr): $\bar{\upsilon}$ 3145 (vs), 2963 (w), 2987 (w), 2926 (w), 2854 (w), 2378 (vw), 1794 (vs), 1759 (m), 1612 (w), 1460 (m), 1400 (vw), 1376 (w), 1340 (w), 1284 (vw), 1214 (vs), 11130-1031 (m), 940-877 (vw), 794 (s), 766 (s), 750 (m), 721 (vw), 511 (vw), 451 (vw) (cm^{-1}); Anal. Calcd for $C_{21}H_{20}N_4O_2$, C, 69.98; H, 5.59; N, 15.55%; Found C, 70.00; H, 5.56; N, 15.53%.

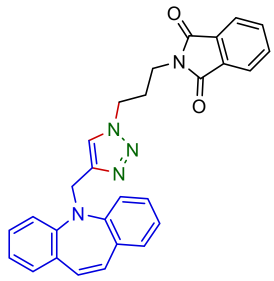

2-(3-(4-((5*H*-Dibenzo[b,f]azepin-5-yl)methyl)-1*H*-1,2,3-triazol-1-yl)propyl)isoindoline-1,3-dione (**4g**)

(Yield 82%; 0.378 mg; Cream solid), m.p. 141-142 °C; 1H NMR ($CDCl_3$, 400 MHz): δ 1.18 (t, *J* = 12 Hz, CH_3), 2.09 (t, *J* = 6 Hz, CH_2), 3.52 (t, *J* = 8 Hz, CH_2), 4.19 (t, J = 6 Hz, CH_2), 5.01 (s, CH_2), 6.92 (t, *J* = 6 Hz, 2H, aromatic ring), 7.05 (m, 4H, aromatic ring), 7.15 (m, 2H, aromatic ring), 7.24 (t, *J* = 8 Hz, 2H, aromatic ring), 7.65-7.67 (s, 1H, aromatic ring and d.d, *J* = 8 Hz, *j* = 4 Hz, 2H, aromatic ring), 7.78 (d, *J* = 4 Hz, aromatic ring) (ppm); $^{13}C\{^1H\}$ NMR ($CDCl_3$, 101 MHz): δ 168.19 (2C), 134.17 (2C), 133.35 (2C), 132.04 (2C), 131.89 (2C), 129.58, 129.23 (2C), 129.12 (4C), 123.98 (2C), 123.53, 123.40 (2C), 120.66 (2C), 47.90, 47.36, 34.88, 29.41 (ppm); FT-IR (KBr): $\bar{\upsilon}$ 3444 (s), 2949 (m), 2926 (m), 2856 (w), 2378 (w), 1765 (m), 1711 (vs), 1464 (w), 1396 (m), 1143 (s), 1048 (w), 940 (m), 894 (m), 852 (w), 812 (vw), 792 (w), 770 (m), 720 (s), 613 (w), 513 (s) (cm^{-1}); Anal. Calcd for $C_{28}H_{23}N_5O_2$, C, 72.87; H, 5.02; N, 15.17%; Found C, 72.84; H, 4.99; N, 15.20%.

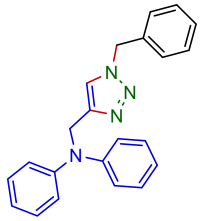

N-((1-Benzyl-1*H*-1,2,3-triazol-4-yl)methyl)-*N*-phenylaniline (**4h**)

(Yield 96%; 0.327 mg; White solid), m.p. 104-105 °C; 1H NMR ($CDCl_3$, 400 MHz): δ 4.99 (s, CH_2), 5.36 (s, CH_2), 6.86 (t, *J* = 8 Hz, 2H, aromatic ring), 6.96 (d, *J* = 8 HZ, 4H, aromatic ring), 7.05 (d.d, *J* = 8 HZ, *J* = 4 HZ, 2H, aromatic ring), 7.15 (t, *J* = 8 HZ, 5H, aromatic ring), 7.24 (d, *J* = 8 Hz, 2H, aromatic ring and s, 1H, aromatic ring) (ppm); $^{13}C\{^1H\}$ NMR ($CDCl_3$, 101 MHz): δ 147.49 (2C), 134.77, 129.34 (5C), 129.03 (2C), 128.59 (2C), 127.68, 121.73 (3C), 120.93 (4C), 54.06, 48.57 (ppm); FT-IR (KBr): $\bar{\upsilon}$ 3113 (w), 3066 (m), (m), 3036 (m), 2956 (w), 2854 (w), 2375 (vw), 1937 (vw), 1590 (vs), 1575 (m), 1497 (vs), 1494 (vs), 1456 (m), 1440 (w), 1368 (s), 1338-1276 (w), 1257 (s), 1226 (s), 1214 (m),1184-990 (w), 862 (m), 828 (w), 780 (w), 748 (vs), 736 (m), 701 (vs), 693 (vs), 620 (vw), 606 (w), 589 (vw), 508 (w), 497 (w), 464 (vw) (cm^{-1}); Anal. Calcd for $C_{22}H_{20}N_4$, C, 77.62; H, 5.92; N, 16.46%; Found C, 77.60; H, 5.98; N, 16.51%.

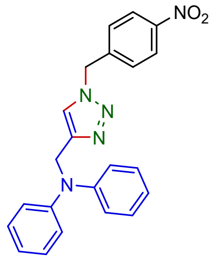

N-((1-(4-nitrobenzyl)-1*H*-1,2,3-triazol-4-yl)methyl)-*N*-phenylaniline (**4i**)

(Yield 95%; 0.366 mg; Yellow solid), m.p. 90-92 °C; 1H NMR ($CDCl_3$, 400 MHz): δ 5.03 (s, CH_2), 5.48 (s,CH_2), 6.88 (t, J = 8 Hz, 2H, aromatic ring), 6.98 (d, J = 8 Hz, 4H, aromatic ring), 7.17 (m, 7H, aromatic ring), 8.10 (d, J = 12 Hz, 2H, aromatic ring) (ppm); $^{13}C\{^1H\}$ NMR ($CDCl_3$, 101 MHz): δ 147.36 (2C), 146.92, 141.79, 129.38 (5C), 128.21 (2C), 124.24 (2C), 122.34, 121.87 (2C), 120.88 (4C), 53.01, 48.52 (ppm); FT-IR (KBr): $\bar{\upsilon}$ 3442 (vw), 3134 (m), 3082 (w), 2996 (vw), 2940 (s), 2932 (s), 2859 (m), 2446 (vw), 1648 (vs), 1608 (m), 1518 (s), 1487 (s), 1458 (m), 1426 (m), 1348 (vs), 1300-1230 (w), 1194 (s), 1125 (m), 1109 (m), 1086 (w), 1053 (w), 974 (w), 956 (w), 929 (vw), 858-816 (w), 804 (m), 792 (m), 734 (m), 717 (m), 632 (vw), 572 (vw), 521 (m), 488 (w), 409 (vw) (cm^{-1}); Anal. Calcd for $C_{22}H_{19}N_5O_2$, C, 68.56; H, 4.97; N, 18.17%; Found C, 68.60; H, 4.93; N, 18.14%.

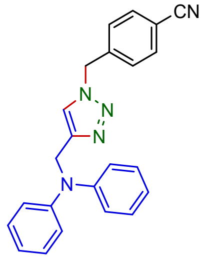

4-((4-((Diphenylamino)methyl)-1*H*-1,2,3-triazol-1-yl)methyl)benzonitrile (**4j**)

(Yield: 93%; 0.340 mg; Cream solid), 88-90 °C; ^1H NMR ($CDCl_3$, 400 MHz): δ 5.11 (s, CH_2), 5.52 (s, CH_2), 6.98 (t, J = 6 Hz, 2H, aromatic ring), 7.07 (d, J = 8 Hz, 4H, aromatic ring), 7.20 (d, J = 8 Hz, 2H, aromatic ring), 7.26 (t, J = 8 Hz, 4H, aromatic ring), 7.30 (s, 1H, aromatic ring), 7.62 (d, J = 8 Hz, 2H, aromatic ring) (ppm); $^{13}C\{^1H\}$ NMR ($CDCl_3$, 101 MHz): δ 147.41, 146.83, 140.03, 132.80 (2), 129.38, 128.03 (6), 122.33, 121.82 (2), 120.88 (4), 118.20, 112.57, 53.23, 48.48 (ppm); FT-IR (KBr): $\bar{\upsilon}$ 3441 (w), 3135 (vw), 3059 (w), 2925 (w), 2853 (w), 2429 (vw), 2230 (m), 1938 (vw), 1590 (s), 1534 (vw), 1522 (w), 1496 (vs), 1458 (m), 1417 (w), 1361 (s), 1340 (m), 1272 (w), 1249 (m), 1223 (s), 1156 (w), 1128 (m), 1094 (w), 1048 (s), 1021 (w), 990 (vw), 855 (w), 817 (w), 783 (m), 752 (s), 696 (s), 549 (vw), 605 (vw), 588 (vw), 549 (m), 615 (m) (cm^{-1}); Anal. Calcd for $C_{23}H_{19}N_5$, C, 75.59; H, 5.24; N, 19.16%; Found C, 75.57; H, 5.22; N, 19.20%.

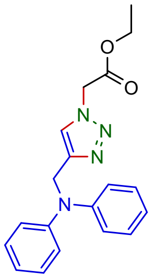

Ethyl 2-(4-((diphenylamino)methyl)-1*H*-1,2,3-triazol-1-yl)acetate (**4k**)

(Yield 92%; 0.309 mg; Cream solid), m.p. 123-124 °C; 1H NMR ($CDCl_3$, 400 MHz): δ 1.17 (t, *J* = 6 Hz, CH_3), 4.14 (q, *J* = 6 Hz, CH2), 4.99 (s,CH_2), 5.04 (s,CH_2), 6.89 (t, *J* = 6 Hz, 2H, aromatic ring), 7.02 (d, *J* = 8 Hz, 4H, aromatic ring), 7.18 (t, *J* = 8 Hz, 2H, aromatic ring), 7.37 (s, 1H, aromatic ring) (ppm); $^{13}C\{^1H\}$ NMR ($CDCl_3$, 101 MHz): δ 166.15, 147.38 (2C), 129.38 (5C), 123.41, 121.82 (2C), 120.86 (4C), 62.37, 50.92, 48.54, 14.02 (ppm); FT-IR (KBr): $\bar{\upsilon}$ 3132 (w), 3080 (w), 2993 (m), 2957 (w), 2934 (w), 2858 (vw), 2373 (vw), 1753 (vs), 1602 (m), 1590 (s), 1573 (m), 1500 (vs), 1458-1394 (w), 1368 (s), 1334 (w), 1277 (w), 1254 (s), 1228 (vs), 1212 (vs), 1180 (m), 1137-1028 (w), 875 (vw), 856 (w), 791 (w), 780 (vw), 750 (s), 733 (w), 693 (s), 574 (vw), 602 (w), 576 (vw), 512 (w), 494 (m), 416 (m) (cm^{-1}); Anal. Calcd for $C_{19}H_{20}N_4O_2$, C, 67.84; H, 5.99; N, 16.66%; Found C, 67.81; H, 5.95; N, 16.69%.

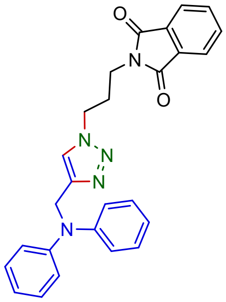

2-(3-(4-((Diphenylamino)methyl)-1*H*-1,2,3-triazol-1-yl)propyl)isoindoline-1,3-dione (**4l**)

(Yield 90%; 0.394 mg; Pale yellow oil); ^1H NMR ($CDCl_3$, 400 MHz): δ 2.27 (p, *J* = 4 Hz, CH_2), 3.71 (t, *J* = 6 Hz, CH_2), 4.34 (t, J = 6 Hz, CH_2), 5.08 (s, CH_2), 6.97 (t, *J* = 6 Hz, 2H, aromatic ring), 7.11 (d, *J* = 8 Hz, 4H, aromatic ring), 7.27 (t, *J* = 8 Hz, 4H, aromatic ring), 7.48 (s, 1H, aromatic ring), 7.74 (d.d, *J* = 8 Hz, *j* = 4 Hz, 2H, aromatic ring), 7.85 (d, *J* = 4 Hz, 2H, aromatic ring) (ppm); $^{13}C\{^1H\}$ NMR ($CDCl_3$, 101 MHz): δ 168.24 (2C), 147.50 (2C), 134.21 (4C), 131.88, 129.37 (4C), 123.40 (2C), 122.41, 121.68 (2C), 120.88 (4C), 48.53, 47.88, 35.00, 29.40 (ppm); FT-IR (KBr): $\bar{\upsilon}$ 3464 (w), 3130 (w), 3062 (w), 2933 (w), 1773 (m), 1717 (vs), 1590 (m), 1500 (s), 1466 (m) 1437 (m), 1401 (s), 1368 (s), 1341 (w), 1299 (vw), 1255-1032 (m), 989 (vw), 890 (w), 856 (w), 780 (w), 746 (s), 714 (s), 693 (s), 602 (w), 530 (w) 512 (w) (cm^{-1}); Anal. Calcd for $C_{26}H_{23}N_5O_2$, C, 71.38; H, 5.30; N, 16.01%; Found C, 71.34; H, 5.26; N, 16.05%.

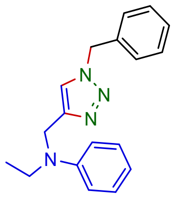

N-((1-Benzyl-1*H*-1,2,3-triazol-4-yl)methyl)-*N*-ethylaniline (**4m**)

(Yield 95%; 0.278 mg; Cream solid), 104-105 °C; 1H NMR ($CDCl_3$, 400 MHz): δ 1.09 (t, J = 6 Hz, CH_3), 3.35 (q, *J* = 8 Hz, CH_2), 4.52 (s, CH_2), 5.38 (s, CH_2), 6.63 (m, 3H, aromatic ring), 7.20 (m, 8H, aromatic ring) (ppm); $^{13}C\{^1H\}$ NMR ($CDCl_3$, 101 MHz): δ 146.96, 134.79, 129.35, 129.05 (2C), 128.63 (2C), 127.79 (2C), 127.67, 121.66, 116.57, 112.58 (2C), 54.09, 46.34, 45.26, 12.20 (ppm); FT-IR (KBr): $\bar{\upsilon}$ 3444 (w), 3121 (w), 3075 (w), 2980 (m), 2930 (w), 2892 (w), 1597 (vs), 1513 (vs) 1506 (vs), 1376 (m), 1382 (m), 1348 (s), 1241 (m), 1220 (m), 1189 (m), 1132 (m), 1112 (m), 1054 (w), 975 (vw), 889 (vw), 802 (w), 789 (w), 745 (s), 736 (m), 514 (m), 469 (vw) (cm^{-1}); Anal. Calcd for $C_{18}H_{20}N_4$, C, 73.94; H, 6.89; N, 19.16%; Found C, 73.90; H, 6.86; N, 19.23%.

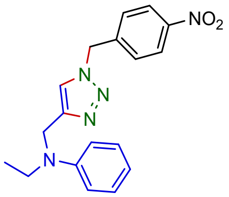

N-ethyl-*N*-((1-(4-nitrobenzyl)-1*H*-1,2,3-triazol-4-yl)methyl)aniline (**4n**)

(Yield: 92%; 0.310 mg; Yellow solid), m.p. 102-104 °C; 1H NMR ($CDCl_3$, 400 MHz): δ 1.20 (t, *J* = 8 Hz, CH_3), 3.46 (q, *J* = 6 Hz, CH_2), 4.65 (s, CH_2), 5.59 (s, CH_2), 6.73 (d, *J* = 8 Hz, 3H, aromatic ring), 7.22 (t, *J* = 8 Hz, 2H, aromatic ring), 7.34 (d, *J* = 8 Hz, 2H, aromatic ring and s, 1H, aromatic ring), 8.21 (d, *J* = 8 Hz, 2H, aromatic ring) (ppm); $^{13}C\{^1H\}$ NMR ($CDCl_3$, 101 MHz): δ 148.01, 147.66, 141.83, 129.37 (3C), 128.37 (2C), 124.26 (2C), 121.96, 116.76, 112.66 (2C), 53.03, 46.32, 45.45, 12.32 (ppm); FT-IR (KBr): $\bar{\upsilon}$ 3121 (w), 3075 (w), 2978 (m), 2983 (w), 2891 (w), 2425, 1638 (vw), 1598 (vs), 1572 (w), 1555 (vw), 1507 (vw), 1452 (vw), 1436 (vw), 1395 (w), 1376 (m), 1382 (m), 1350 (vs), 1332 (m), 1274 (w), 1241-1112 (m), 1076-1054 (w), 976 (w), 890 (vw), 830-717 (w), 745 (s), 736 (m), 717 (w), 693 (w), 512 (m), 468 (vw) (cm^{-1}); Anal. Calcd for $C_{18}H_{19}N_5O_2$, C, 64.08; H, 5.68; N, 20.76%; Found C, 64.04; H, 5.63; N, 20.80%.

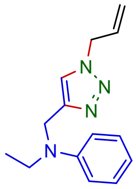

N-((1-allyl-1*H*-1,2,3-triazol-4-yl)methyl)-*N*-ethylaniline (**4o**)

(Yield: 87%; 0.211 mg; Cream oil); 1H NMR ($CDCl_3$, 400 MHz): δ 1.20 (t, J = 6 Hz, CH_3), 3.47 (q, J = 8 Hz, CH_2), 4.65 (s, CH_2), 4.89 (d, J = 8 Hz, CH_2), 5.24 (d, J = 16 Hz, CH), 5.30 (d, J = 12 Hz, CH), 5.96 (m, CH), 6.75 (m , 3H, aromatic ring), 7.23 (t, J = 8 Hz, 2H, aromatic ring), 7.37 (s, 1H, aromatic ring) (ppm); $^{13}C\{^1H\}$ NMR ($CDCl_3$, 101 MHz): δ 146.53, 132.53, 131.34, 129.37 (2C), 129.31, 121.71, 119.95, 118.48, 117.29 (2C), 52.67, 46.39, 45.39, 12.31 (ppm); FT-IR (KBr): $\bar{\upsilon}$ 3446 (vw), 3136-3026 (vw), 2972 (m), 2930 (w), 2870 (w), 2412 (vw), 1599 (vs), 1574 (w), 1558 (vw), 1506 (vw), 1461 (w), 1418 (vw), 1375 (m), 1353 (m), 1274 (w), 1236 (w), 1218 (m), 1218 (m), 1188 (m), 1130 (w), 1074 (vw), 1048 (m), 989 (m), 935 (w), 886 (vw), 795 (m), 749 (vs), 694 (s), 557 (vw), 514 (w) (cm^{-1}); Anal. Calcd for $C_{14}H_{18}N_4$, C, 69.39; H, 7.49; N, 23.12%; Found C, 69.35; H, 7.47; N, 23.19%.

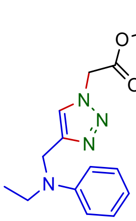

Ethyl 2-(4-((ethyl(phenyl)amino)methyl)-1*H*-1,2,3-triazol-1-yl)acetate (**4p**)

(Yield 91%; 0.262 mg; Colorless oil); 1H NMR ($CDCl_3$, 400 MHz): δ 1.21 (t, J = 6 Hz, CH_3), 1.27 (t, J = 8 Hz, CH_3), 3.48 (q, J = 8 Hz, CH_2); 4.23 (q, J = 8 Hz, CH_2), 4.65 (s, CH_2), 5.07 (s, CH_2), 6.74 (m, 3H, aromatic ring), (t, J = 8 Hz, 2H, aromatic ring), 7.45 (s, 1H, aromatic ring) (ppm); $^{13}C\{^1H\}$ NMR ($CDCl_3$, 101 MHz): δ 166.30, 146.78, 129.37 (3C), 123.31, 116.63, 112.66 (2C), 62.33, 50.83, 46.32, 45.39, 14.04, 12.32 (ppm); FT-IR (KBr): $\bar{\upsilon}$ 3471 (vw), 3139 (vw), 2977 (m), 2937 (w), 2428 (vw), 1756 (vs), 1599 (vs), 1574 (w), 1558 (vw), 1507 (vs), 1463 (w), 1395 (m), 1376 (s), 1353 (s), 1273 (s), 1216 (vs), 1132 (w), 1074 (vw), 1050 (s), 1027 (s), 988 (vw), 876 (w), 795 (w), 750 (vs), 695 (s), 576 (vw), 514 (w) (cm^{-1}); Anal. Calcd for $C_{15}H_{20}N_4O_2$, C, 62.48; H, 6.99; N, 19.43%; Found C, 62.46; H, 6.96; N, 19.45%.

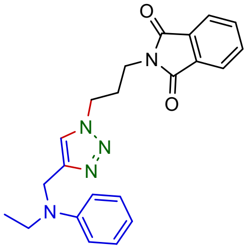

2-(3-(4-((Ethyl(phenyl)amino)methyl)-1*H*-1,2,3-triazol-1-yl)propyl)isoindoline-1,3-dione (**4q**)

(Yield 89%; 0.347 mg; Cream solid), m.p. 90-91 °C; ^1H NMR ($CDCl_3$, 400 MHz): δ 1.21 (t, J = 8 Hz, CH_3), 2.29 (p, J = 8 Hz, CH_2), 3.48 (q, J = 8 Hz, CH_2), 3.73 (t, J = 6 Hz, CH_2), 4.35 (t, J = 8 Hz, CH_2), 4.61 (s, CH_2), 6.71 (t, J = 4 Hz, 1H, aromatic ring), 6.77 (d, J = 8 Hz, 2H, aromatic ring), 7.22 (t, J = 8 Hz, 2H, aromatic ring), 7.45 (s, 1H, aromatic ring), 7.74 (d.d, J = 8 Hz, j = 4

Hz, 2H, aromatic ring), 7.85 (d, $J = 4$ Hz, 2H, aromatic ring) (ppm); $^{13}C\{^1H\}$ NMR ($CDCl_3$, 101 MHz): δ 168.25 (2C), 147.80, 134.21 (4), 131.88, 129.34 (2C), 123.40 (2C), 122.15, 116.53, 112.58 (2C), 47.85, 46.23, 45.28, 35.02, 29.41, 12.34 (ppm); FT-IR (KBr): $\bar{\upsilon}$ 3455 (w), 3140 (vw), 3060 (vw), 2969 (w), 2932 (w), 2870 (vw), 1773 (m), 1714 (vs), 1599 (s), 1508 (s), 1468 (w), 1438 (w), 1398 (s), 1376 (s), 1273 (vw), 1215 (w), 1189 (m), 1143 (w), 1049 (w), 1037 (w), 987 (vw), 889 (w), 795 (w), 750 (m), 721 (s), 694 (m), 531 (m) (cm^{-1}); Anal. Calcd for $C_{22}H_{23}N_5O_2$, C, 67.85; H, 5.95; N, 17.98%; Found C, 67.83; H, 5.92; N, 18.00%.

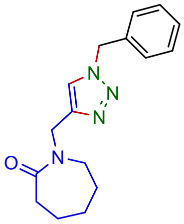

1-((1-Benzyl-1*H*-1,2,3-triazol-4-yl)methyl)azepan-2-one (**4r**)

(Yield 90%; 0.256 mg; White solid), m.p. 97-98 °C; 1H NMR ($CDCl_3$, 400 MHz): δ 1.52 (p, $J = 6$ Hz, CH_2), 1.65 (m, 4H, CH_2), 2.49 (t, $J = 6$ Hz, CH_2), 3.48 (t, $J = 4$ Hz, CH_2), 4.59 (s, CH_2), 5.48 (s, CH_2), 7.24 (m, 2H, aromatic ring), 7.36 (m, 3H, aromatic ring) (ppm); $^{13}C\{^1H\}$ NMR ($CDCl_3$, 101 MHz): δ 175.81, 145.12, 134.59, 129.08 (2C), 128.72 (2C), 128.06, 122.74, 54.16, 49.78, 43.18, 37.01, 29.84, 28.16, 23.26 (ppm); FT-IR (KBr): $\bar{\upsilon}$ 3414 (m), 3243 (w), 3209 (w), 3131 (vw), 3076 (vw), 2972 (vw), 2934 (m), 2856 (w), 2408 (vw), 1630 (vs), 1621 (vs), 1494-1464 (m), 1370-1050 (w), 977-812 (vw), 763-701 (w), 666 (vw), 577 (vw), 515 (w), 474 (vw) (cm^{-1}); Anal. Calcd for $C_{16}H_{20}N_4O$, C, 67.58; H, 7.09; N, 19.70%; Found C, 67.55; H, 7.06; N, 19.72%.

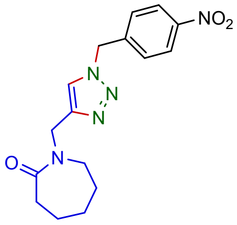

1-((1-(4-Nitrobenzyl)-1*H*-1,2,3-triazol-4-yl)methyl)azepan-2-one (**4s**)

(Yield 93%; 0.306 mg; White solid); ^1H NMR ($CDCl_3$, 400 MHz): δ 1.58 (m, 2H, CH_2), 1.64 (m, 2H, CH_2), 1.69 (m, 2H, CH_2), 2.51 (t, $J = 6$ Hz, CH_2), 3.53 (t, $J = 6$ Hz, CH_2), 4.62 (s, CH_2), 5.62 (s, CH_2), 7.41 (d, $J = 8$ Hz, aromatic ring), 7.61 (s, aromatic ring), 8.24 (d, $J = 8$ Hz, aromatic ring) (ppm); $^{13}C\{^1H\}$ NMR ($CDCl_3$, 101 MHz): δ 175.96, 148.08, 145.71, 141.63, 128.62 (2C), 124.31 (2C), 123.19, 53.10, 50.16, 43.49, 37.02, 29.85, 28.26, 23.28 (ppm); FT-IR (KBr): $\bar{\upsilon}$ 3442 (w), 3133 (m), 3082 (w), 2996 (w), 2940 (s), 2932 (s), 2859 (m), 2446 (vw), 1648 (vs), 1608 (m), 1518 (s), 1487 (s), 1458 (m), 1426 (m), 1348 (vs), 1230 (m), 1259 (w), 1230 (m), 1194 (s), 1125 (m), 1109 (m), 1086-956 (w), 929 (vw), 858 (w), 841 (w), 816 (w), 804 (m), 792 (m), 766 (w),

734 (m), 717 (m), 686 (vw), 632 (vw), 572 (vw), 521 (m), 488 (w), 409 (vw) (cm^{-1}); Anal. Calcd for $C_{16}H_{19}N_5O_3$, C, 58.35; H, 5.81; N, 21.26%; Found C, 58.32; H, 5.77; N, 21.29%.

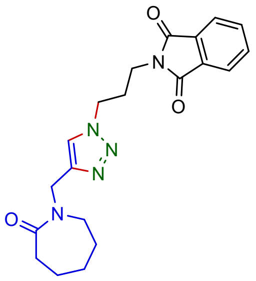

2-(3-(4-((2-Oxoazepan-1-yl)methyl)-1*H*-1,2,3-triazol-1-yl)propyl)isoindoline-1,3-dione (**4t**)

(Yield 88%; 0.336 mg; Pale yellow oil); ^{1}H NMR ($CDCl_3$, 400 MHz): δ 1.46 (m, 2H, CH_2), 1.57 (m, 4H, CH_2), 2.23 (t, J = 4 Hz, CH_2), 2.43 (q, J = 8 Hz, CH_2), 3.41 (t, J = 8 Hz, CH_2), 3.65 (t, J = 8 Hz, CH_2), 4.31 (t, J = 8 Hz, CH_2), 4.51 (s, CH_2), 7.64 (s, 1H, aromatic ring), 7.65 (d, J = 8 Hz, 2H, aromatic ring), 7.74 (t, J = 4 Hz, 2H, aromatic ring) (ppm); $^{13}C\{^{1}H\}$ NMR ($CDCl_3$, 101 MHz): δ δ 175.82, 168.16 (2C), 144.51 (2C), 134.16 (2C), 131.77, 123.30 (2C), 123.18, 49.68, 47.75, 43.07, 36.94, 34.93, 29.79, 29.26, 28.13, 23.19 (ppm); FT-IR (KBr): $\bar{\upsilon}$ 3138 (w), 2933 (s), 2857 (m), 1771 (s), 1714 (vs), 1636 (vs), 1489 (m), 1438 (s), 1399 (s), 1370 (s), 1262 (w), 1196 (m), 1144 (m), 1113 (w), 1087 (w), 1034 (m), 977 (w), 890 (w), 793 (vw), 722 (s), 603 (vw), 576 (vw), 531 (m) (cm^{-1}); Anal. Calcd for $C_{20}H_{23}N_5O_3$, C, 62.98; H, 6.08; N, 18.36%; Found C, 62.94; H, 6.04; N, 18.39%.

Spectral of products

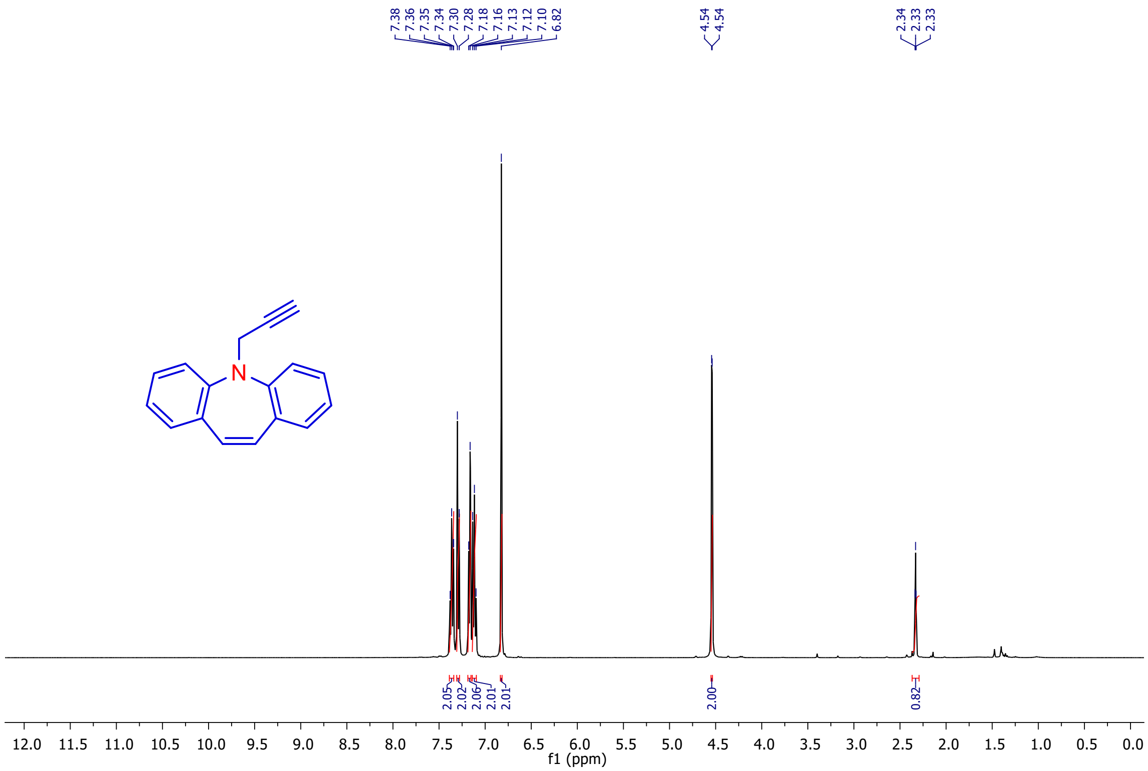

Fig

ure S1. 1H NMR spectrum of (**3b**) in $CDCl_3$ (400 MHz).

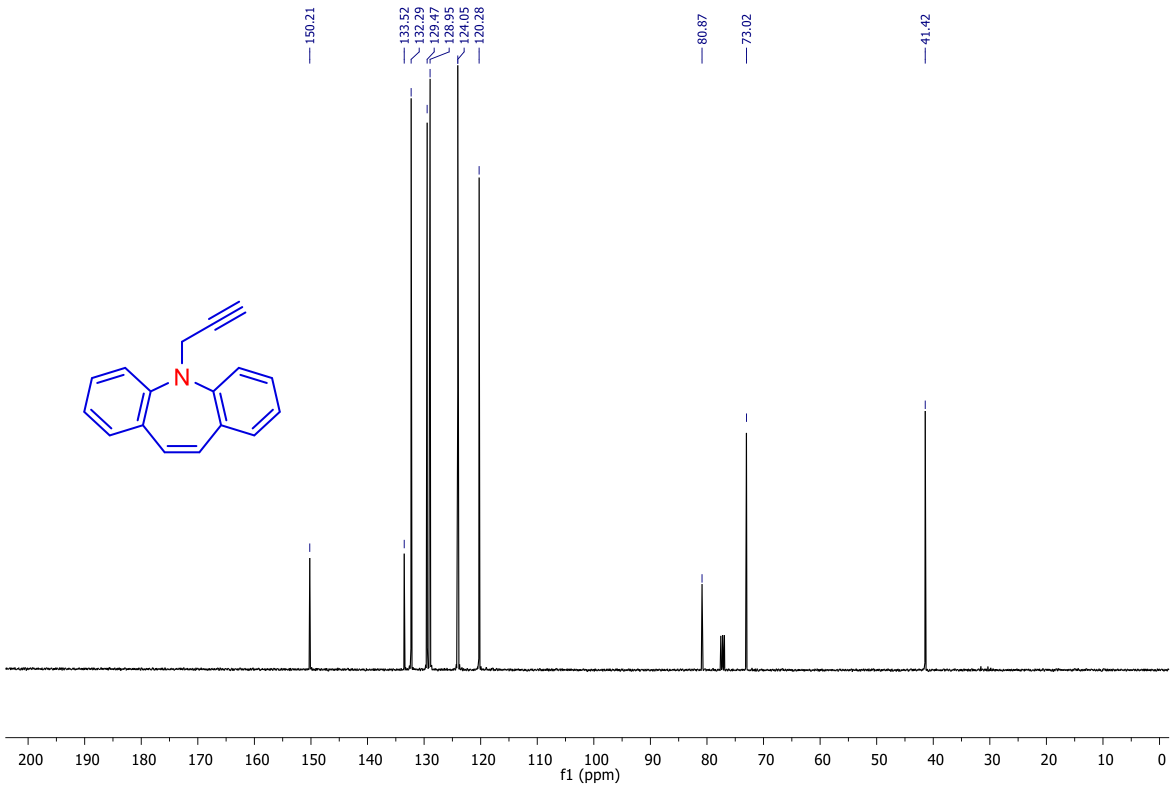

Fi

gure S2. $^{13}C\{^{1}H\}$ NMR spectrum of (**3b**) in $CDCl_3$ (101 MHz).

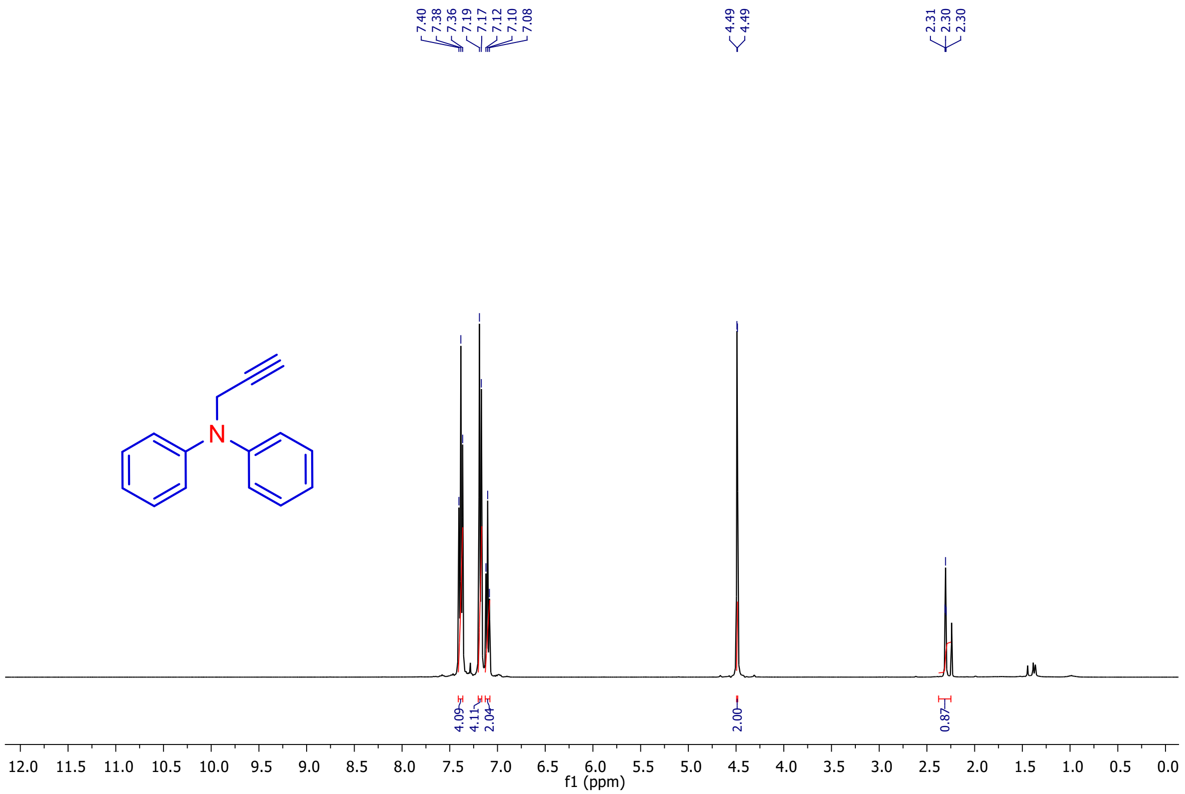

F

igure S3. ^{1}H NMR spectrum of (**3c**) in $CDCl_3$ (400 MHz).

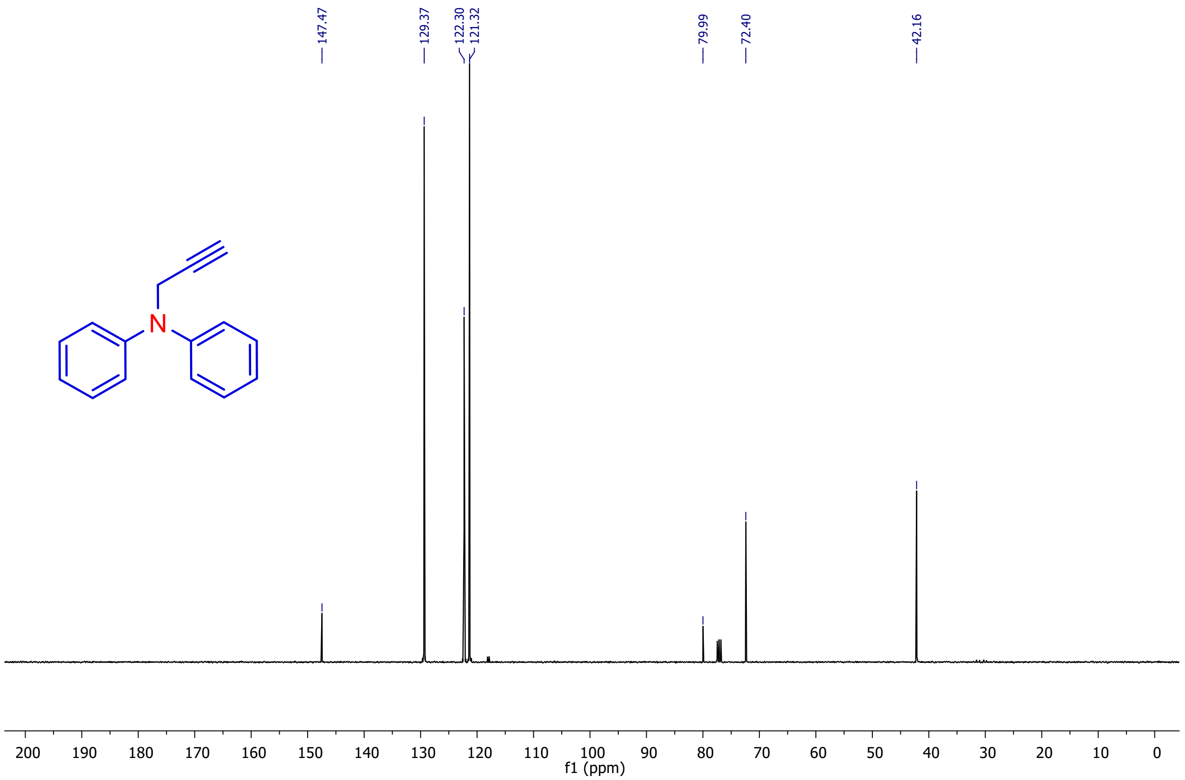

F

igure S4. $^{13}C\{^{1}H\}$ NMR spectrum of (**3c**) in $CDCl_3$ (101 MHz).

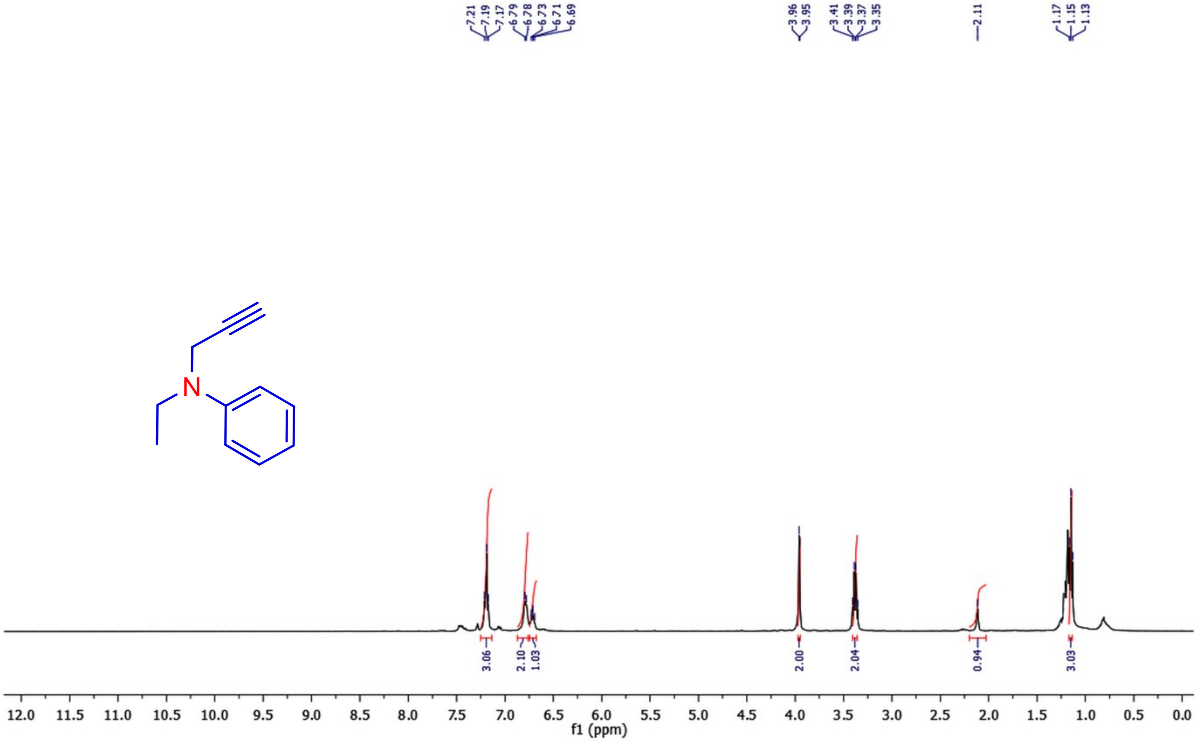

Figure S5. ^{1}H NMR spectrum of (**3d**) in $CDCl_3$ (400 MHz).

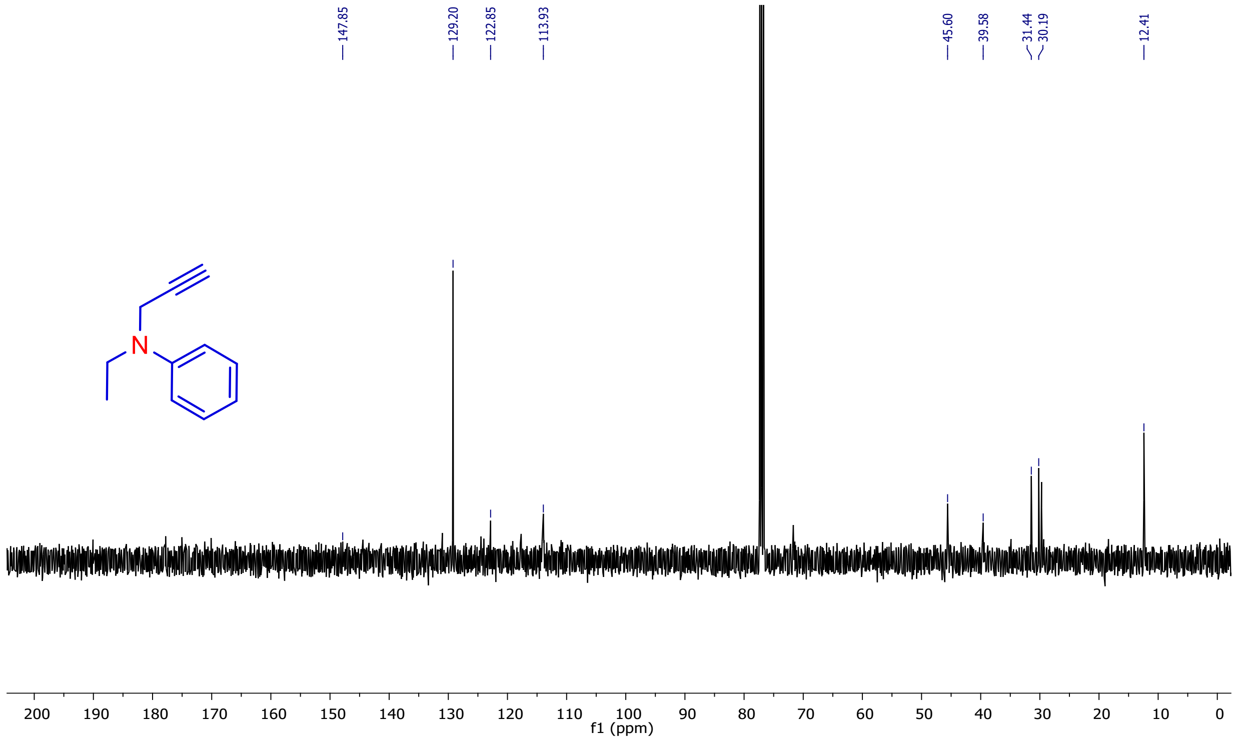

Figure S6. $^{13}C\{^1H\}$ NMR spectrum of (**3d**) in $CDCl_3$ (101 MHz).

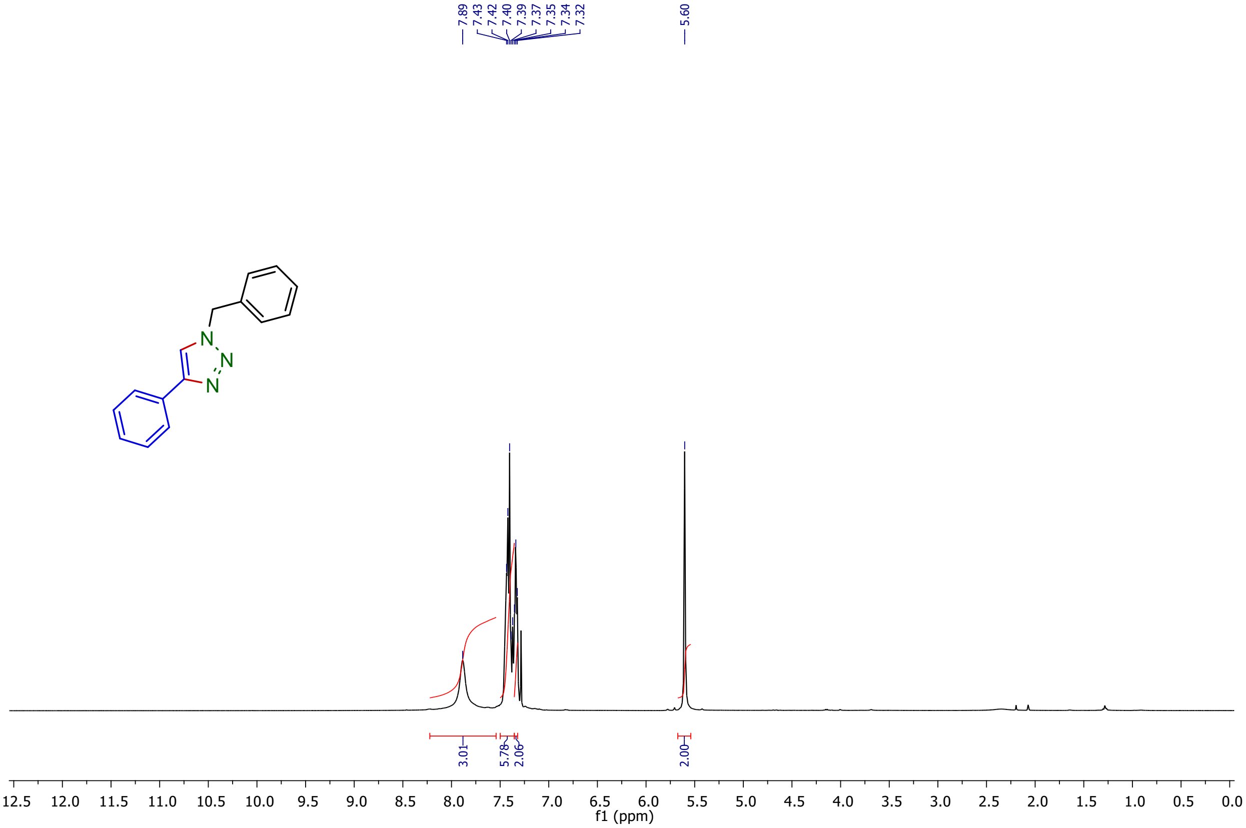

Figure S7. ^1H NMR spectrum of (**4a**) in $CDCl_3$ (400 MHz).

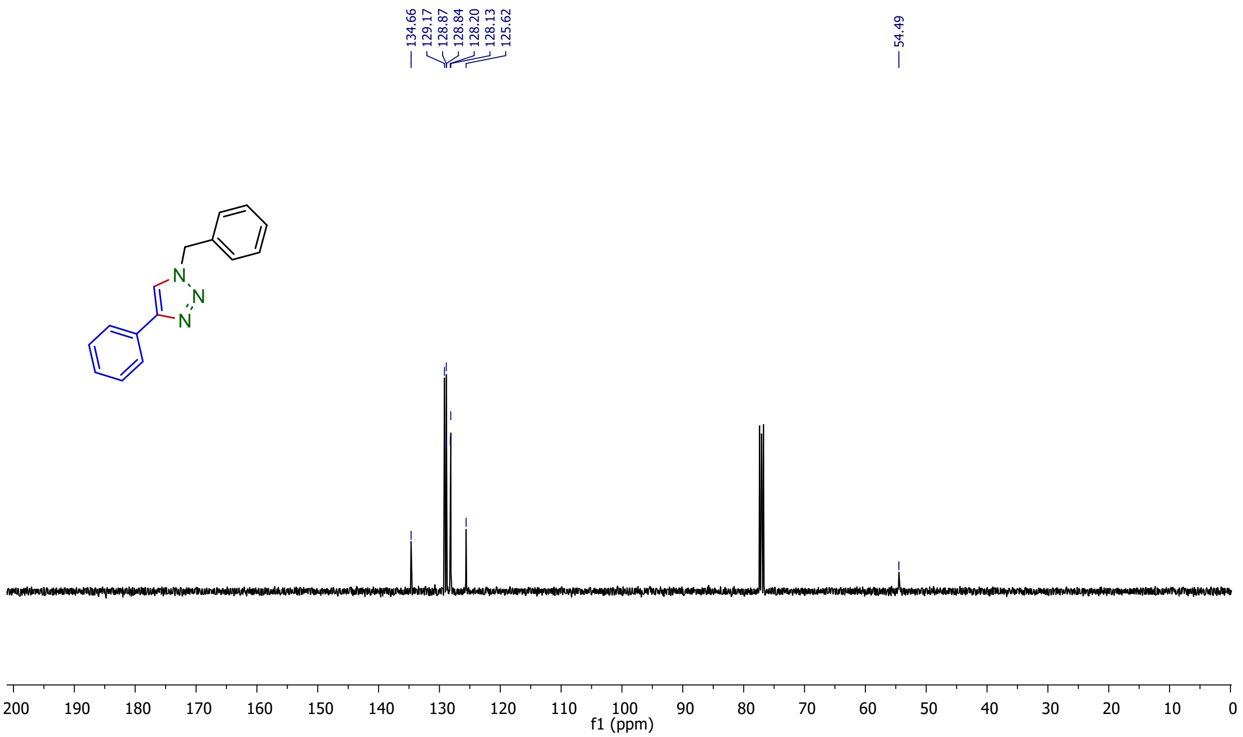

Figure S8. $^{13}C\{^{1}H\}$ NMR spectrum of (**4a**) in $CDCl_3$ (101 MHz).

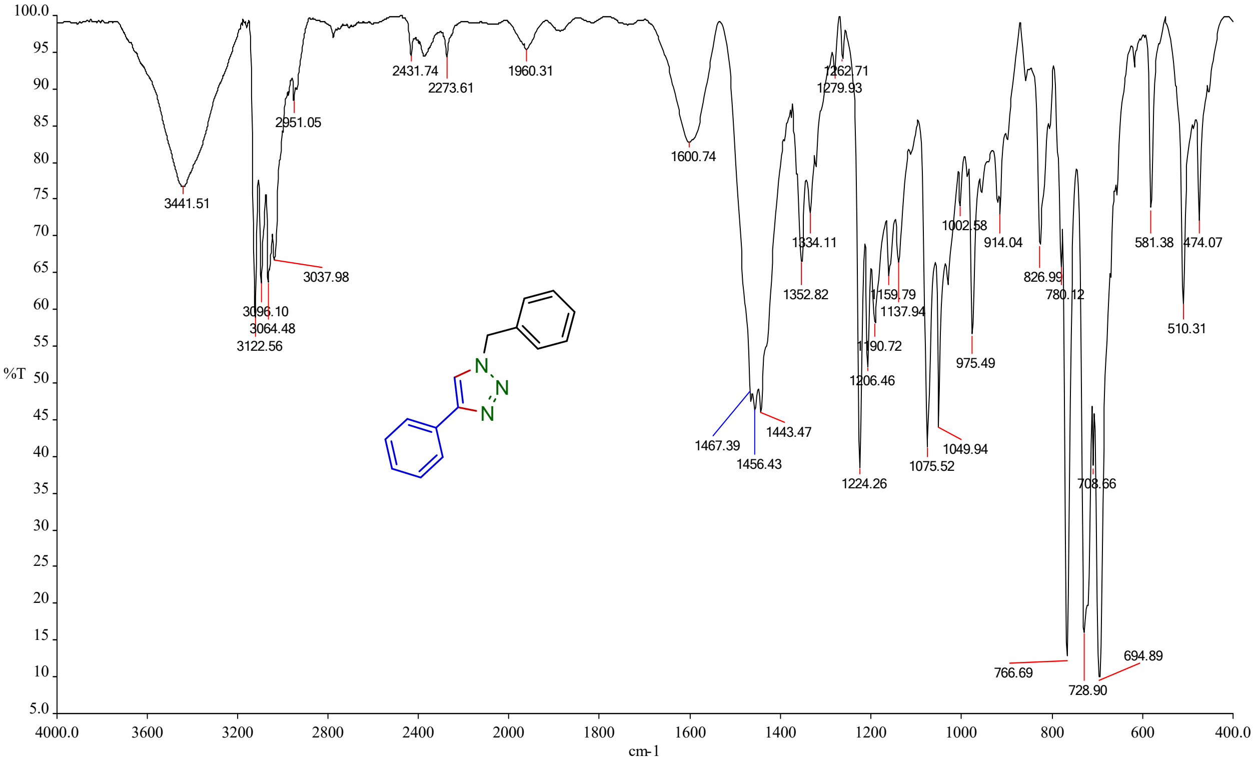

Figure S9. FT-IR spectrum of (**4a**) in KBr

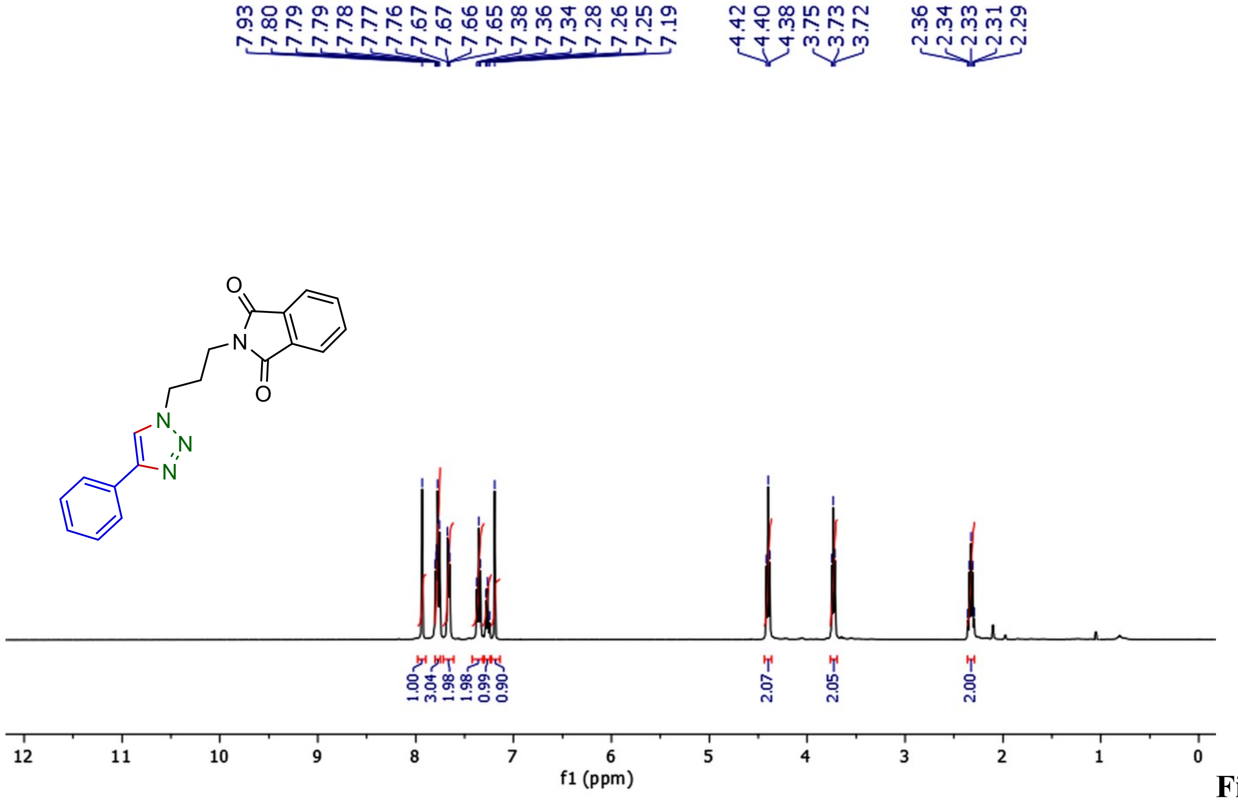

Figure S10. ^{1}H NMR spectrum of (**4b**) in $CDCl_3$ (400 MHz).

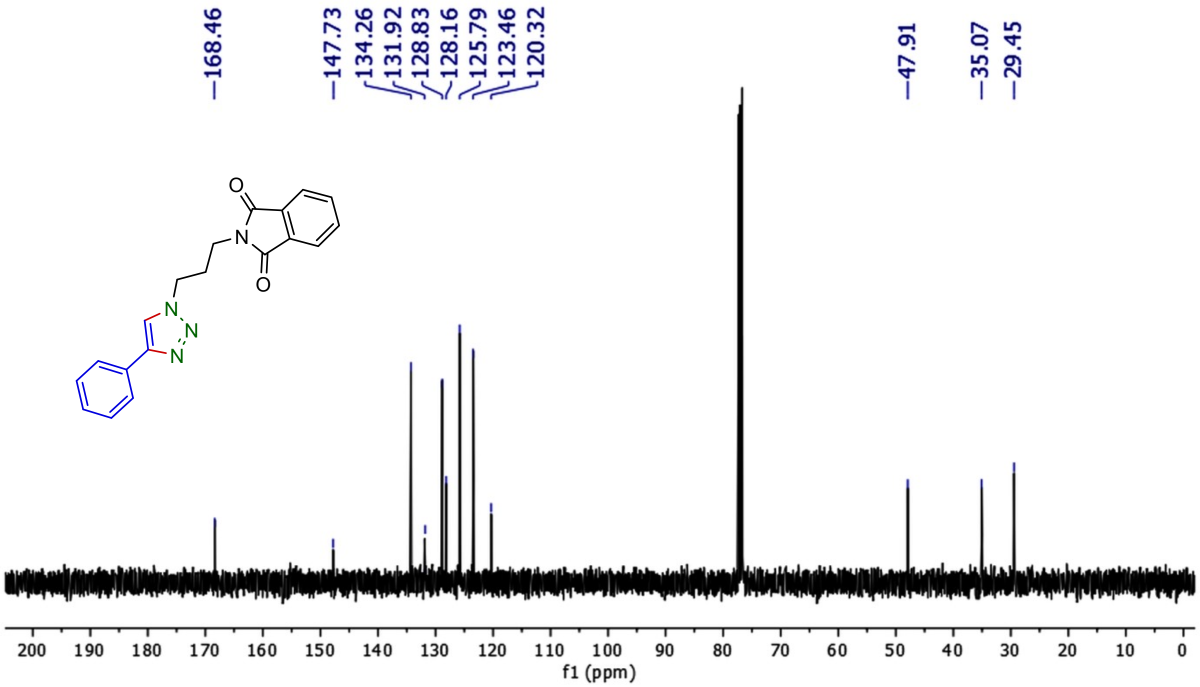

Figure S11. $^{13}C\{^{1}H\}$ NMR spectrum of (**4b**) in $CDCl_3$ (101 MHz).

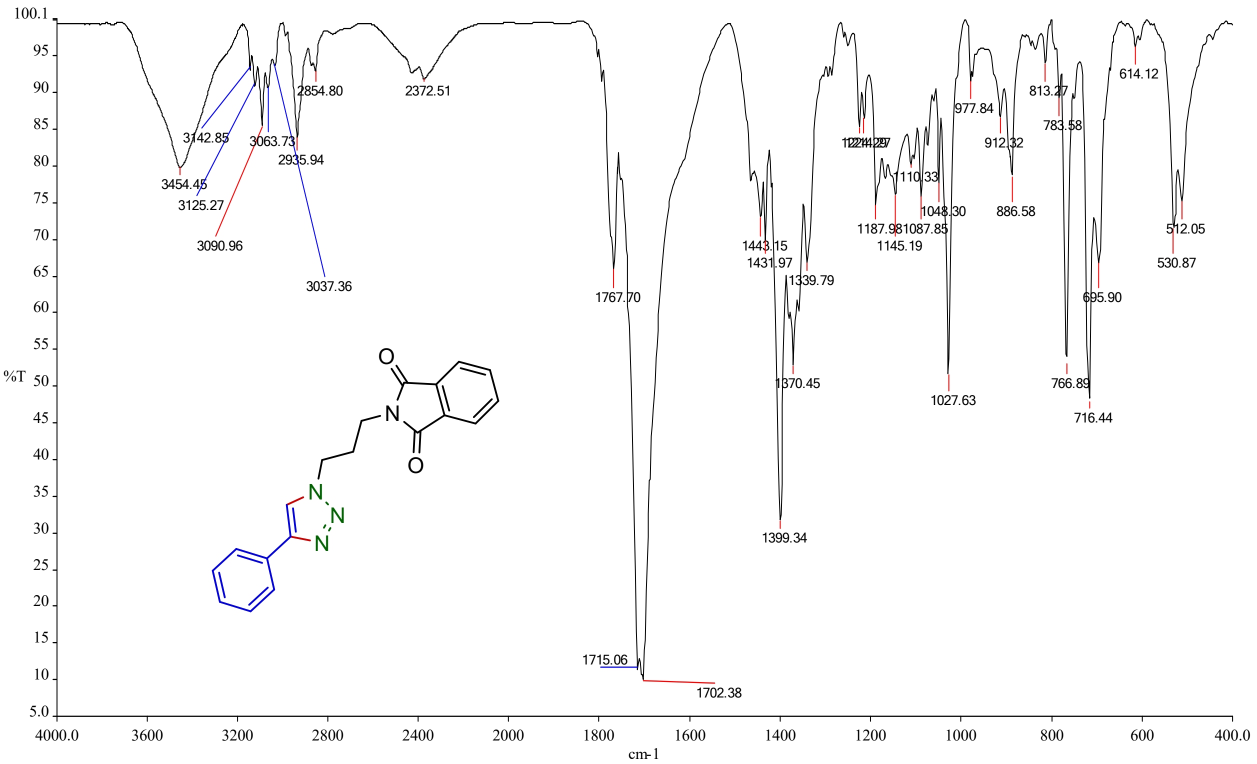

Figure S12. FT-IR spectrum of (**4b**) in KBr

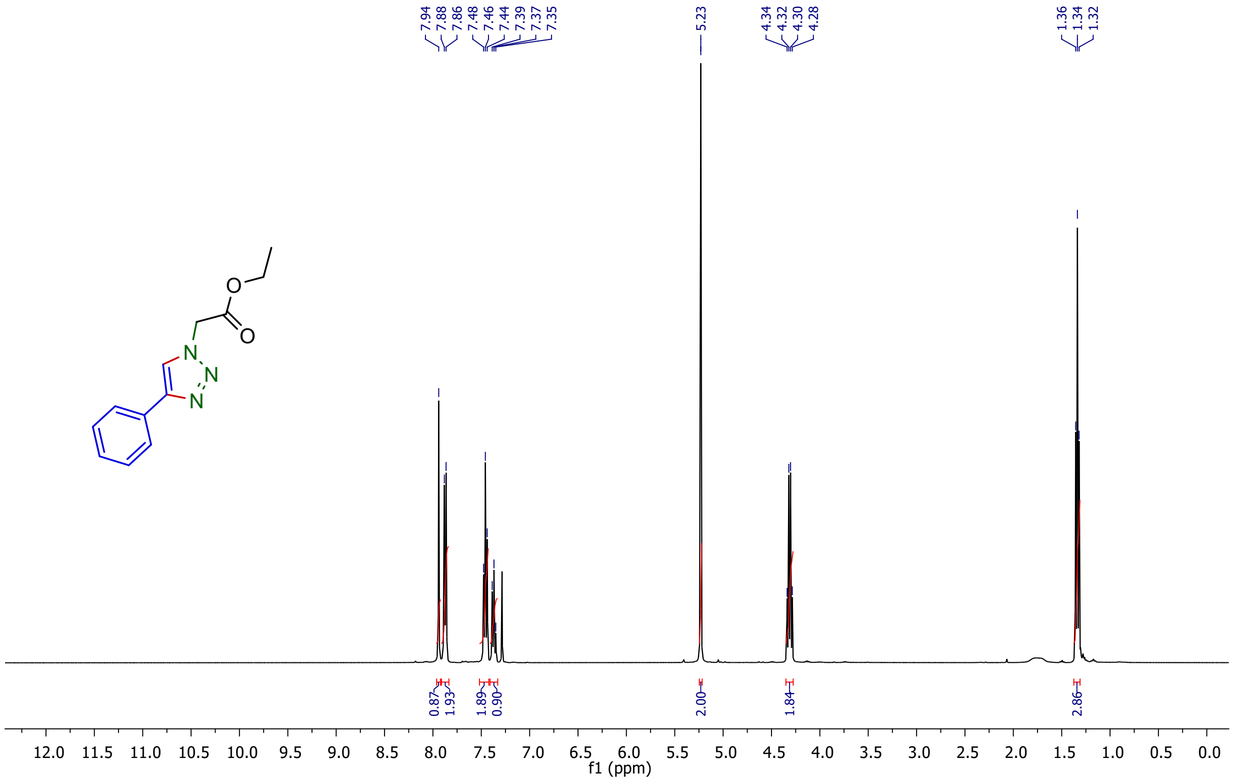

Figure S13. ^{1}H NMR spectrum of (**4c**) in $CDCl_3$ (400 MHz).

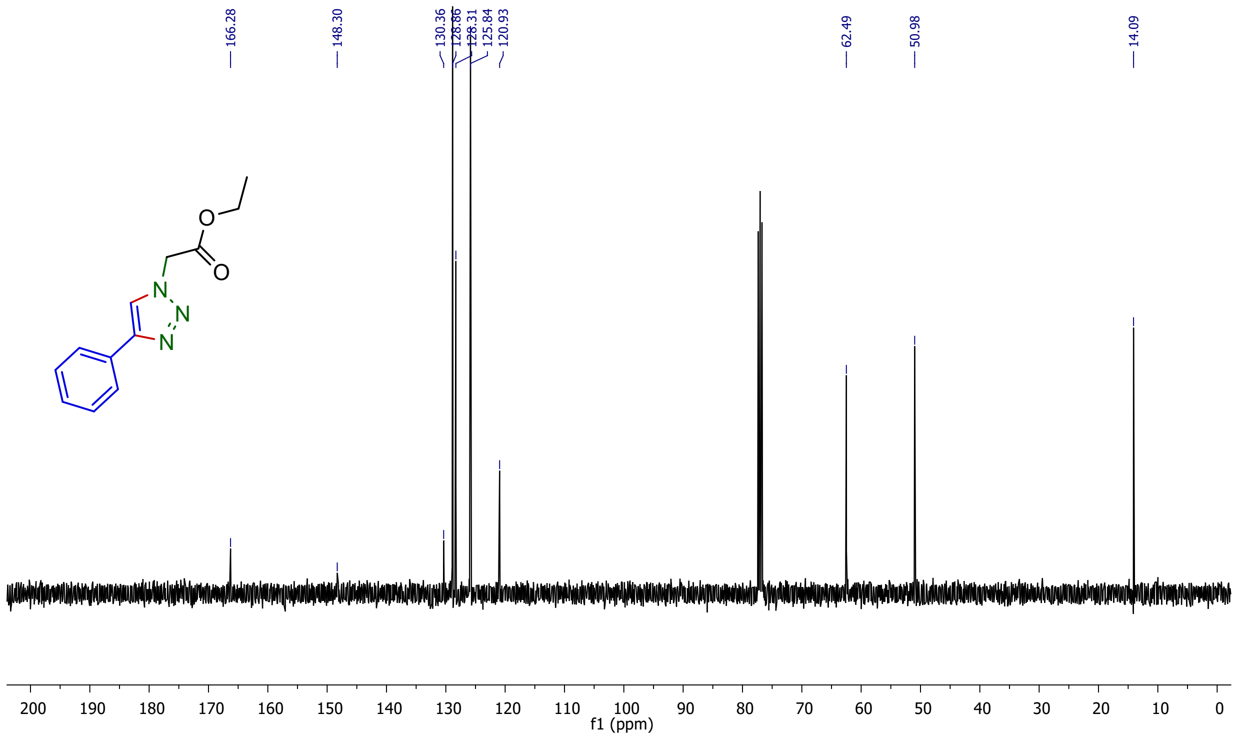

Figure S14. $^{13}C\{^{1}H\}$ NMR spectrum of (**4c**) in $CDCl_3$ (101 MHz).

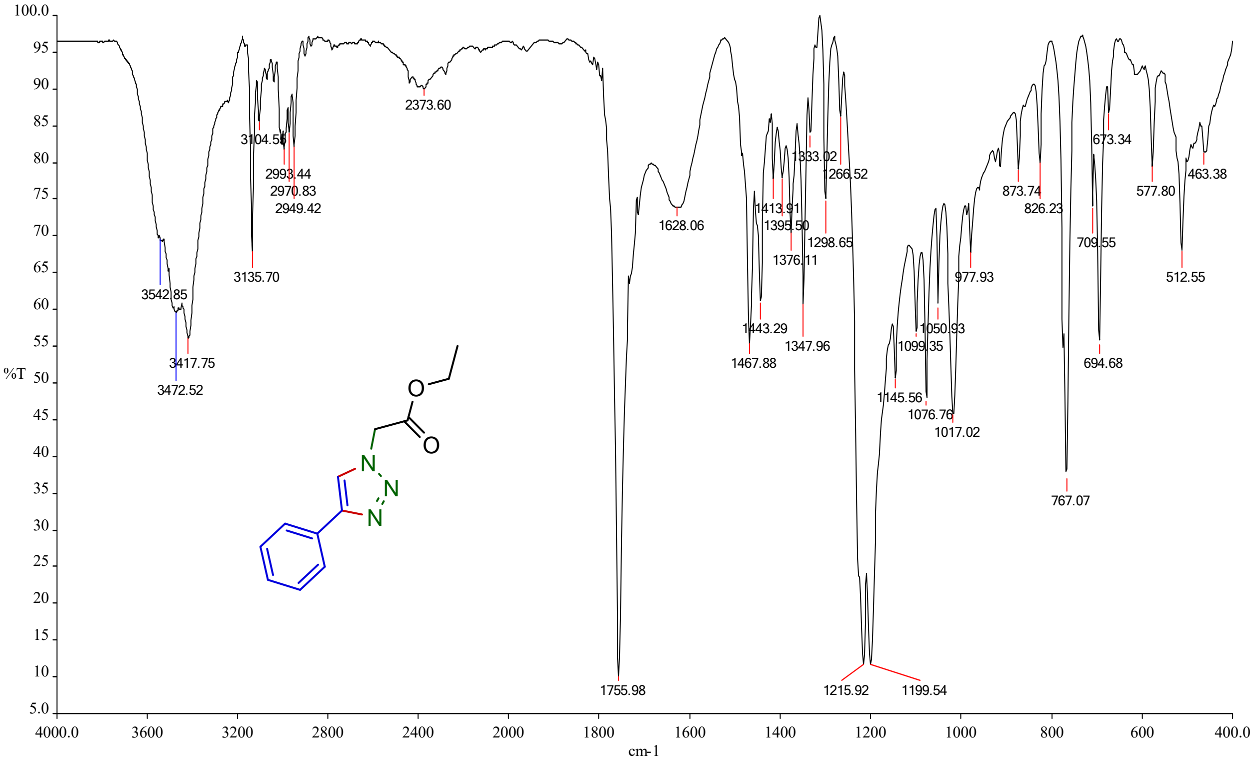

Figure S15. FT-IR spectrum of (**4c**) in KBr

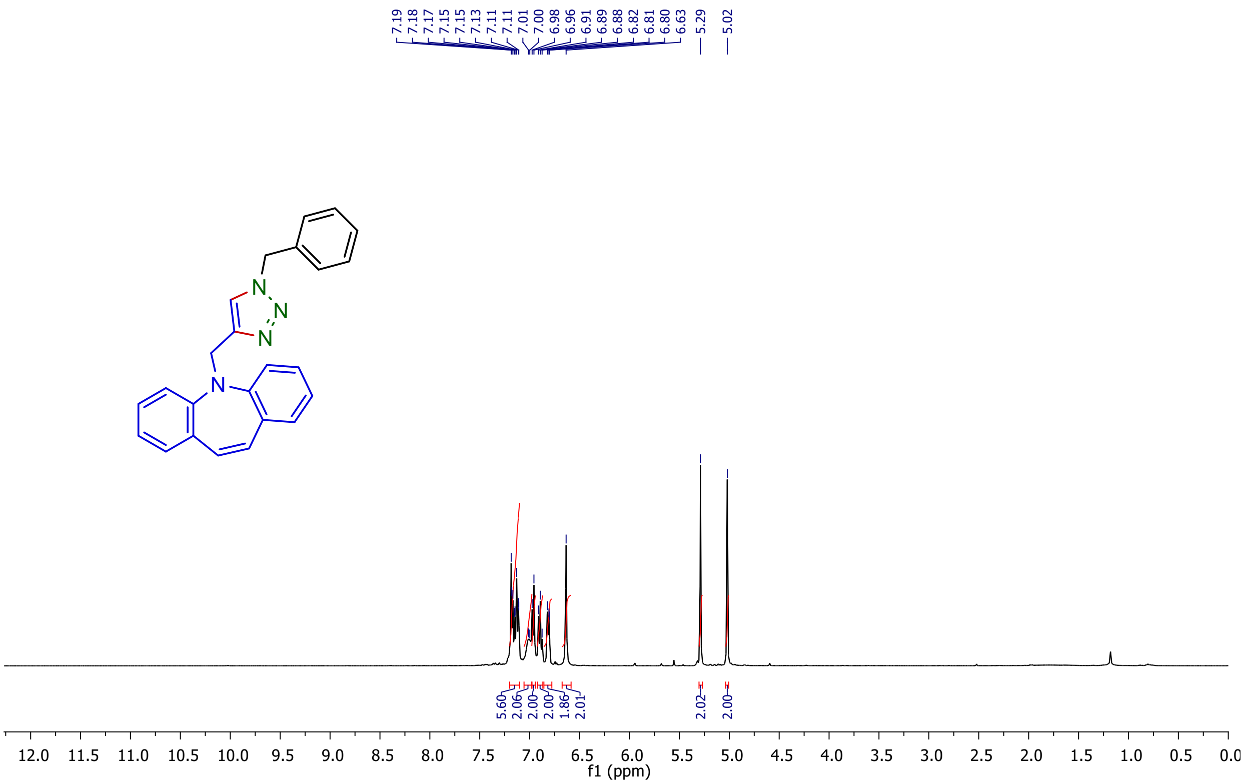

Figure S16. ^{1}H NMR spectrum of (**4d**) in $CDCl_3$ (400 MHz).

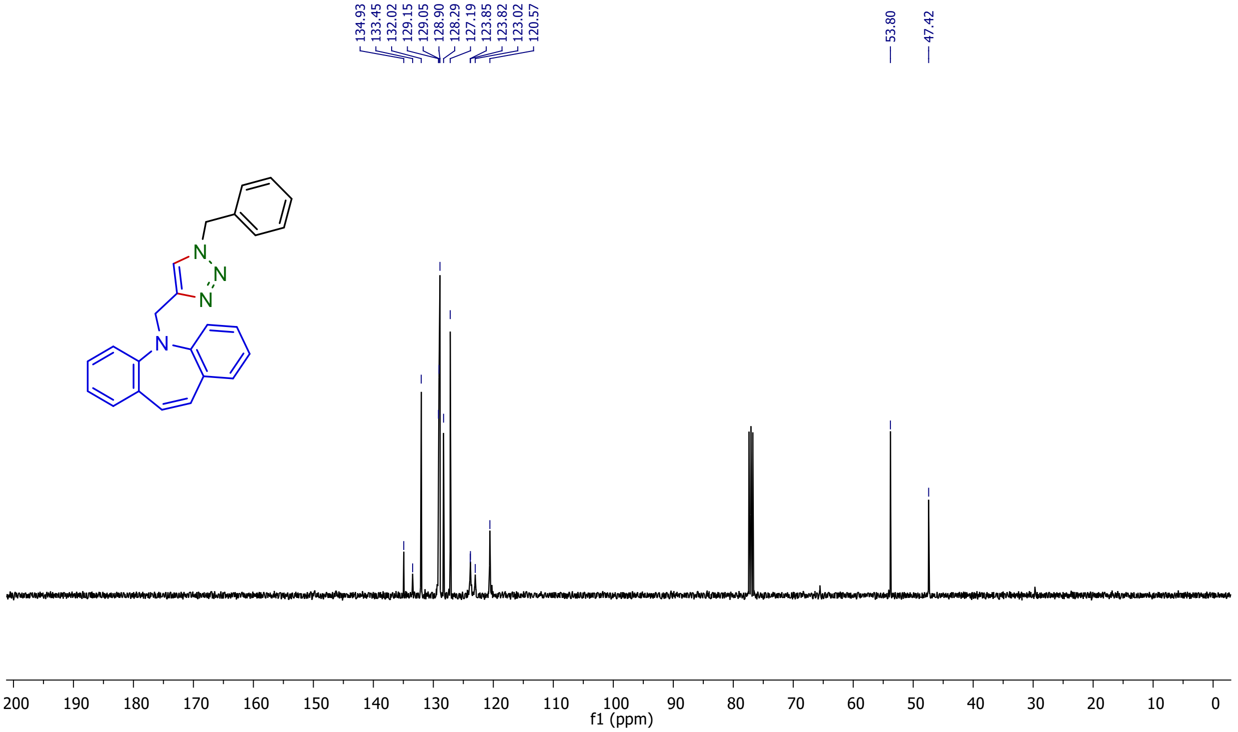

Figure S17. $^{13}C\{^{1}H\}$ NMR spectrum of (**4d**) in $CDCl_3$ (101 MHz).

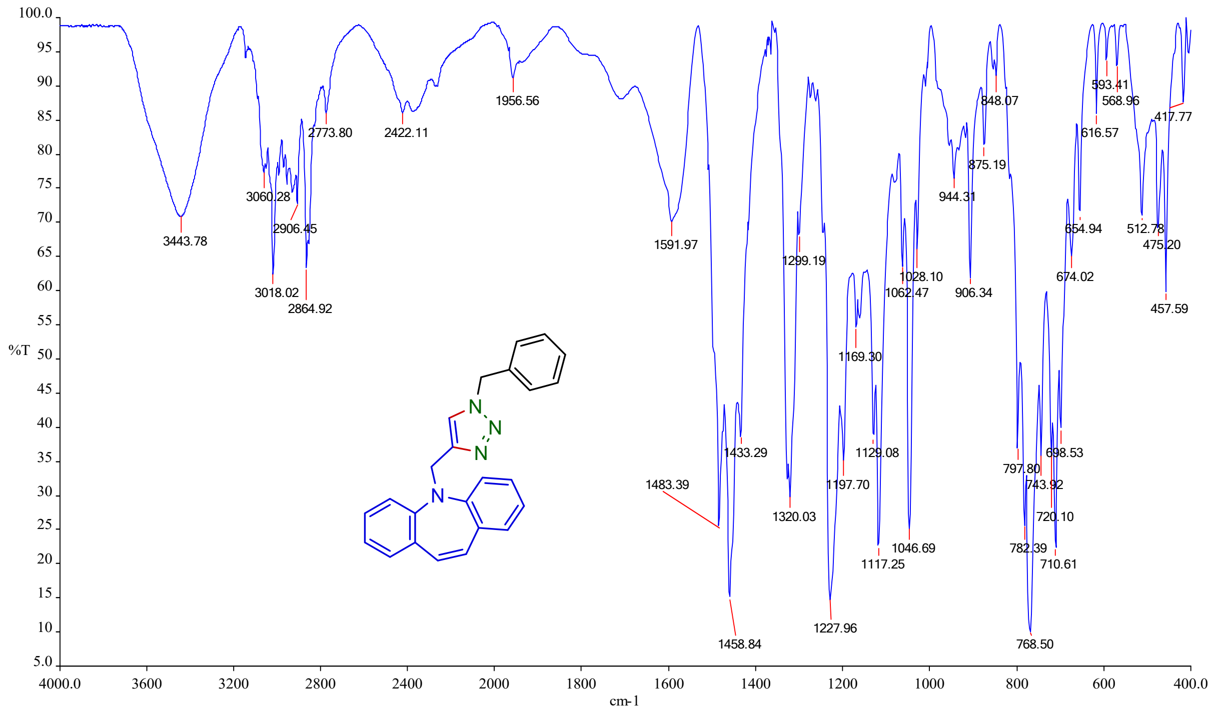

Figure S18. FT-IR spectrum of (**4d**) in KBr

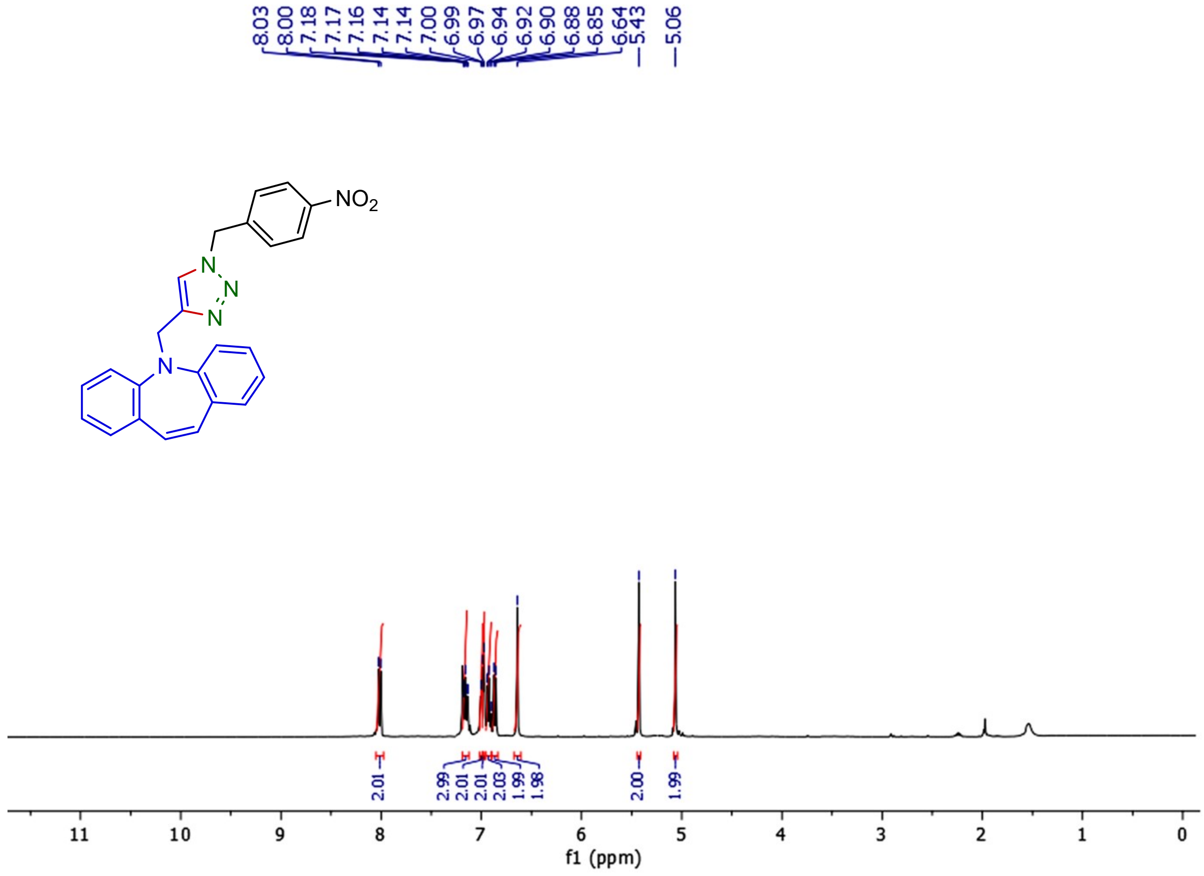

Figure S19. ^{1}H NMR spectrum of (**4e**) in $CDCl_3$ (400 MHz).

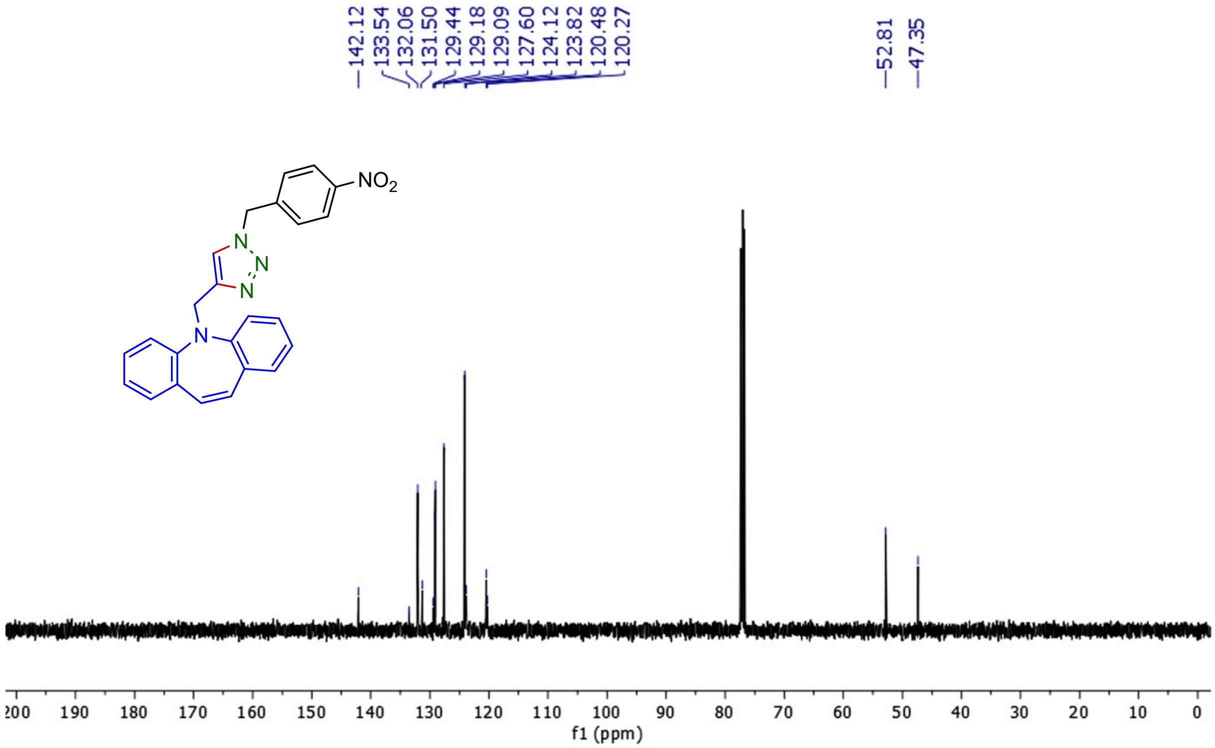

Figure S20. $^{13}C\{^{1}H\}$ NMR spectrum of (**4e**) in $CDCl_3$ (101 MHz).

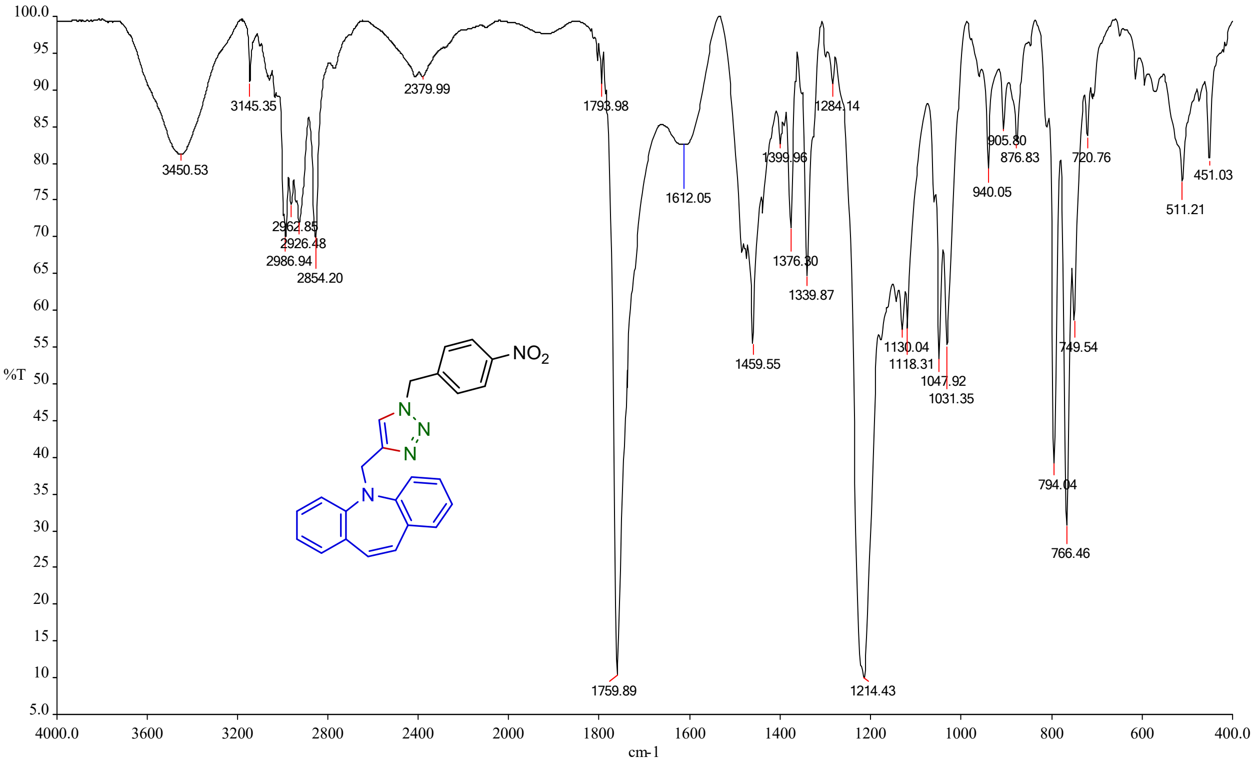

Figure S21. FT-IR spectrum of (**4e**) in KBr

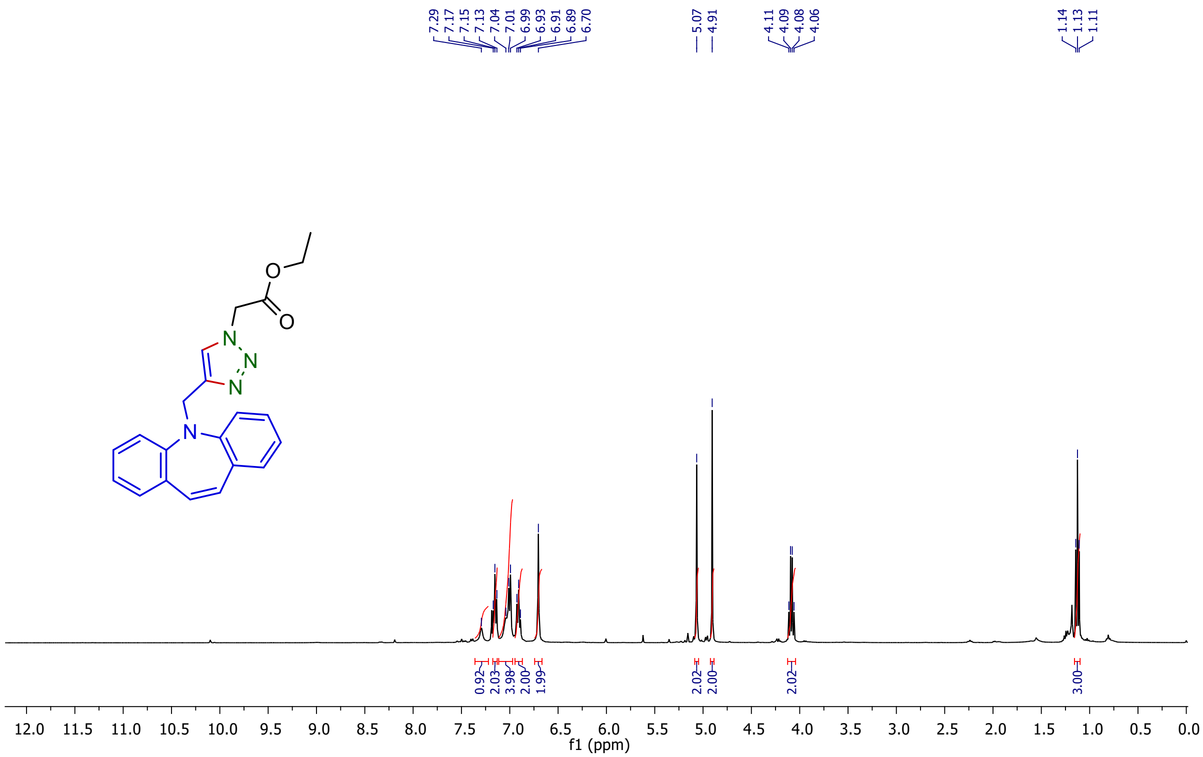

Figure S22. ^{1}H NMR spectrum of (**4f**) in $CDCl_3$ (400 MHz).

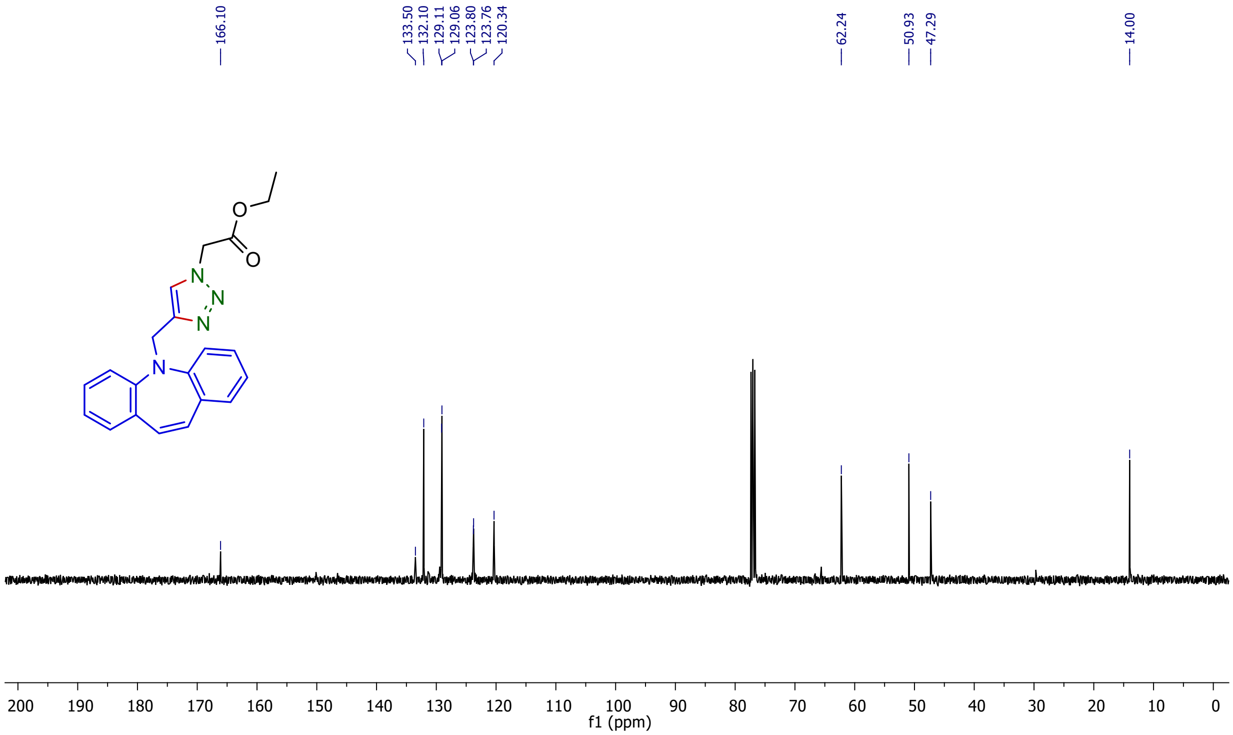

Figure S23. $^{13}C\{^{1}H\}$ NMR spectrum of (**4f**) in $CDCl_3$ (101 MHz).

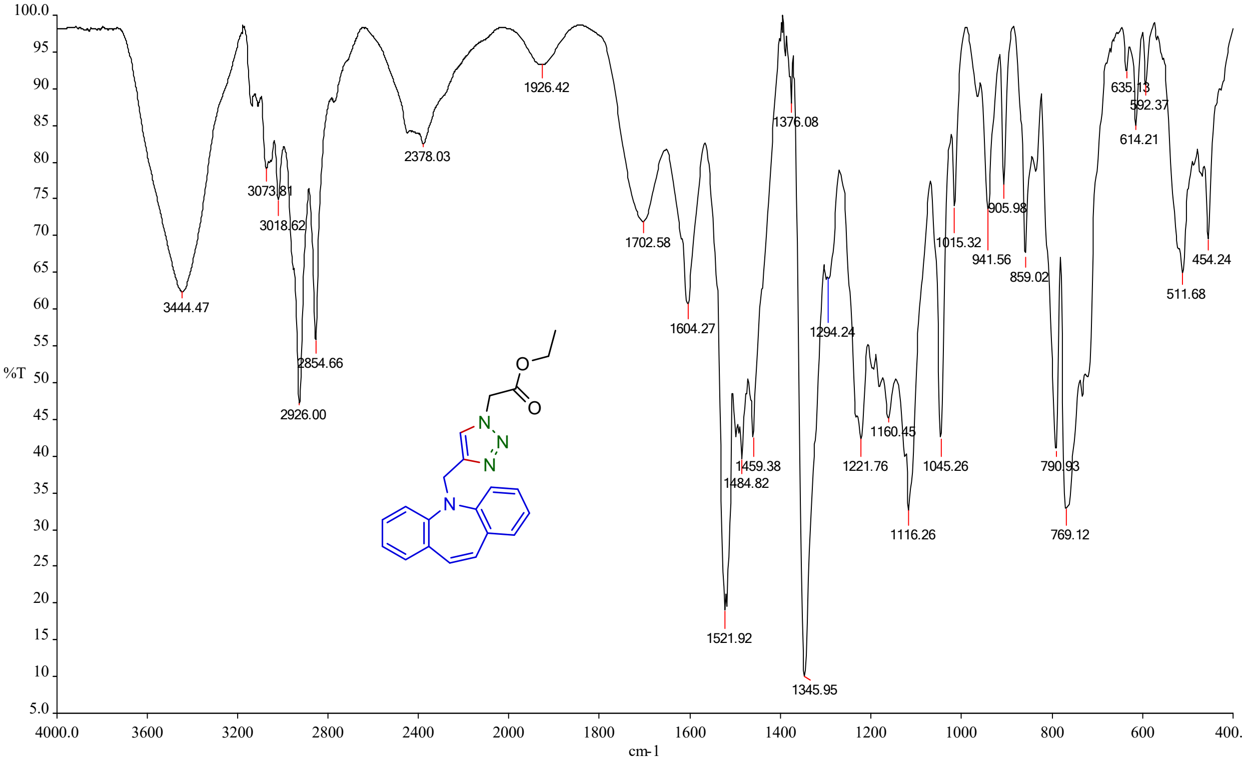

Figure S24. FT-IR spectrum of (**4f**) in KBr

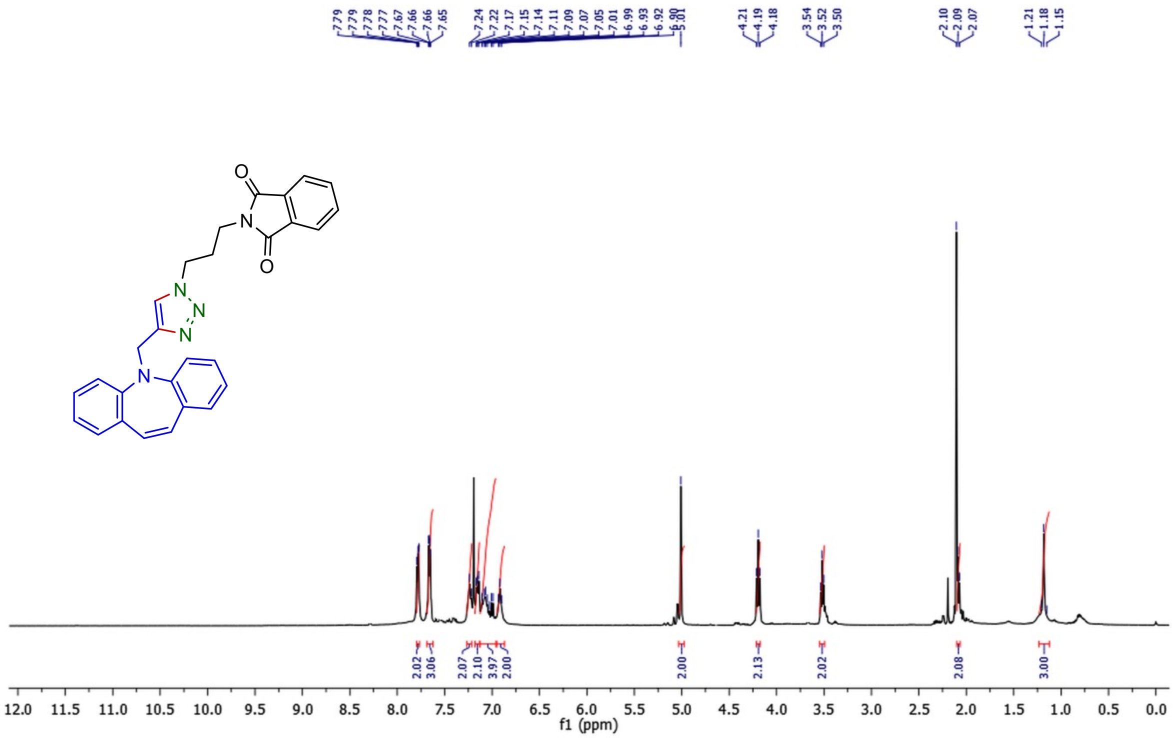

Figure S25. ^{1}H NMR spectrum of (**4g**) in $CDCl_3$ (400 MHz).

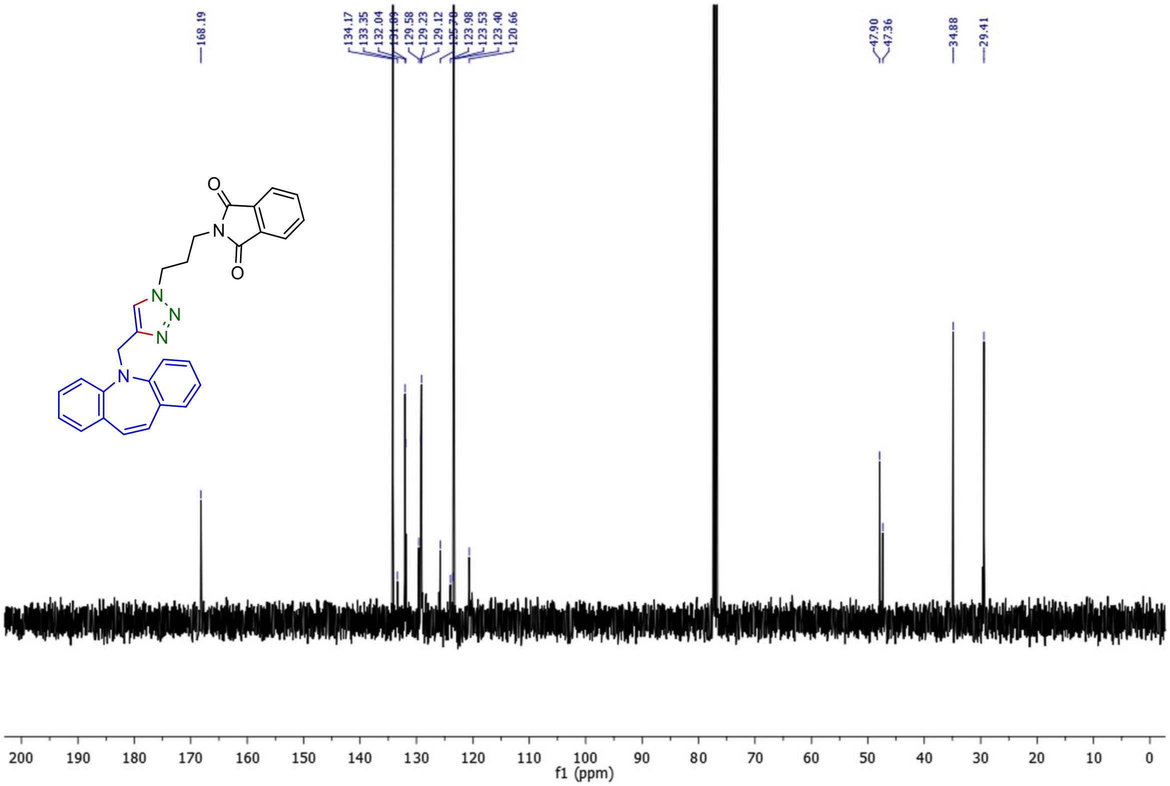

Figure S26. $^{13}C\{^{1}H\}$ NMR spectrum of (**4g**) in $CDCl_3$ (101 MHz).

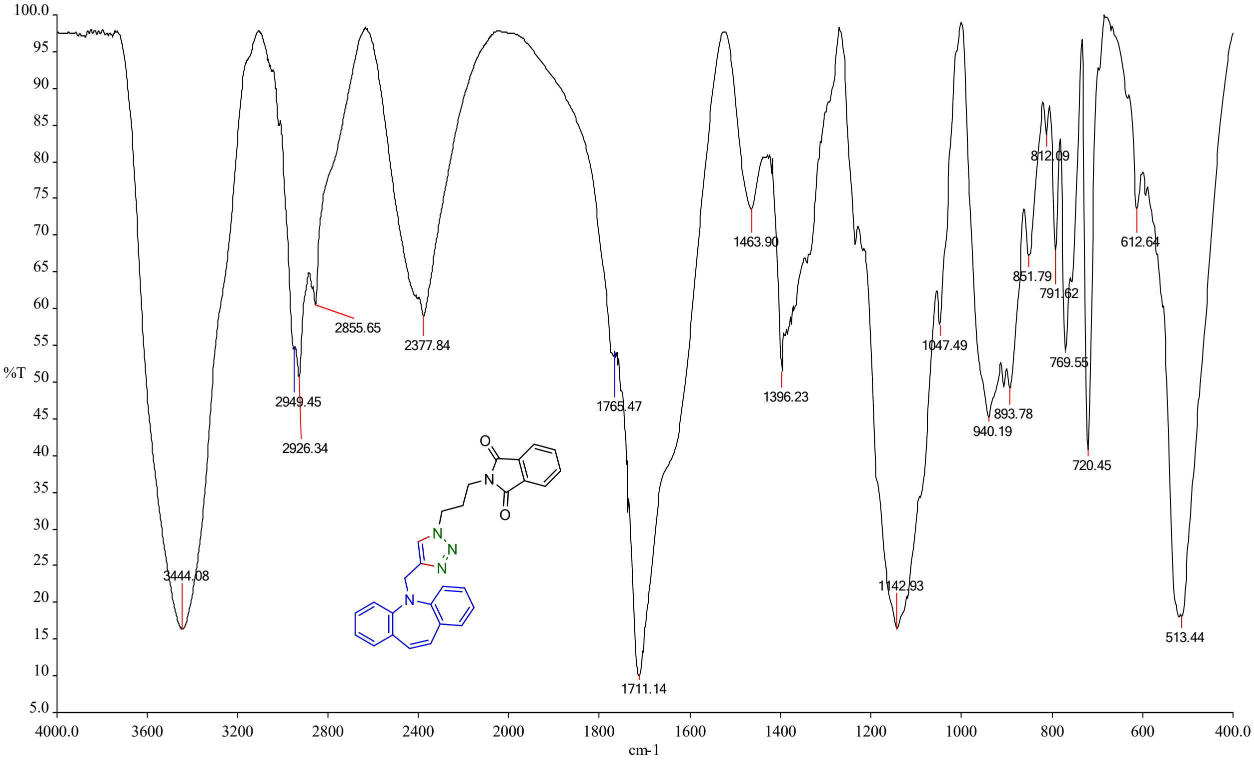

Figure S27. FT-IR spectrum of (**4g**) in KBr

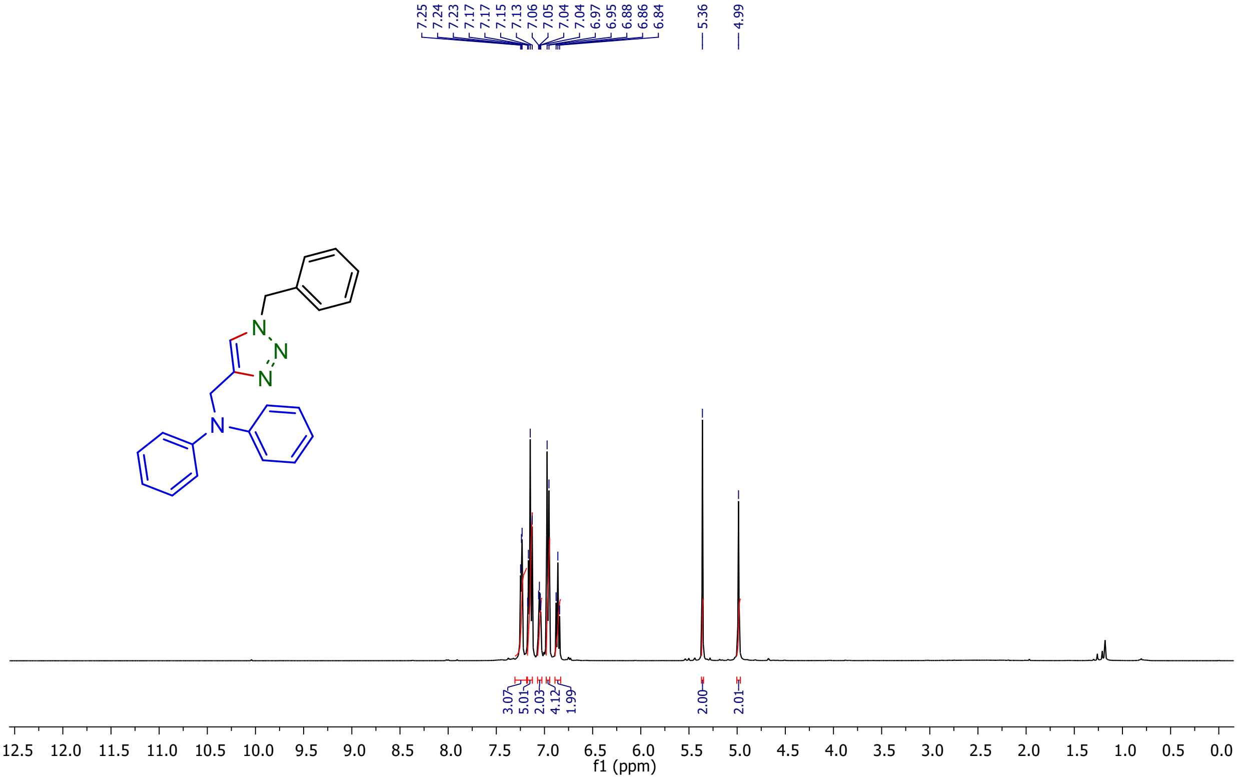

Figure S28. ^{1}H NMR spectrum of (**4h**) in $CDCl_3$ (400 MHz).

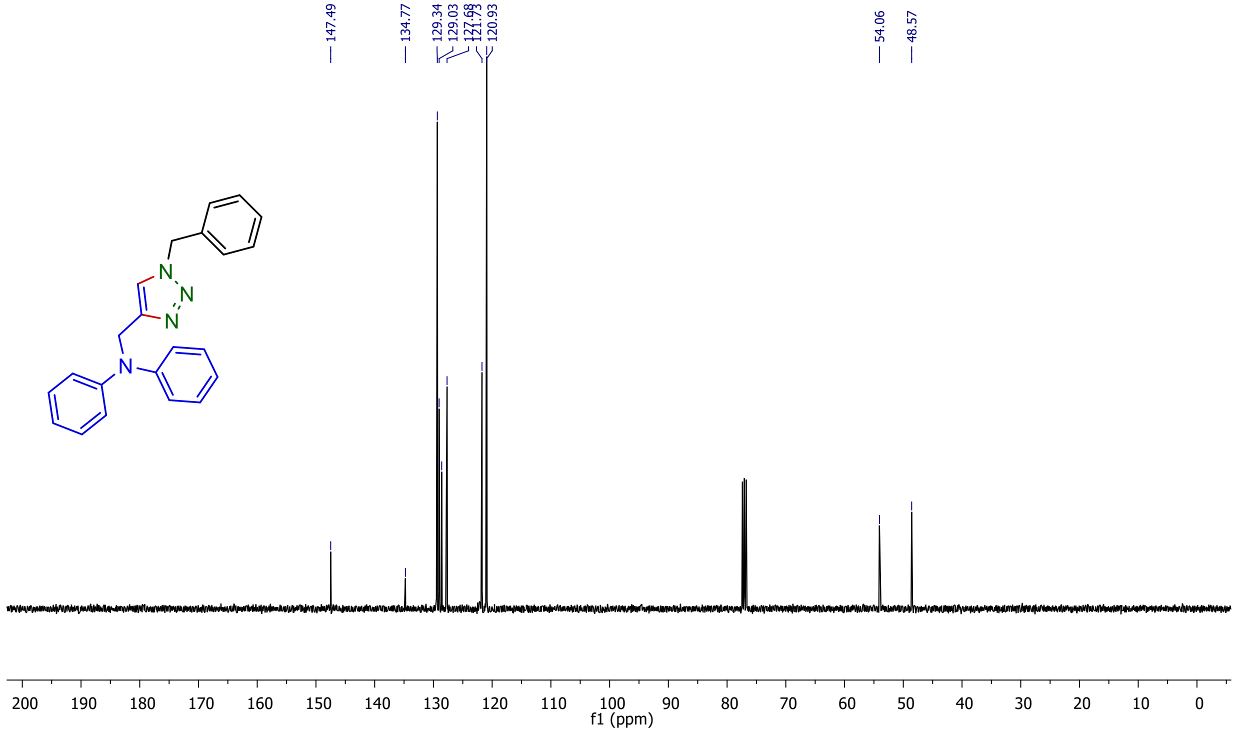

Figure S29. $^{13}C\{^{1}H\}$ NMR spectrum of (**4h**) in $CDCl_3$ (101 MHz).

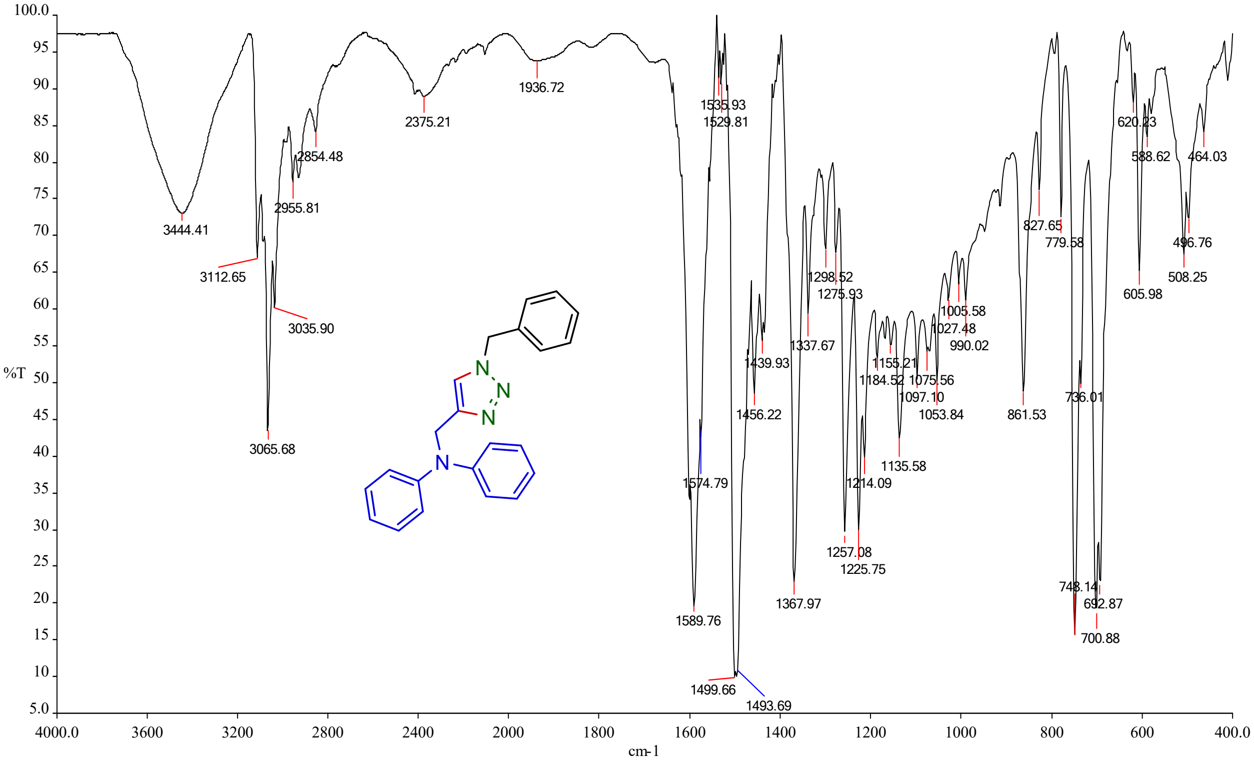

Figure S30. FT-IR spectrum of (**4h**) in KBr

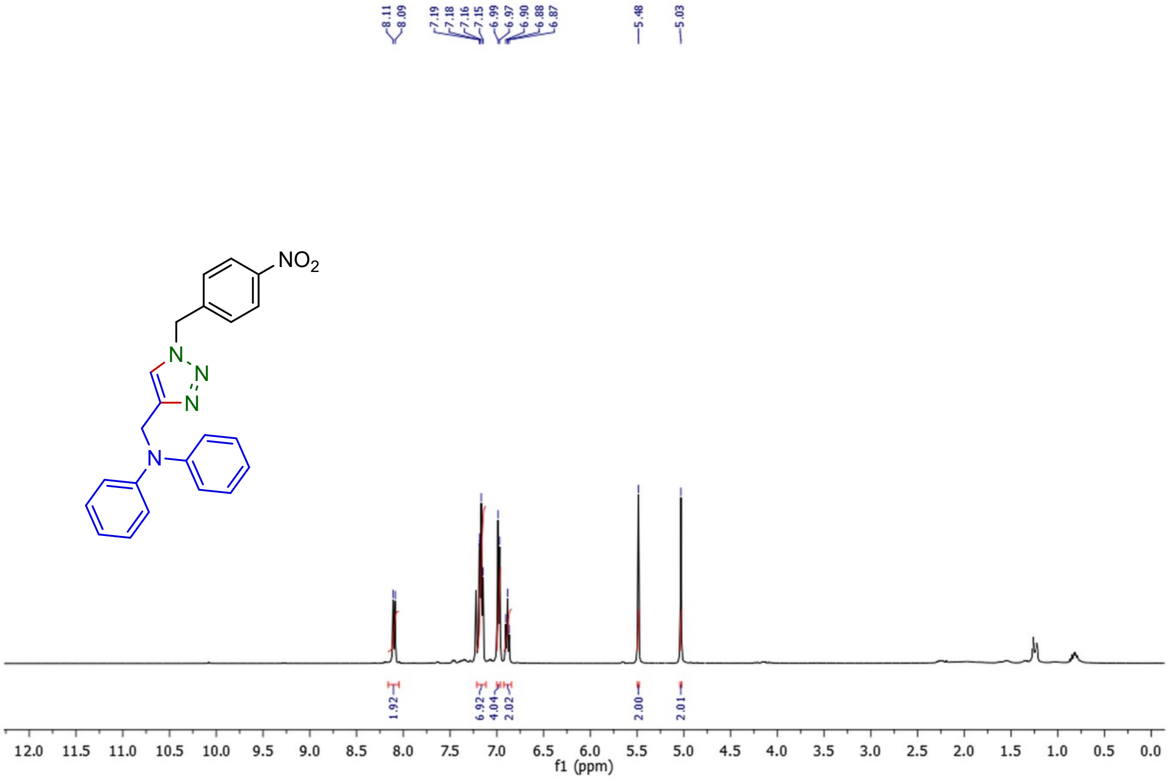

Figure S31. ^{1}H NMR spectrum of (**4i**) in $CDCl_3$ (400 MHz).

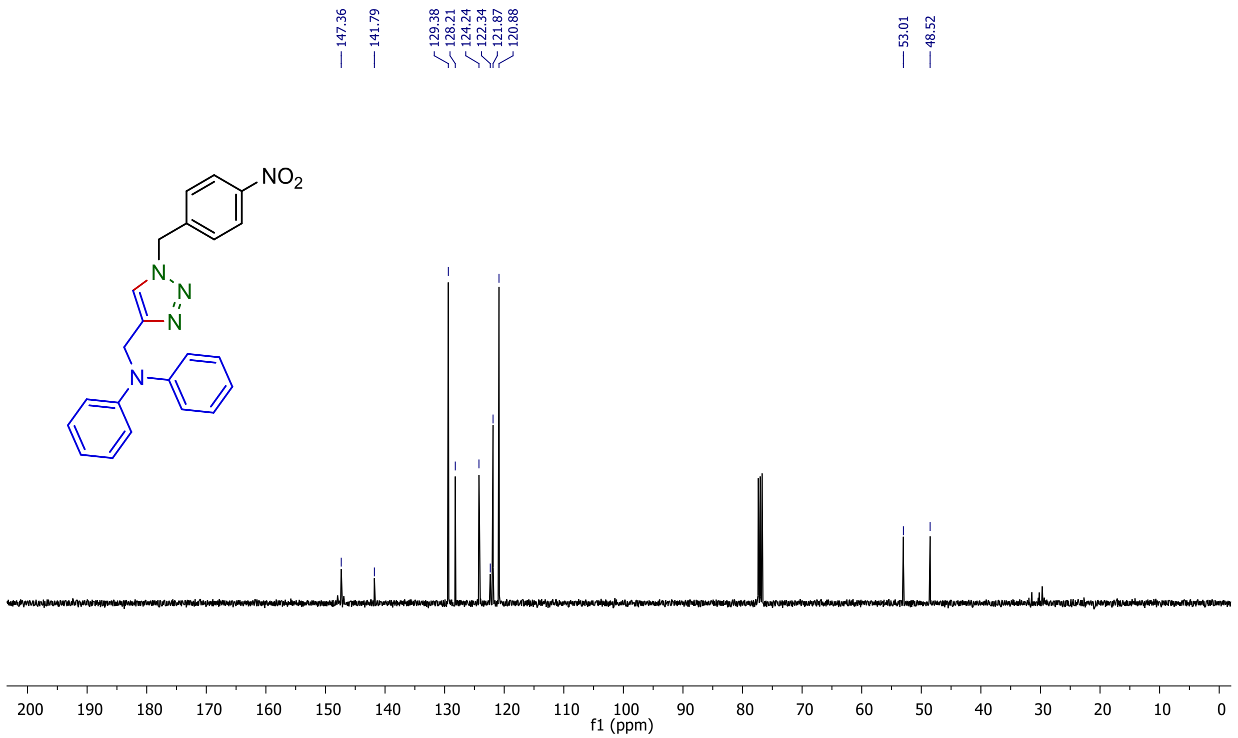

Figure S32. $^{13}C\{^{1}H\}$ NMR spectrum of (**4i**) in $CDCl_3$ (101 MHz).

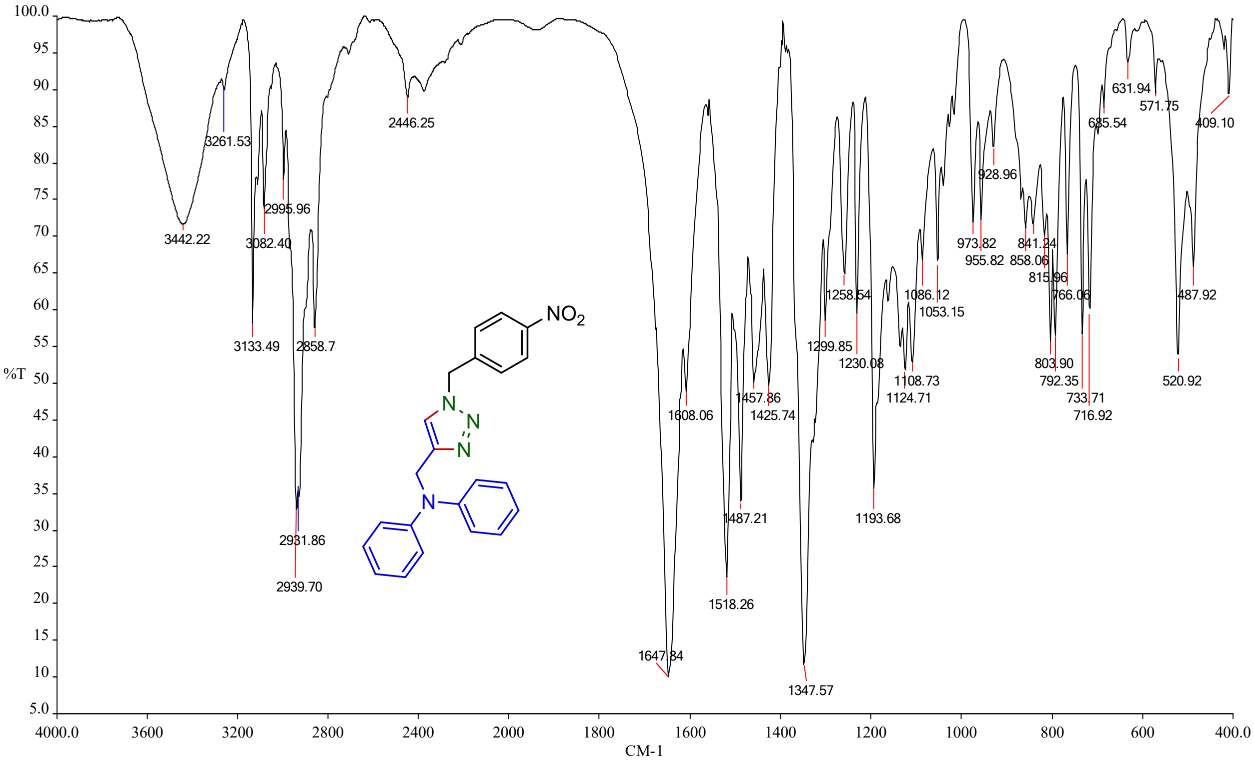

Figure S33. FT-IR spectrum of (**4i**) in KBr

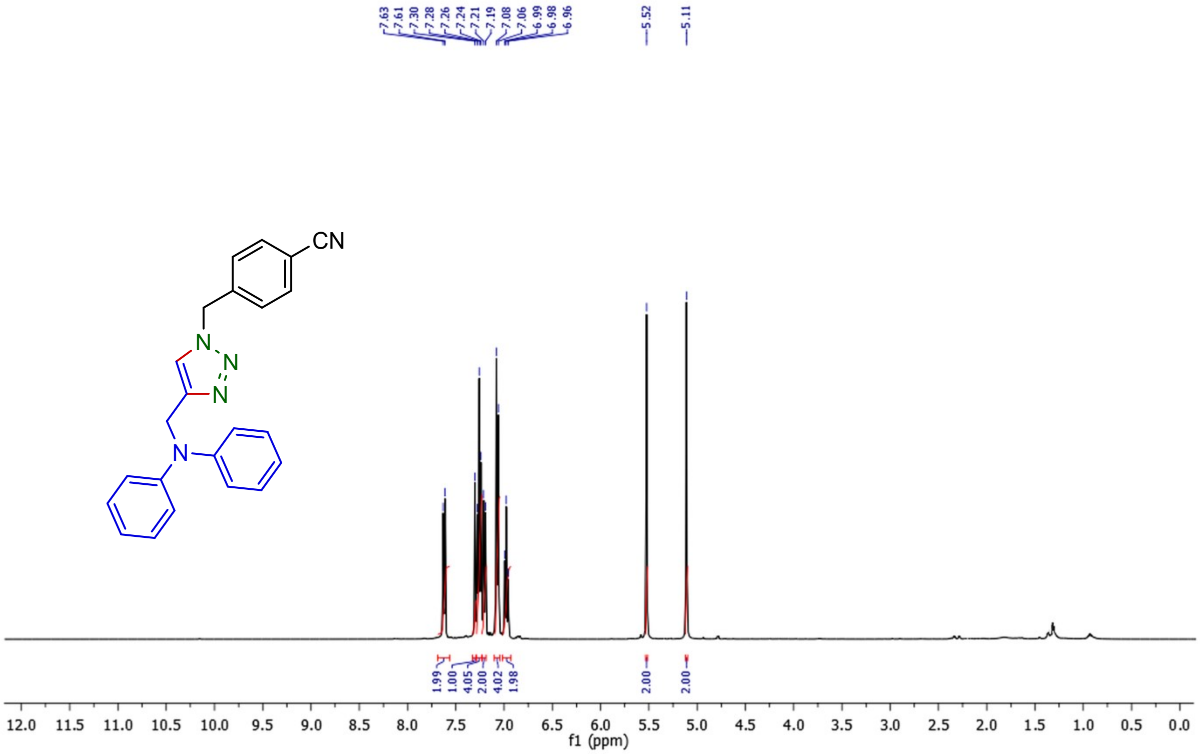

Figure S34. ^{1}H NMR spectrum of (**4j**) in $CDCl_3$ (400 MHz).

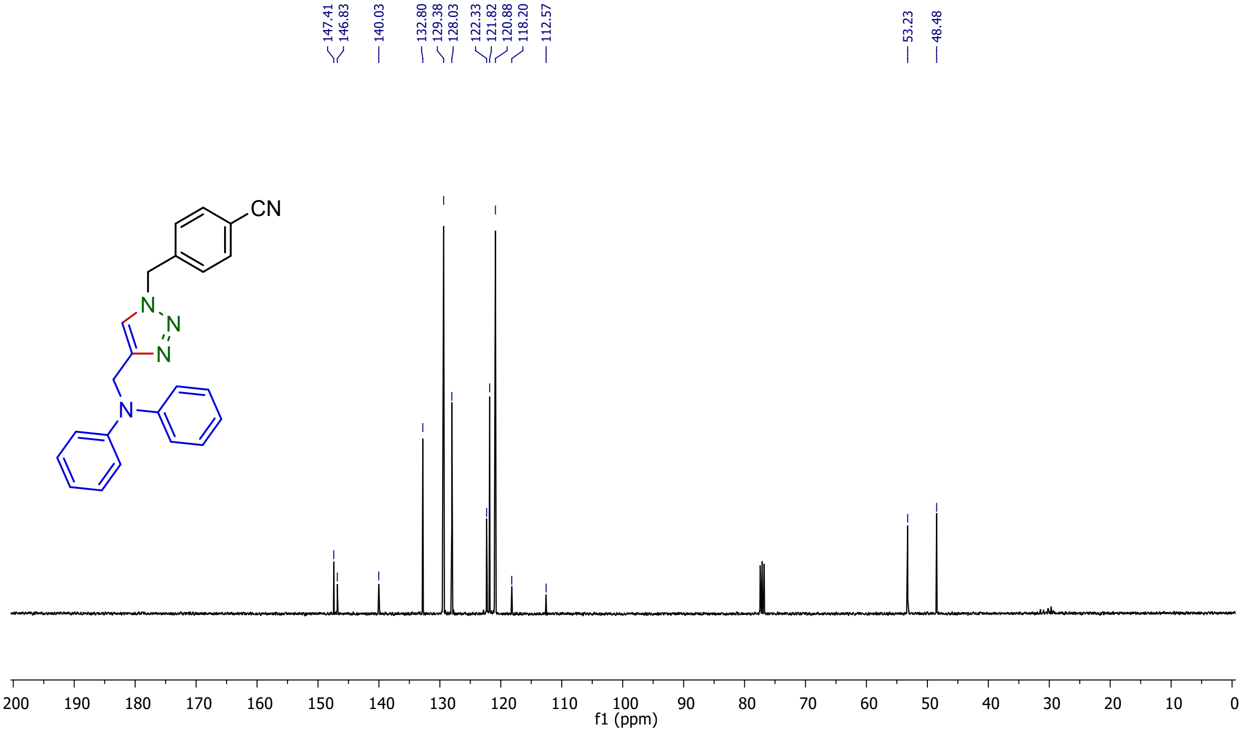

Figure S35. $^{13}C\{^{1}H\}$ NMR spectrum of (**4j**) in $CDCl_3$ (101 MHz).

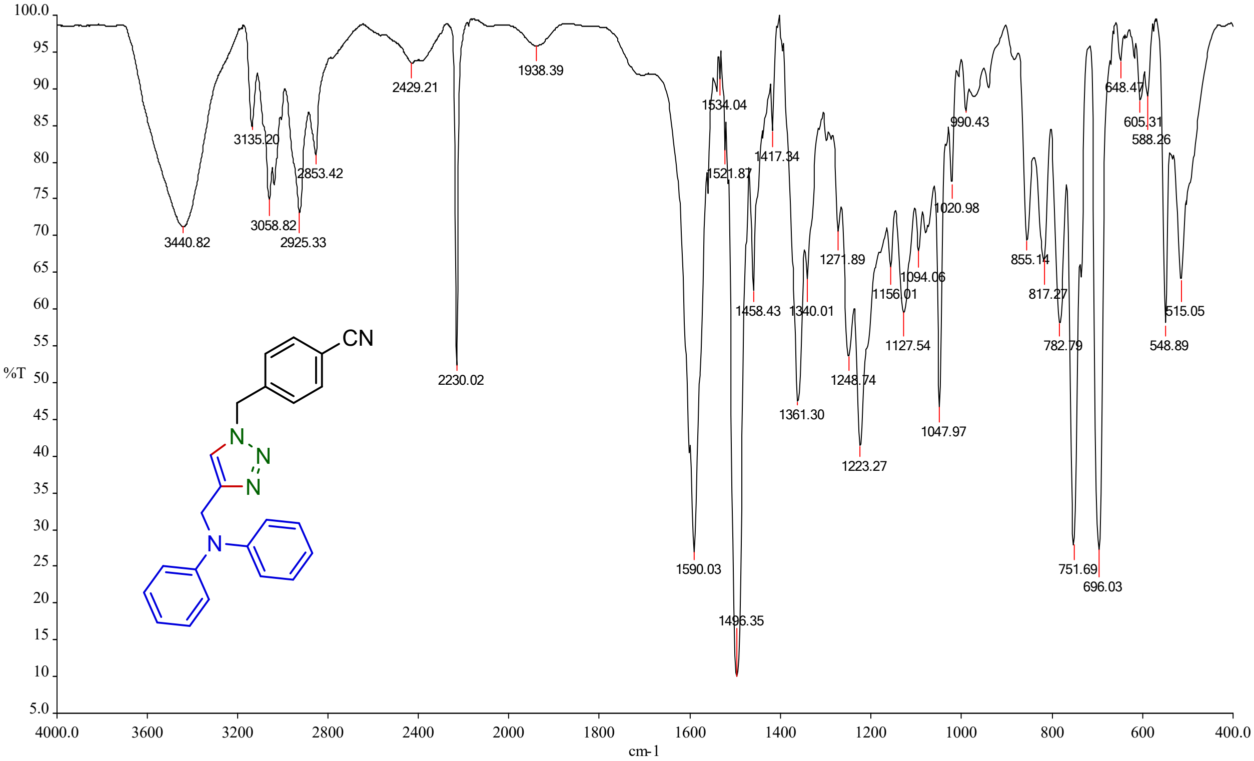

Figure S36. FT-IR spectrum of (**4j**) in KBr

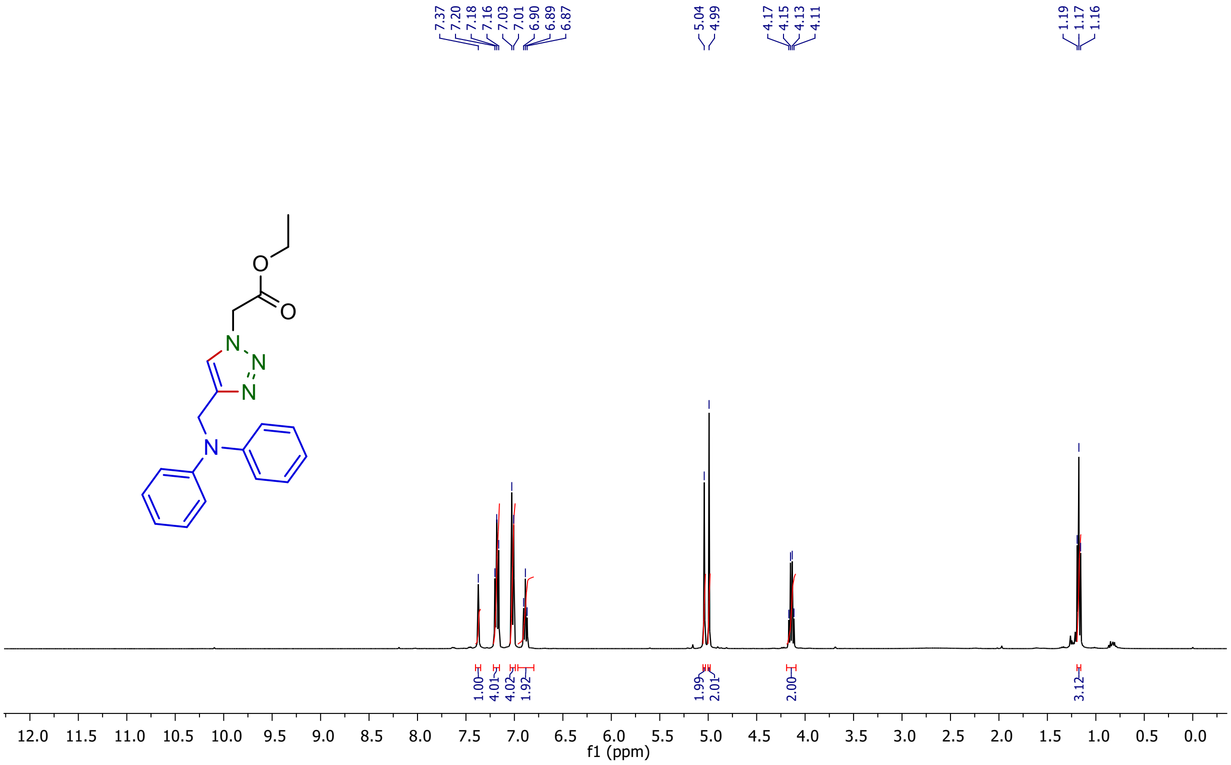

Figure S37. ^{1}H NMR spectrum of (**4k**) in $CDCl_3$ (400 MHz).

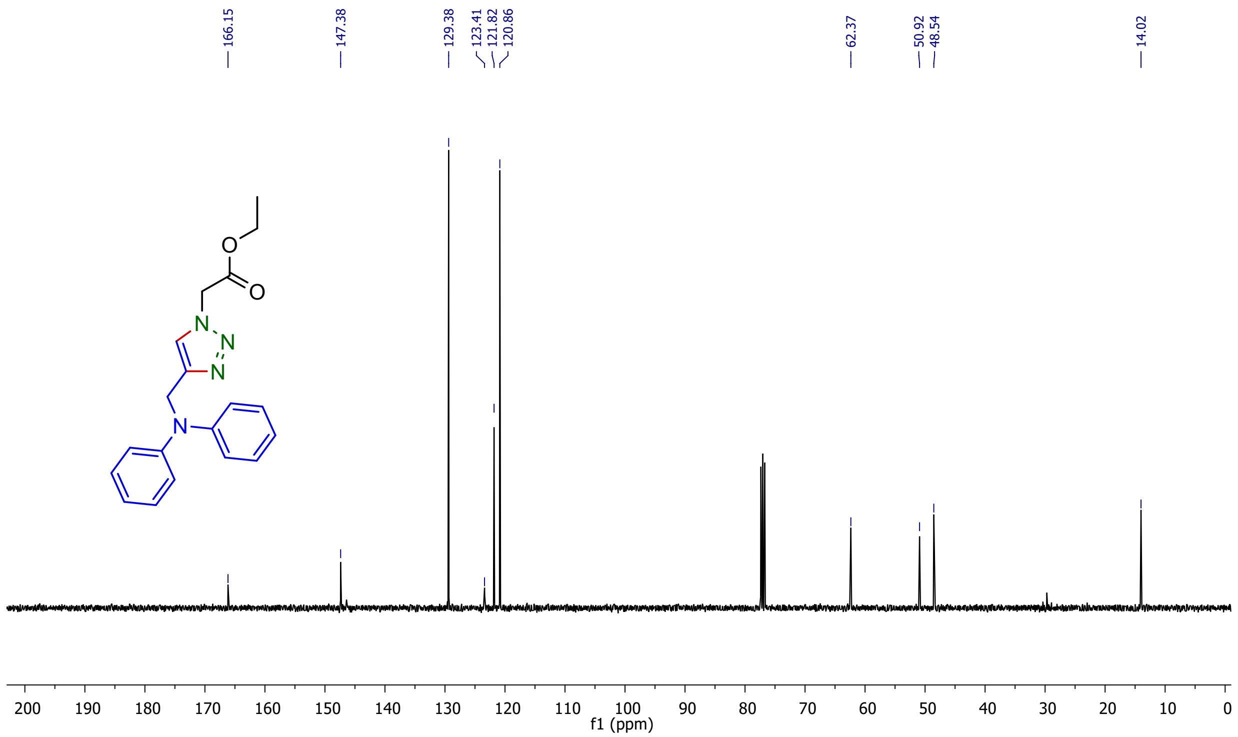

Figure S38. $^{13}C\{^{1}H\}$ NMR spectrum of (**4k**) in $CDCl_3$ (101 MHz).

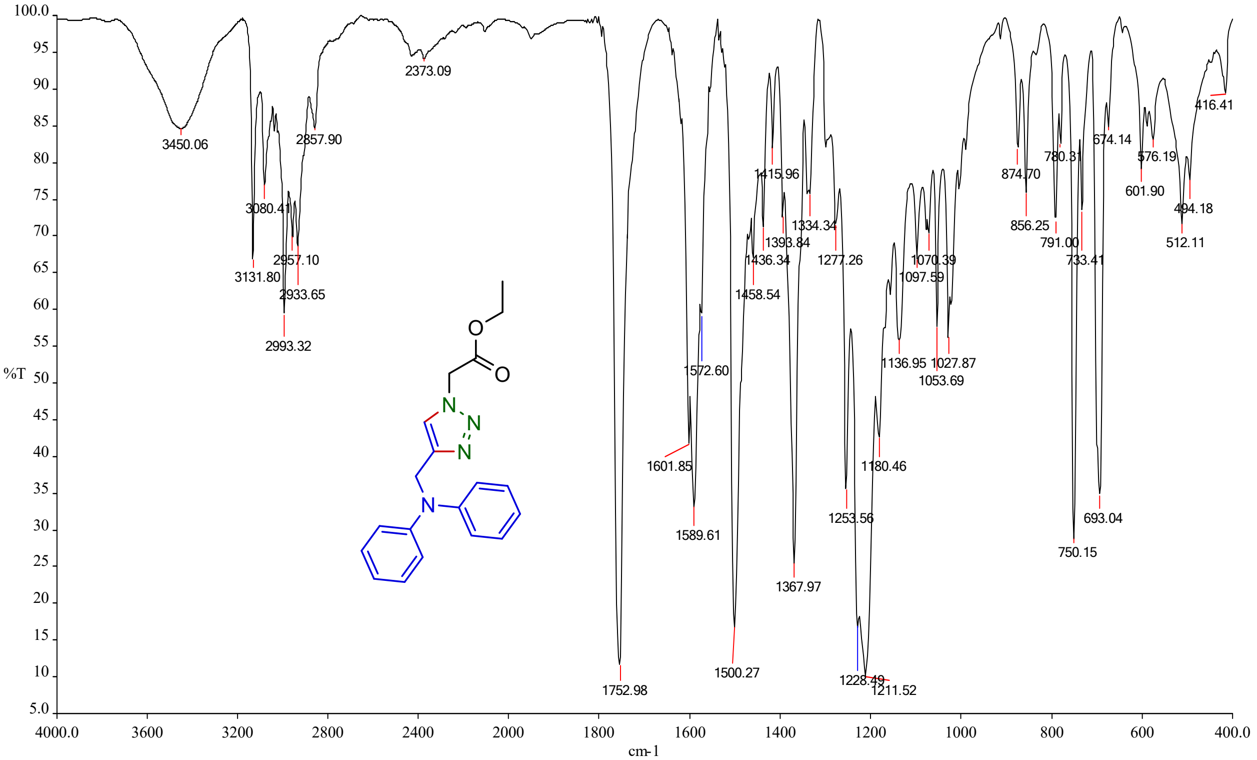

Figure S39. FT-IR spectrum of (**4k**) in KBr

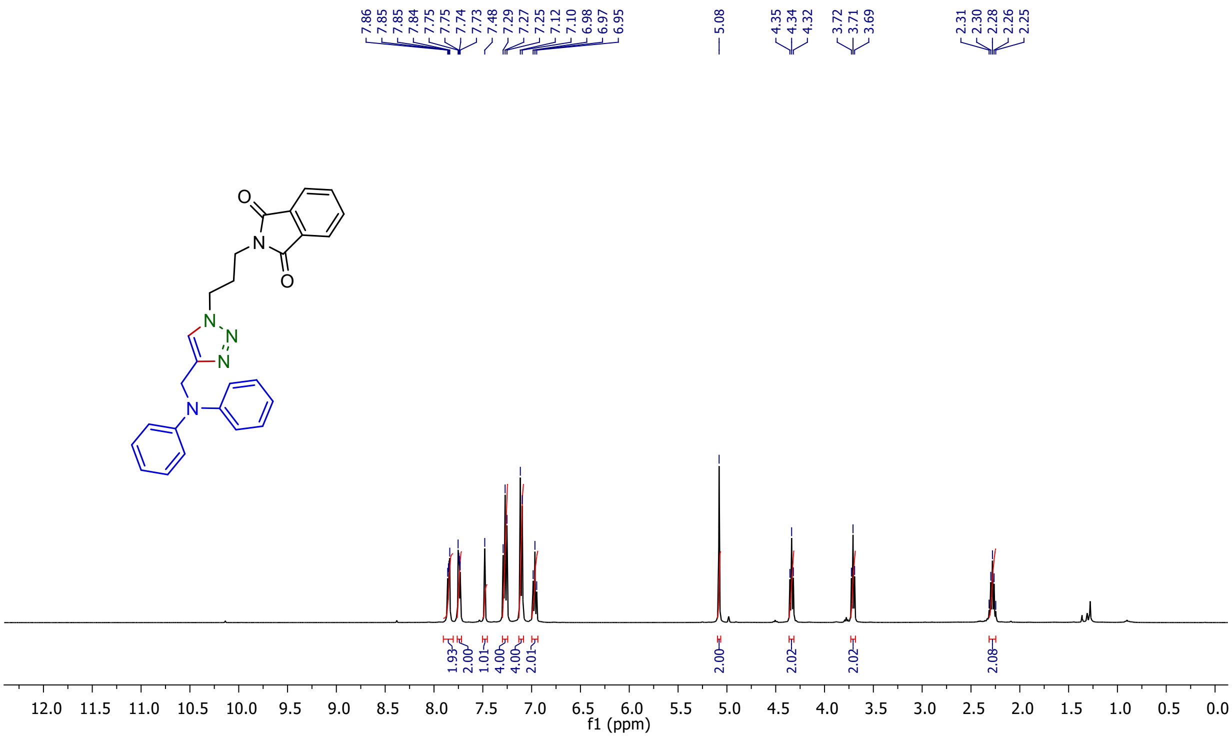

Figure S40. ^{1}H NMR spectrum of (**4l**) in $CDCl_3$ (400 MHz).

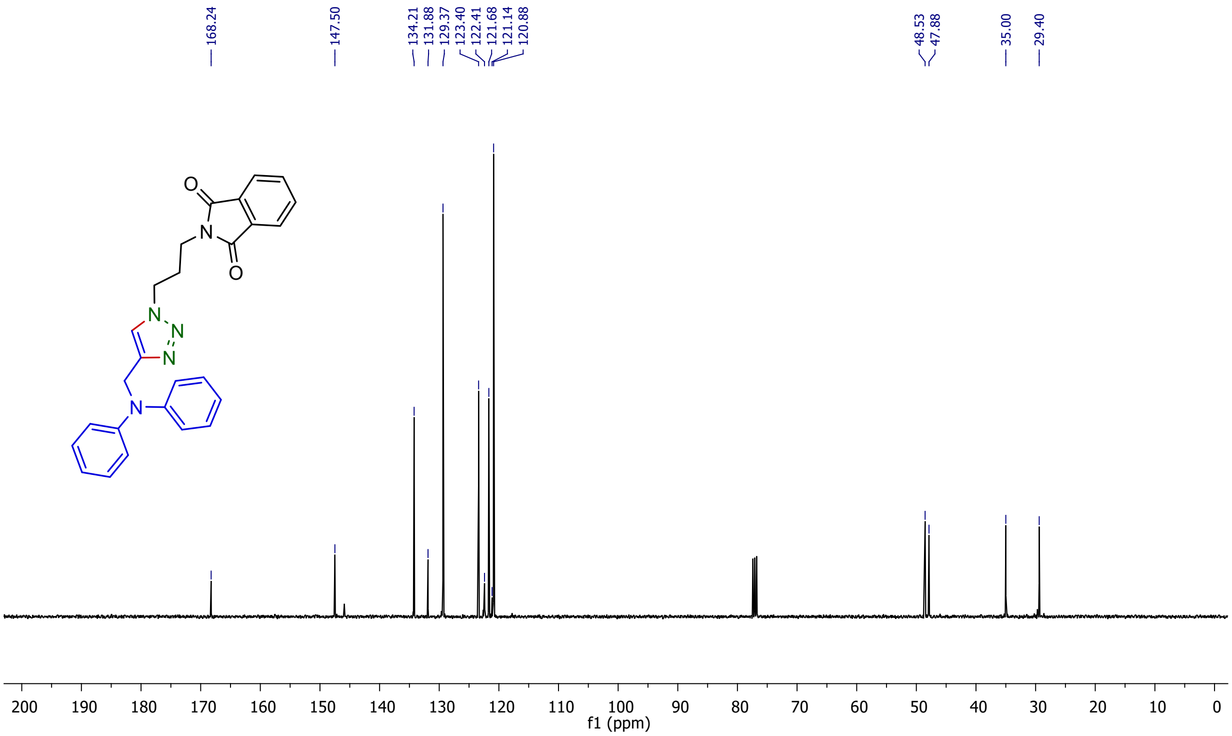

Figure S41. $^{13}C\{^{1}H\}$ NMR spectrum of (**4l**) in $CDCl_3$ (101 MHz).

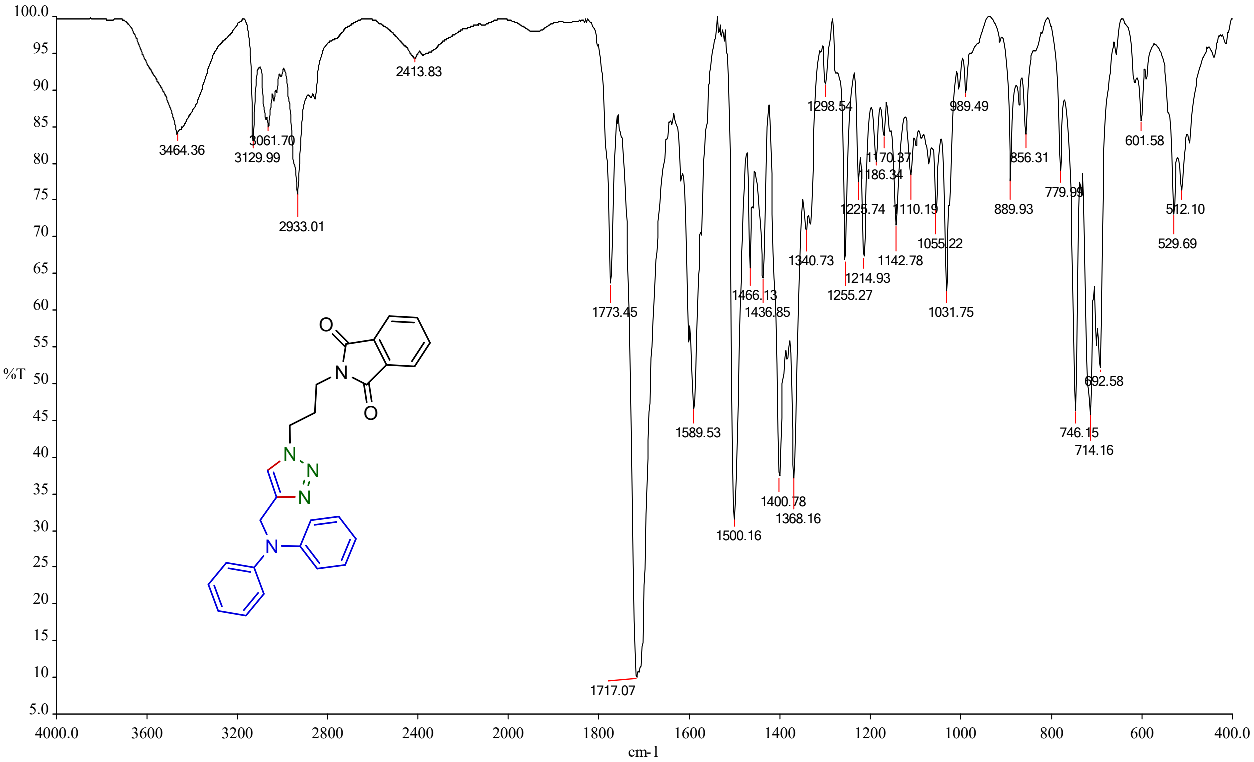

Figure S42. FT-IR spectrum of (**4l**) in KBr

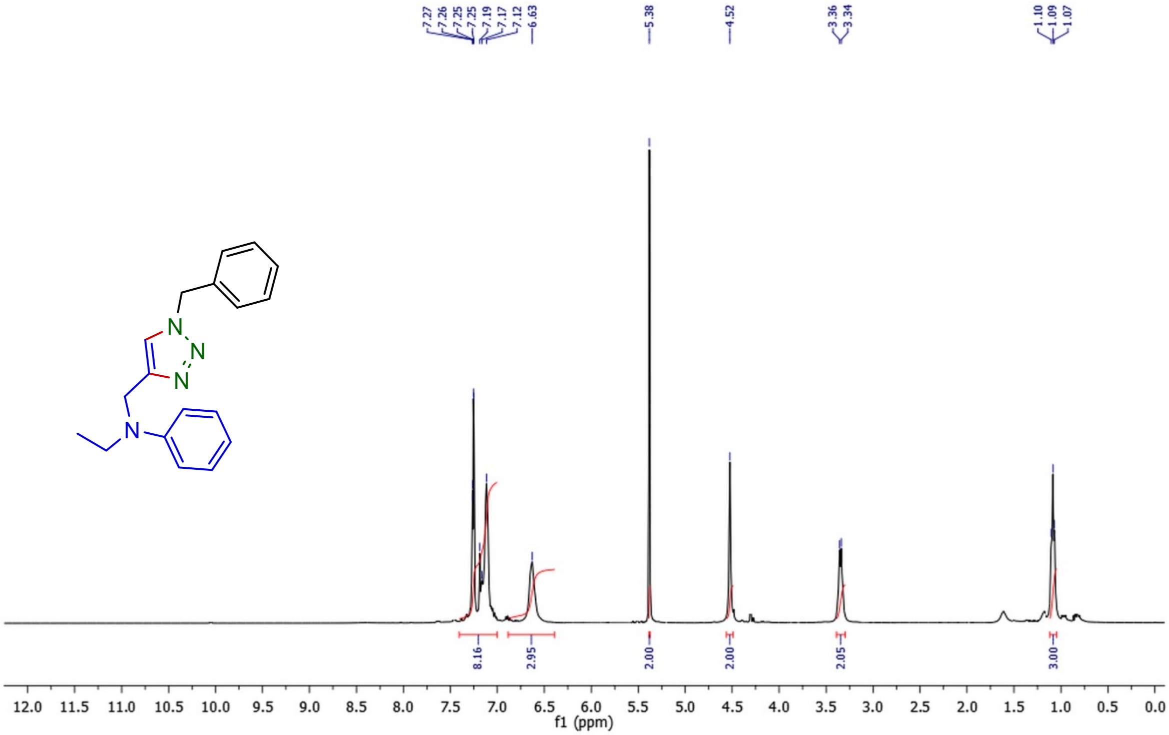

Figure S43. ^{1}H NMR spectrum of (**4m**) in $CDCl_3$ (400 MHz).

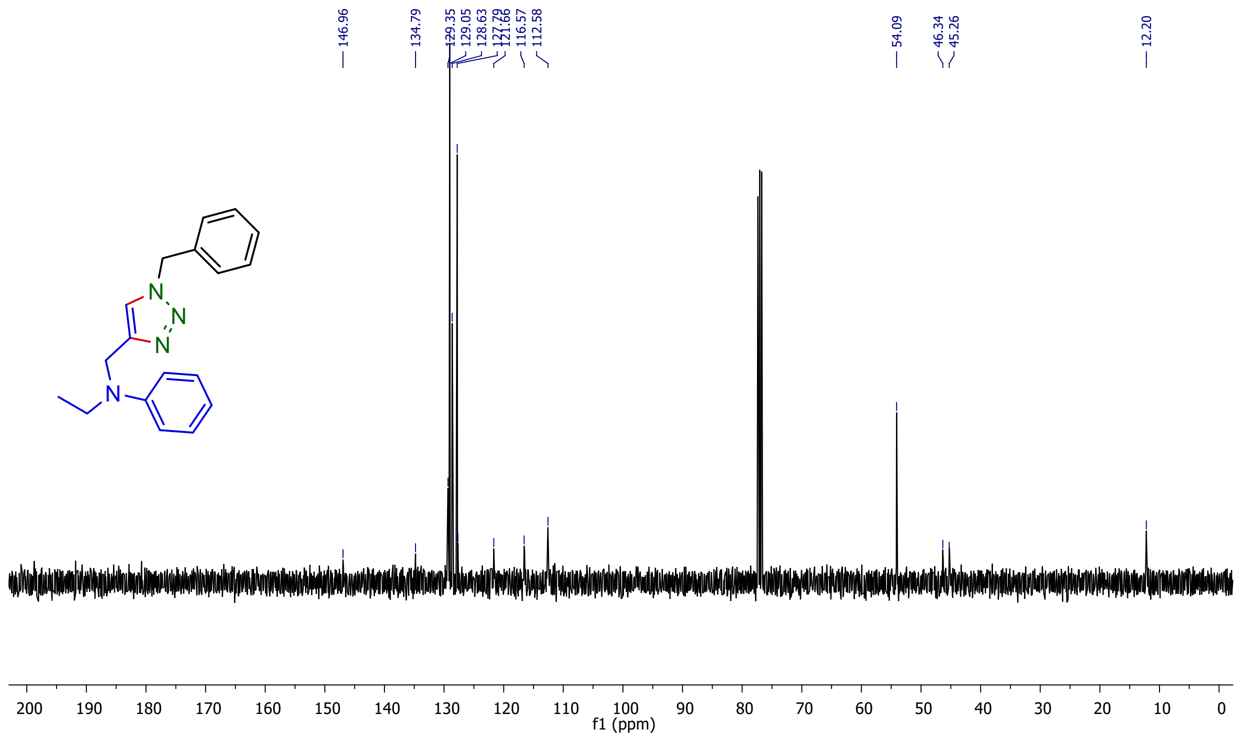

Figure S44. $^{13}C\{^1H\}$ NMR spectrum of (**4m**) in $CDCl_3$ (101 MHz).

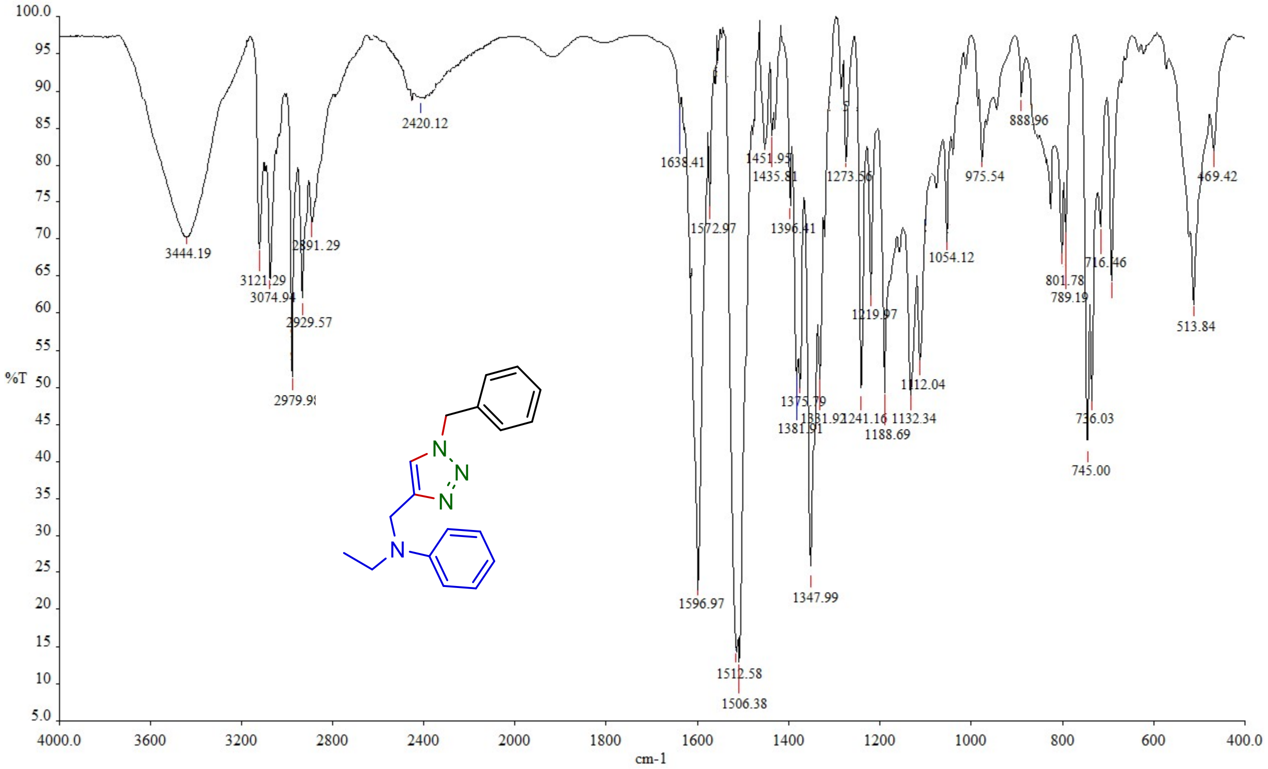

Figure S45. FT-IR spectrum of (**4m**) in KBr

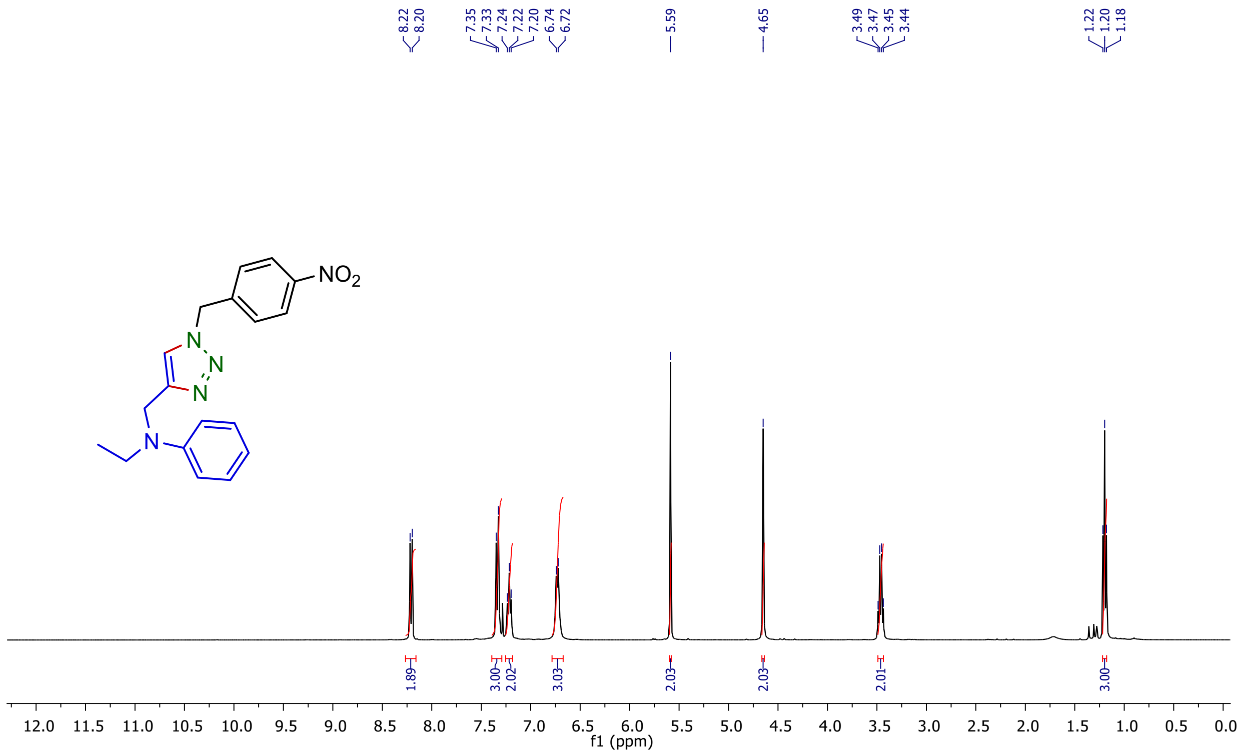

Figure S46. ^{1}H NMR spectrum of (**4n**) in $CDCl_3$ (400 MHz).

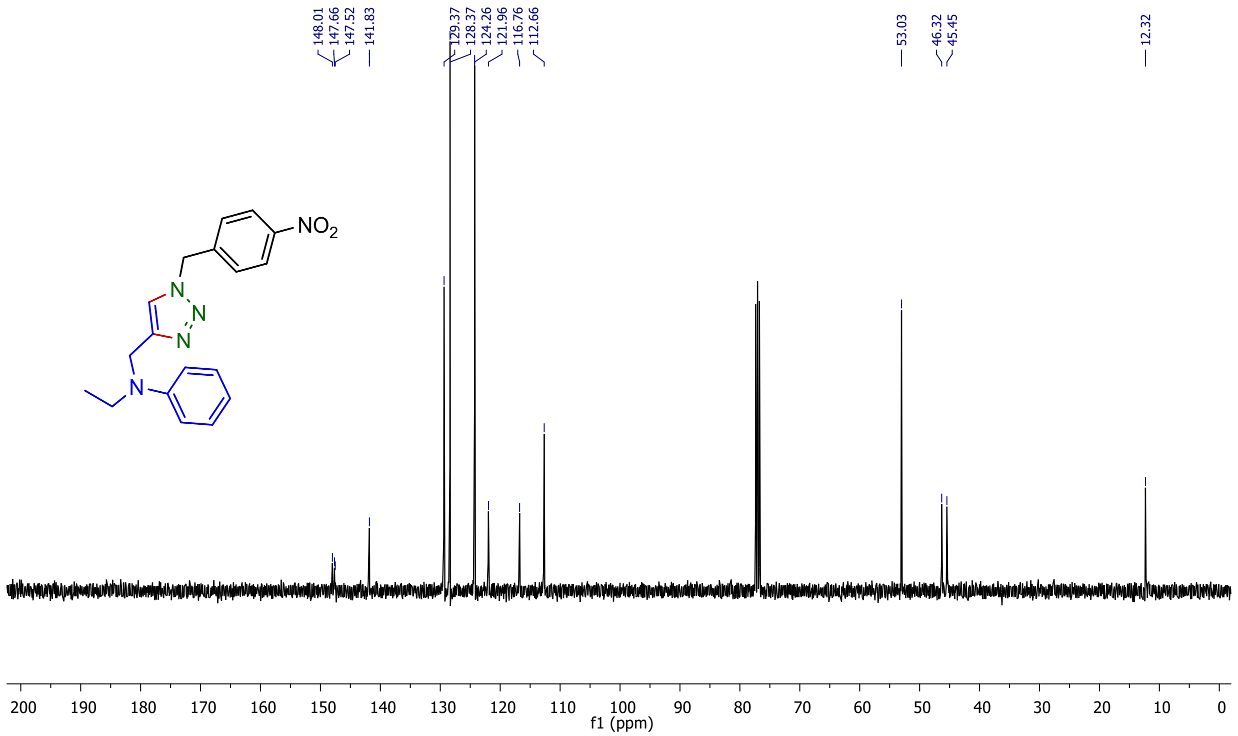

Figure S47. $^{13}C\{^{1}H\}$ NMR spectrum of (**4n**) in $CDCl_3$ (101 MHz).

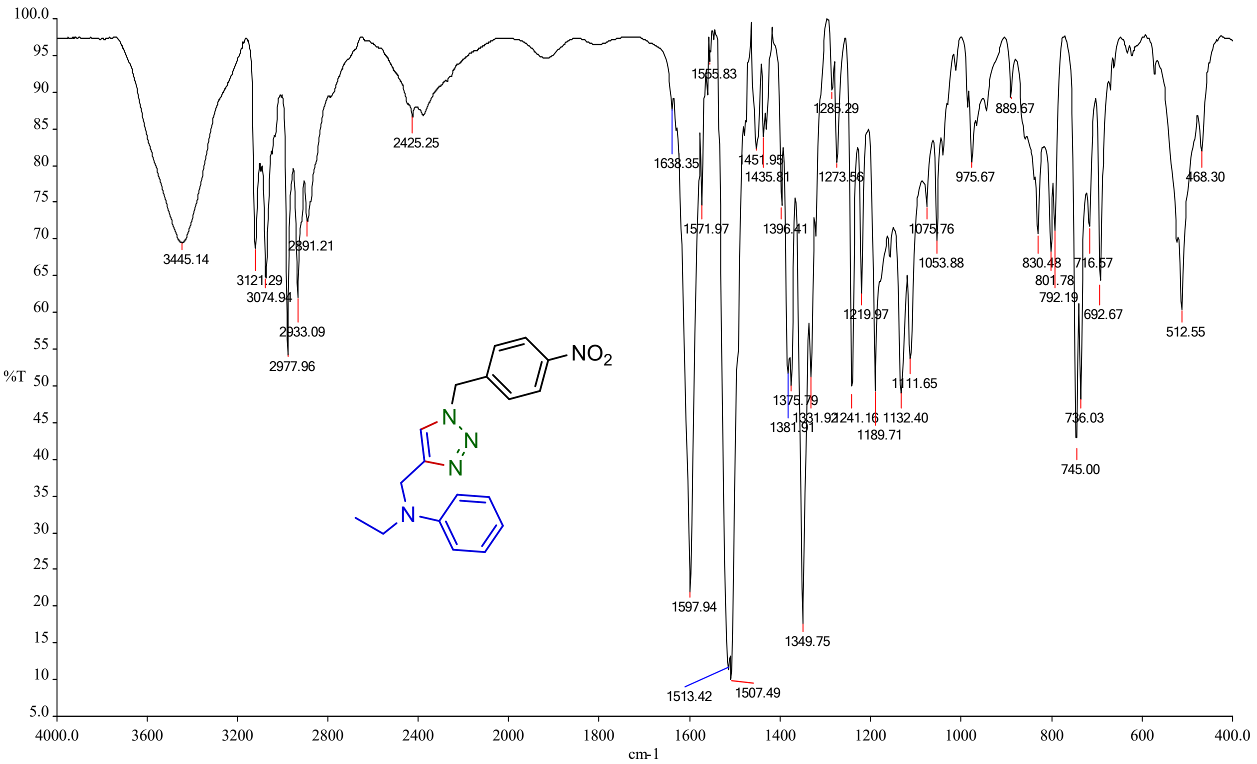

Figure S48. FT-IR spectrum of (**4n**) in KBr

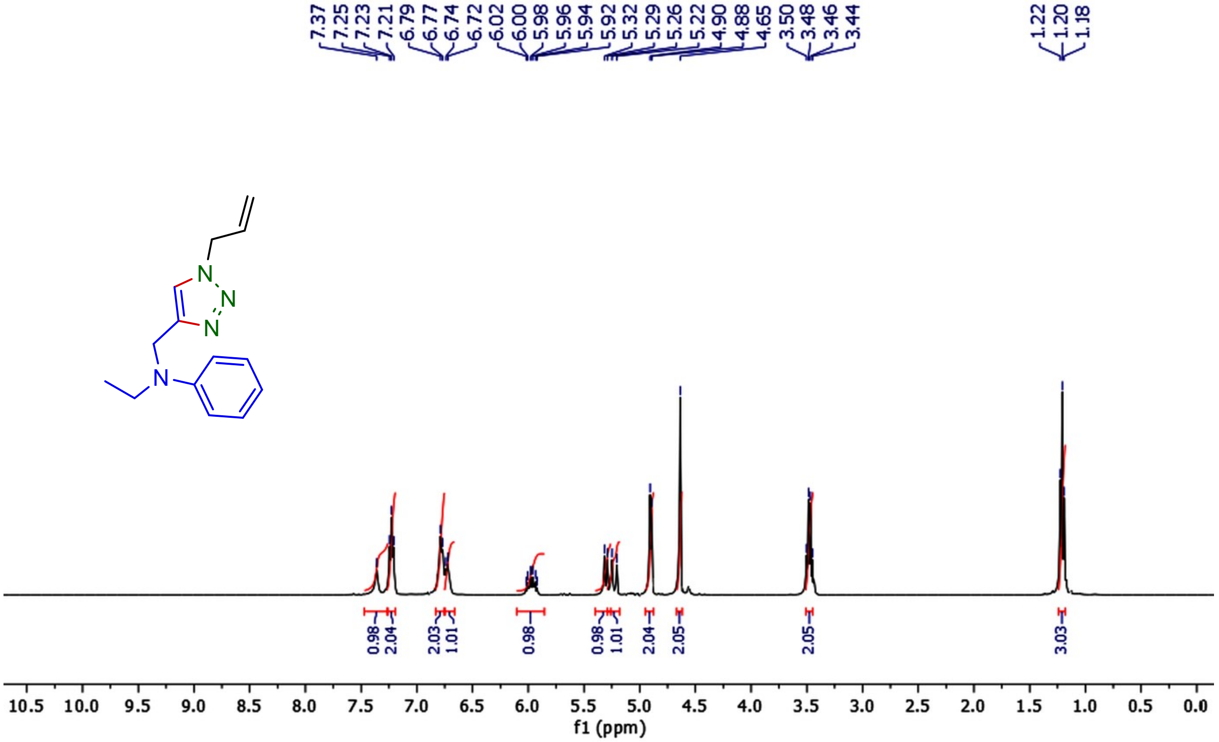

Figure S49. ^{1}H NMR spectrum of (**4o**) in $CDCl_3$ (400 MHz).

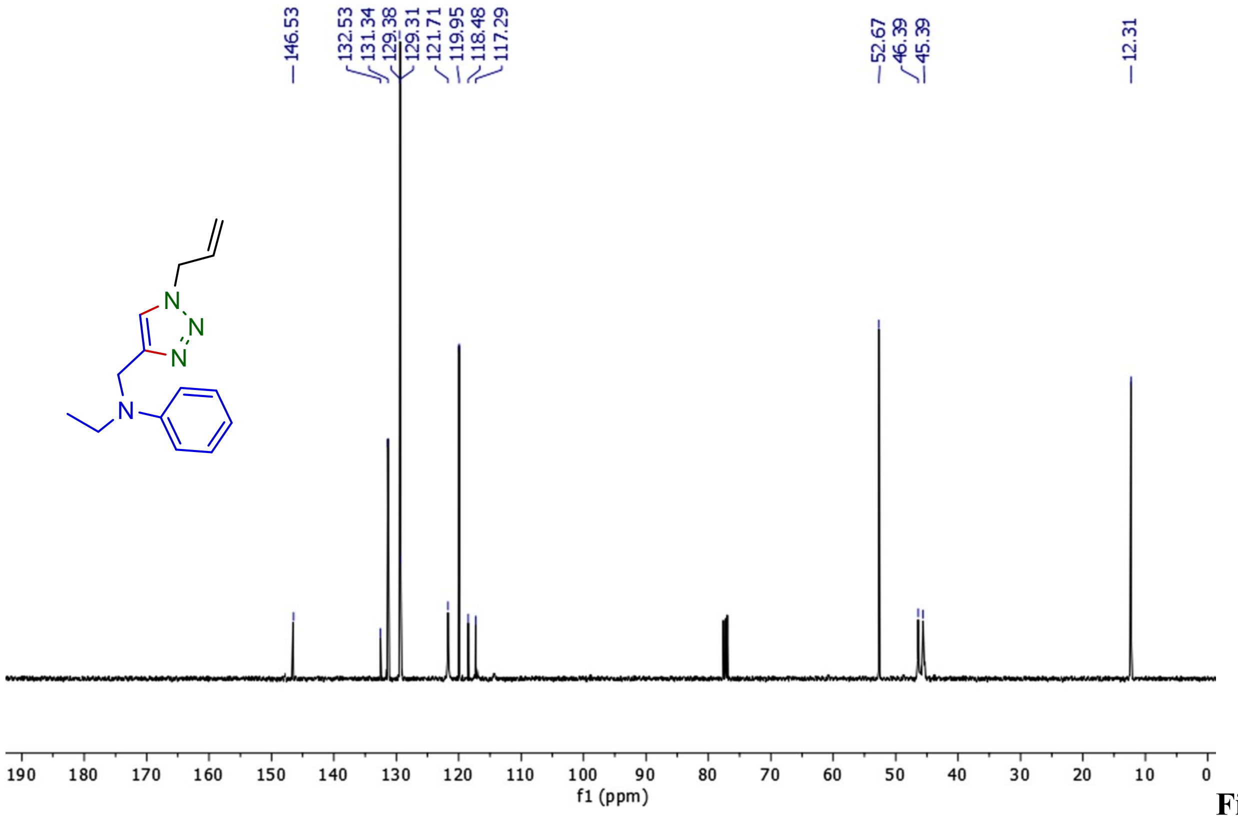

Fi

gure S50. $^{13}C\{^{1}H\}$ NMR spectrum of (**4o**) in $CDCl_3$ (101 MHz).

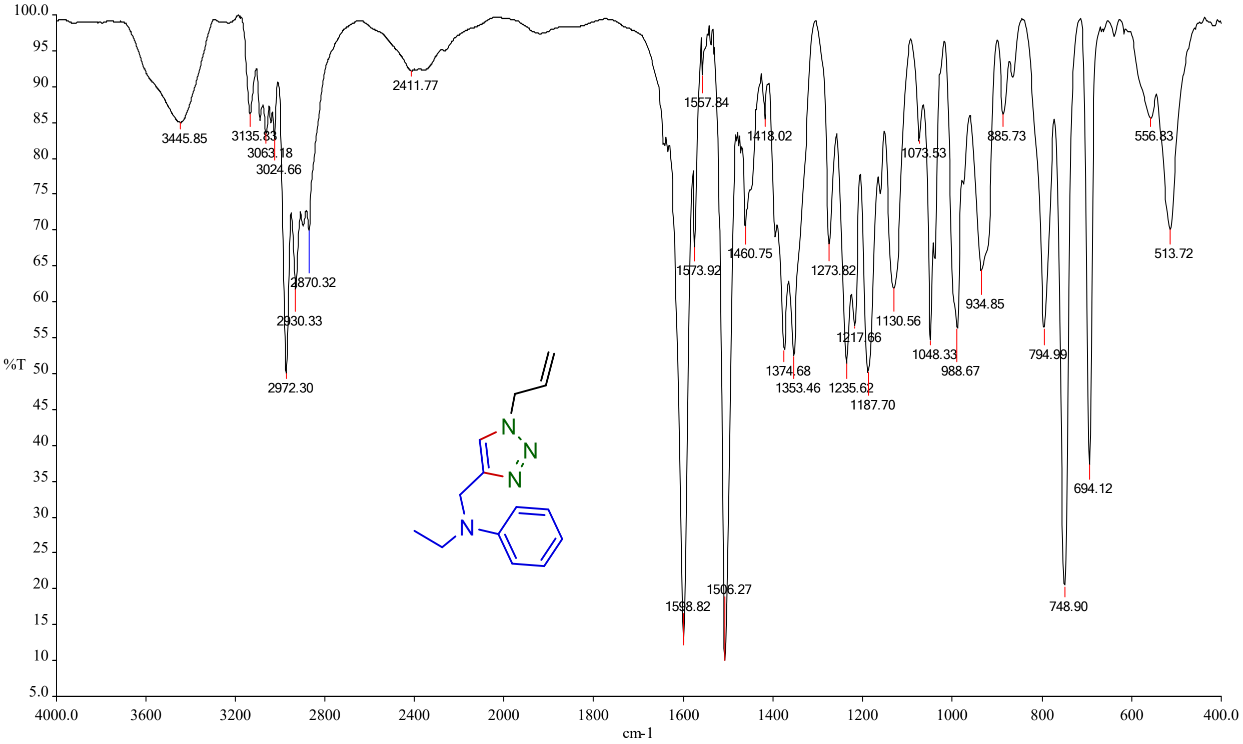

Figure S51. FT-IR spectrum of (**4o**) in KBr

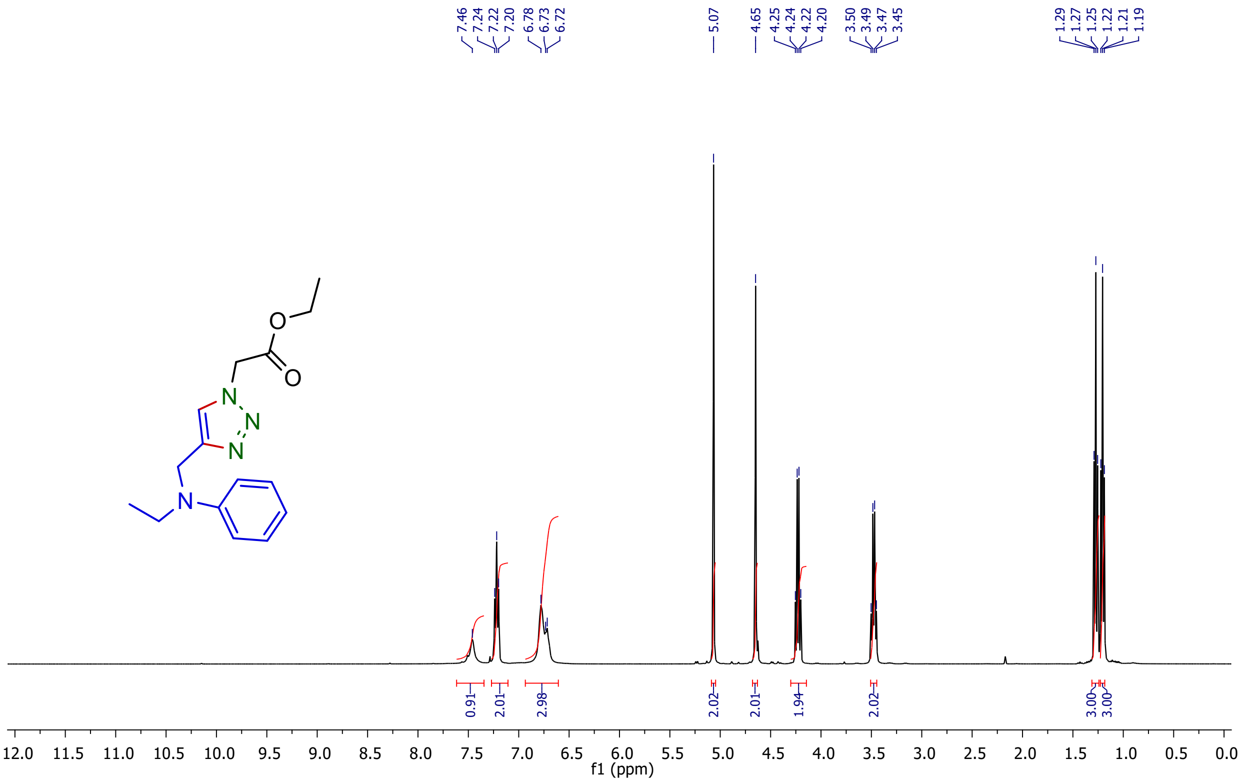

Figure S52. ^{1}H NMR spectrum of (**4p**) in $CDCl_3$ (400 MHz).

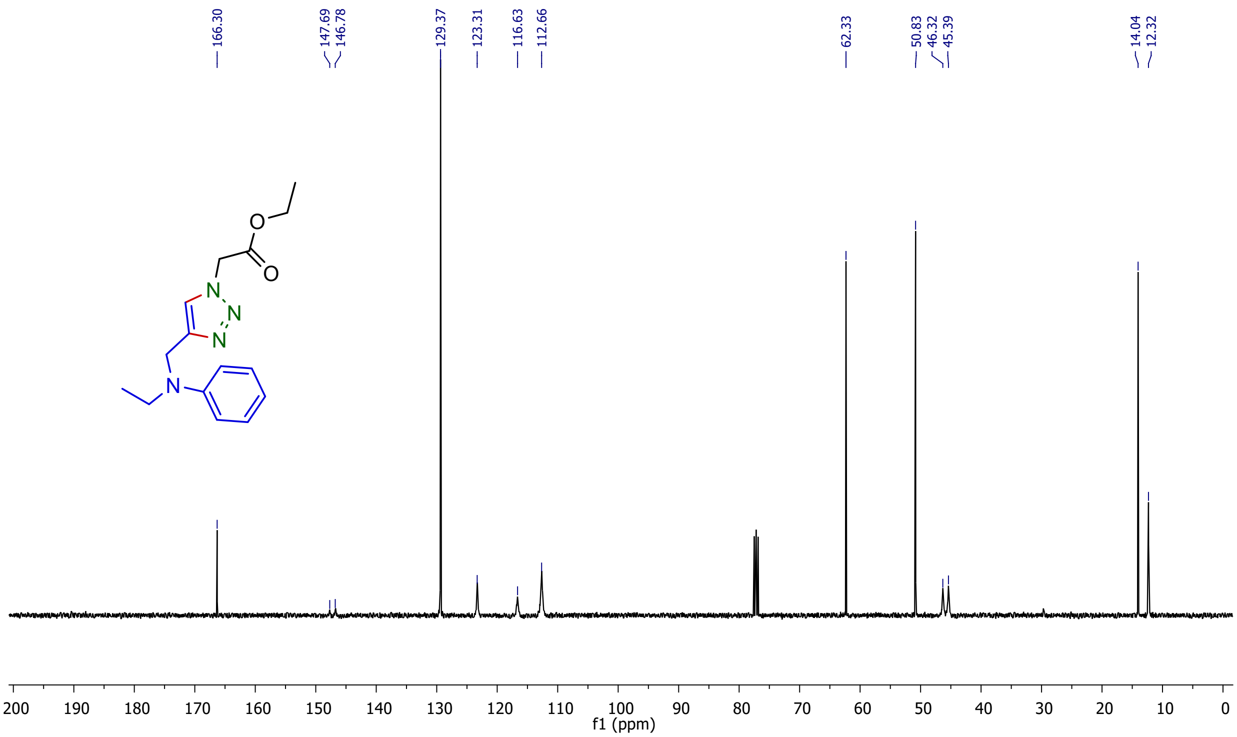

Figure S53. $^{13}C\{^{1}H\}$ NMR spectrum of (**4p**) in $CDCl_3$ (101 MHz).

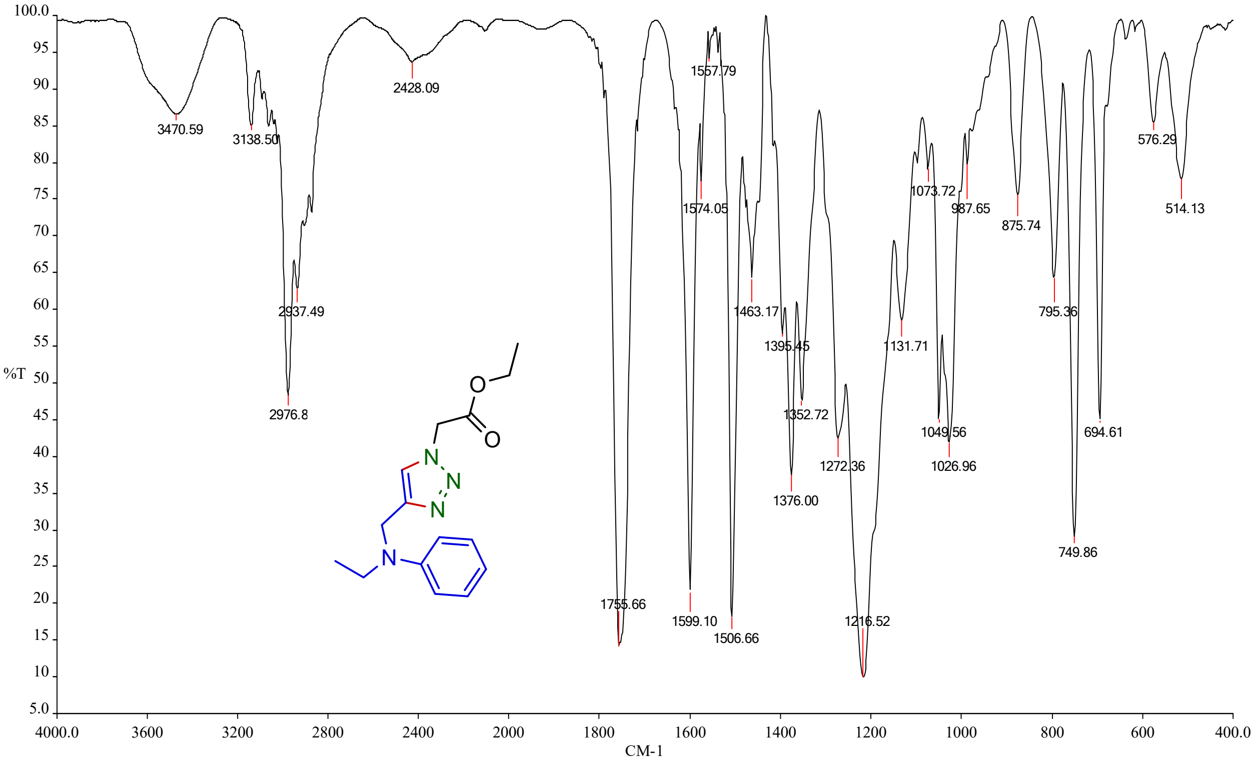

Figure 54. FT-IR spectrum of (**4p**) in KBr

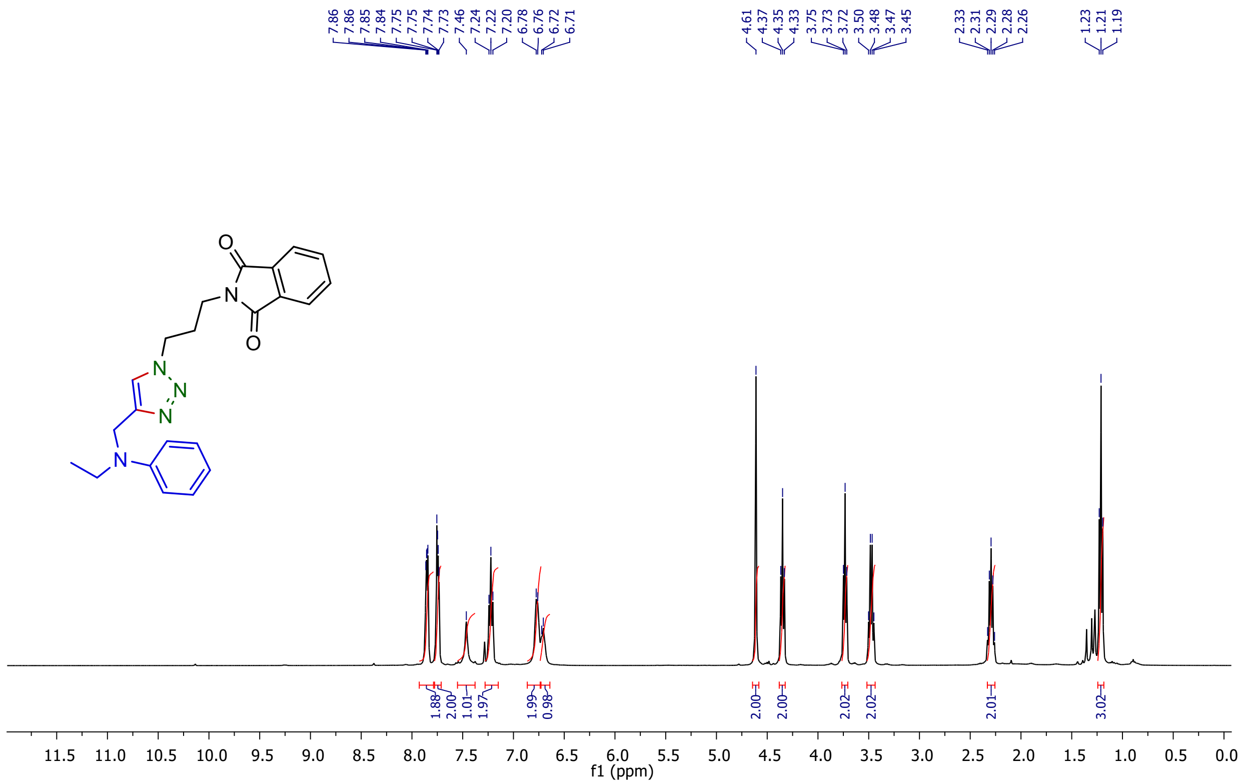

Figure S55. ^{1}H NMR spectrum of (**4q**) in $CDCl_3$ (400 MHz).

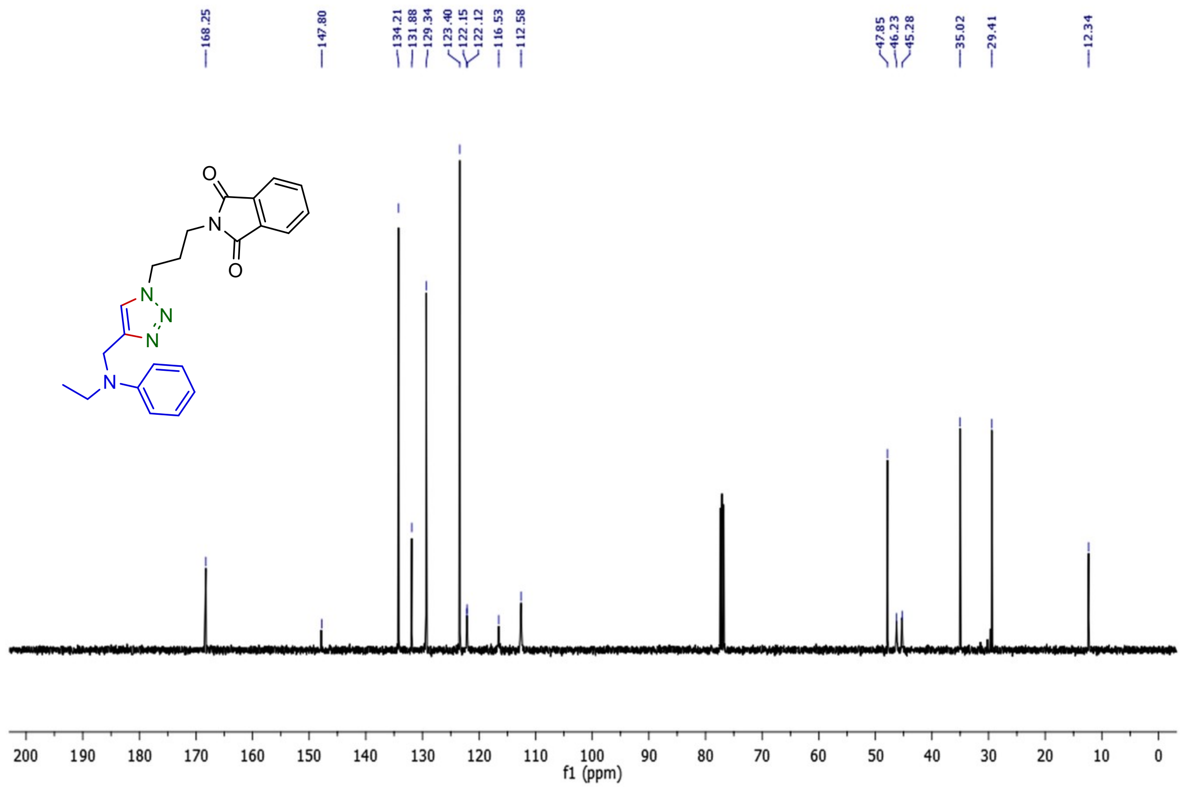

Figure S56. $^{13}C\{^{1}H\}$ NMR spectrum of (**4q**) in $CDCl_3$ (101 MHz).

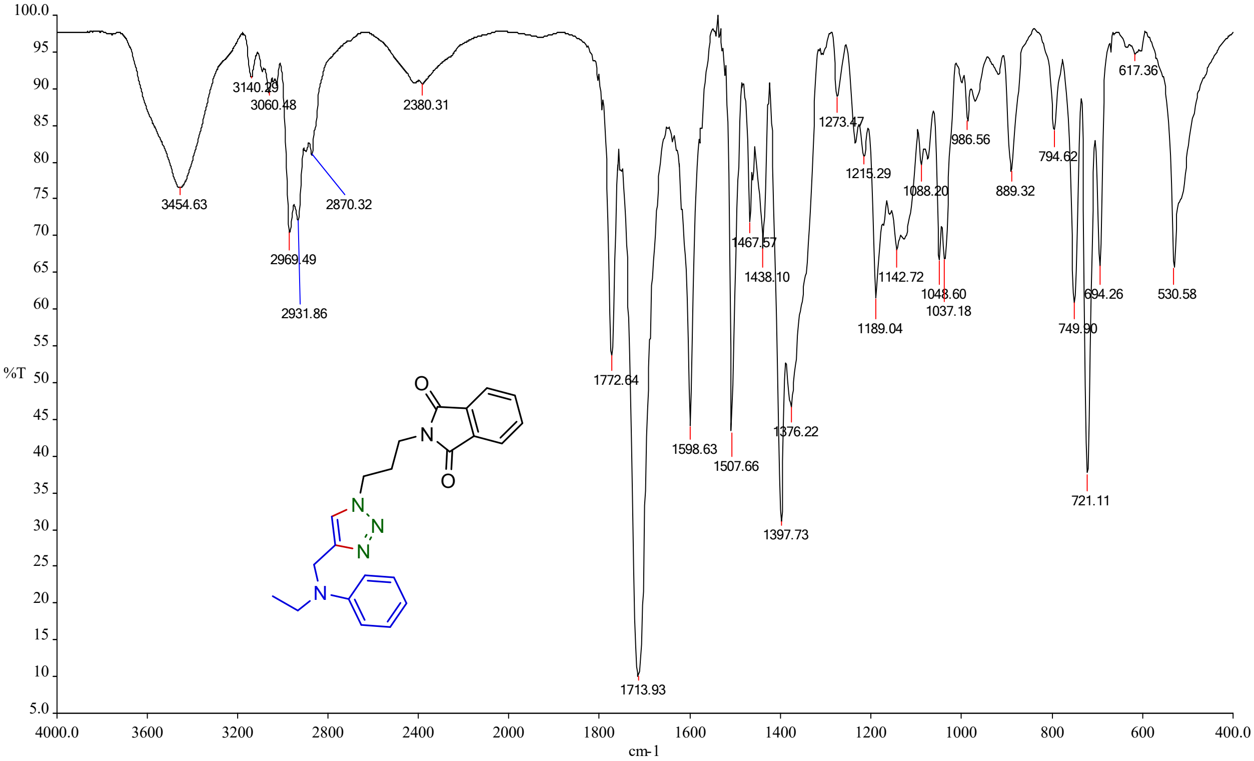

Figure S57. FT-IR spectrum of (**4q**) in KBr

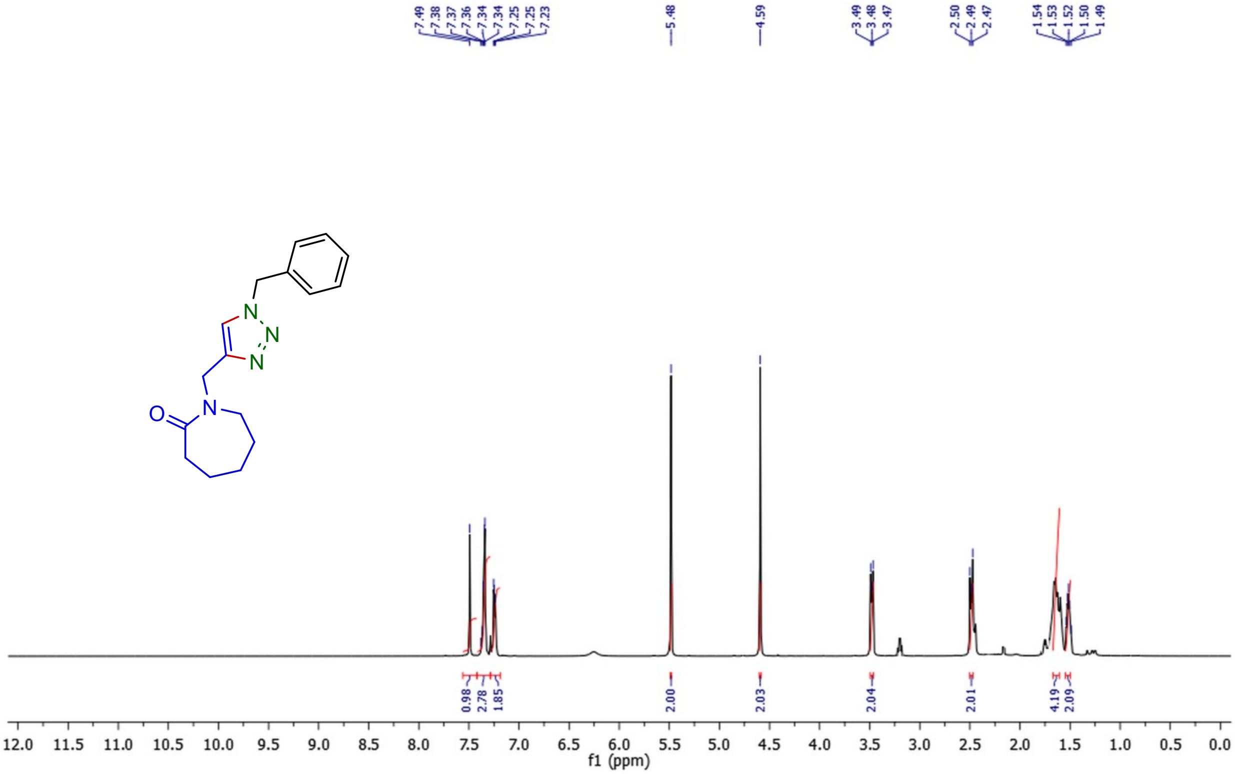

Figure S58. ^{1}H NMR spectrum of (**4r**) in $CDCl_3$ (400 MHz).

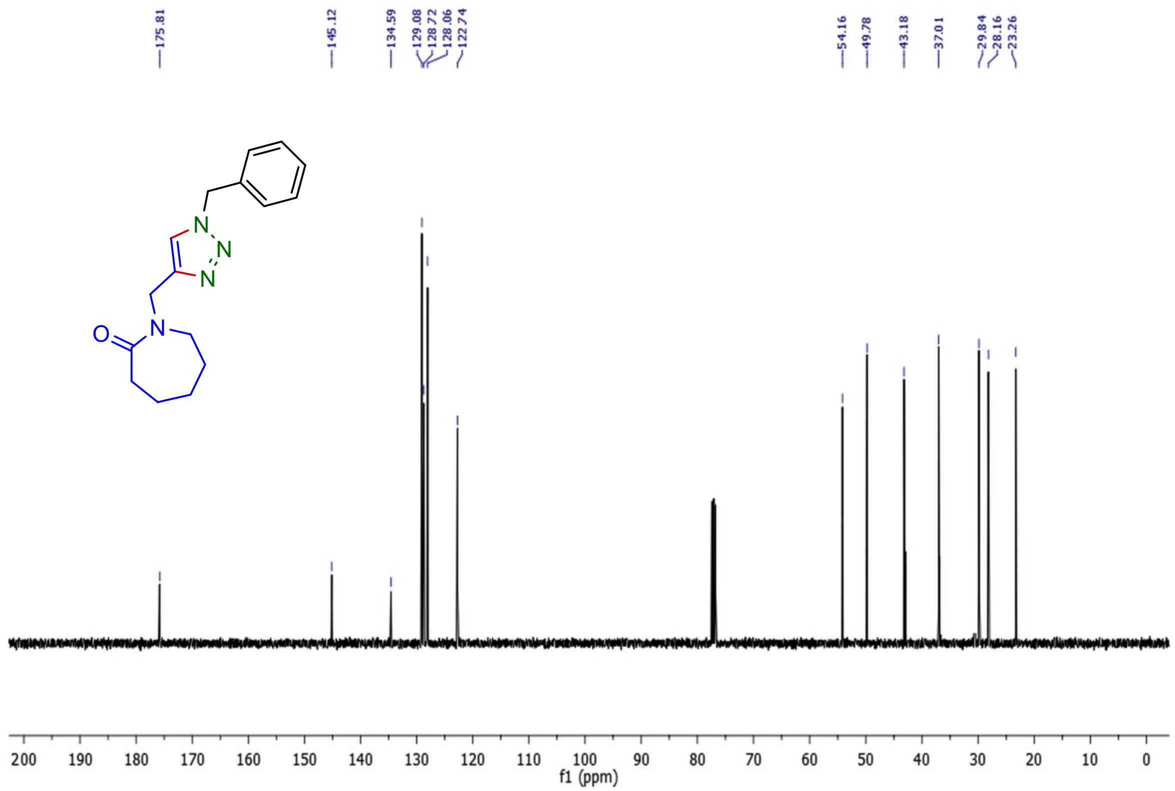

Figure S59. $^{13}C\{^{1}H\}$ NMR spectrum of (**4r**) in $CDCl_3$ (101 MHz).

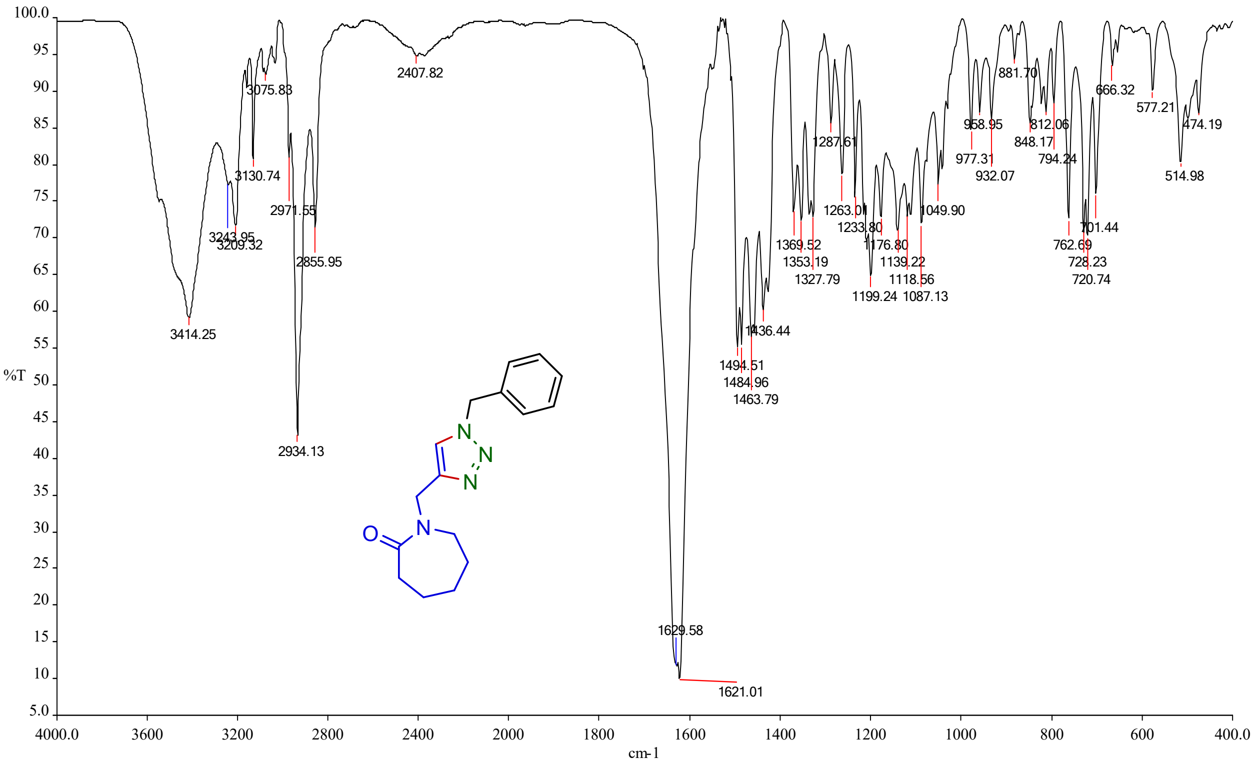

Figure S60. FT-IR spectrum of (**4r**) in KBr

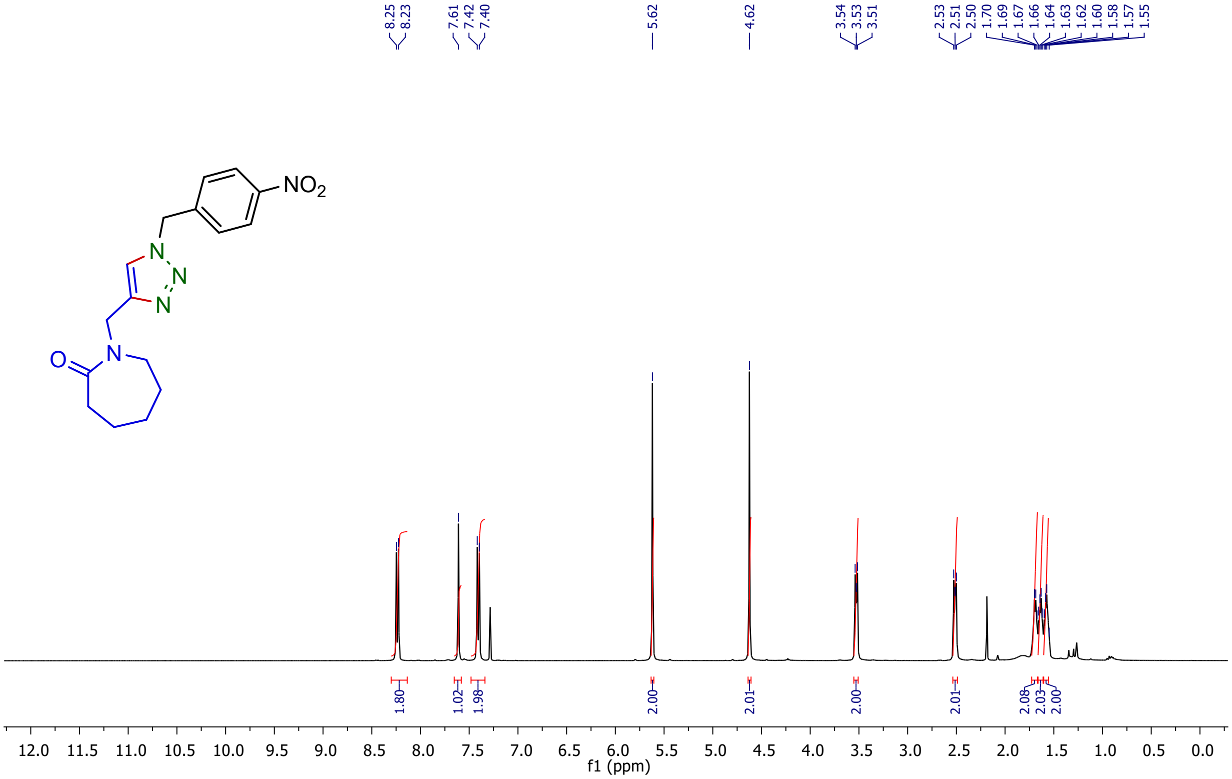

Figure S61. ^{1}H NMR spectrum of (**4s**) in $CDCl_3$ (400 MHz).

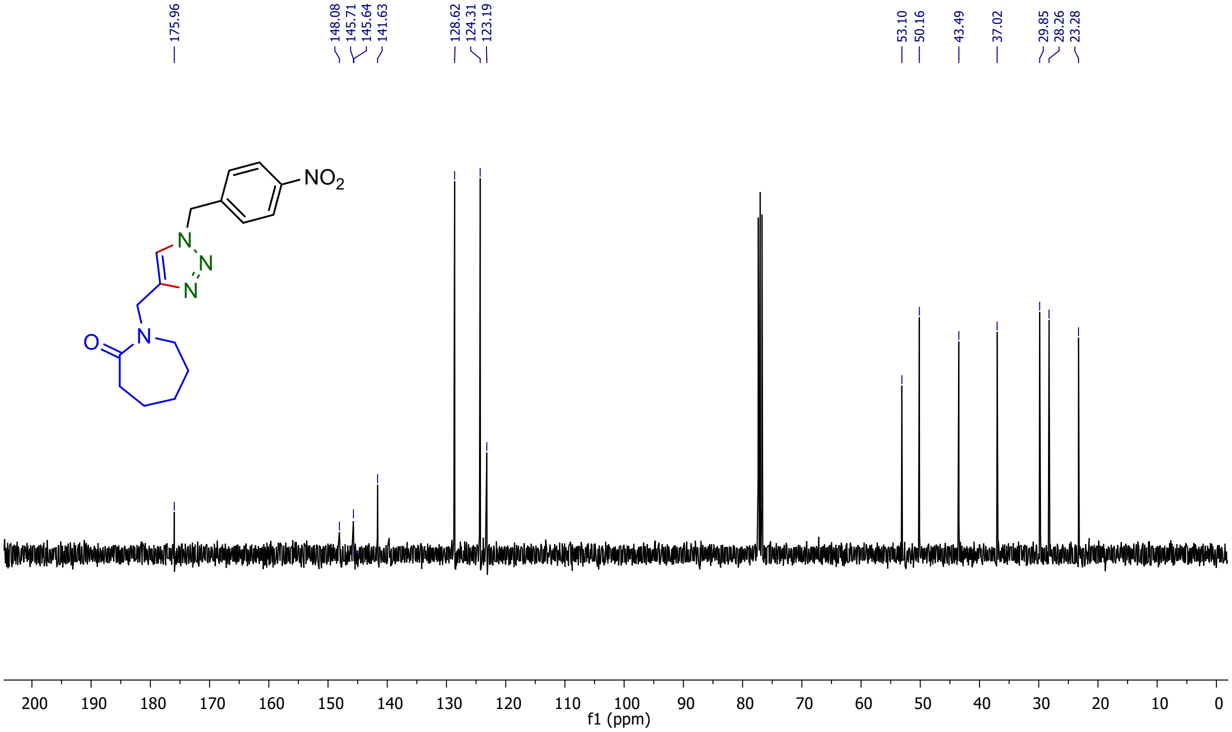

Figure S62. $^{13}C\{^{1}H\}$ NMR spectrum of (**4s**) in $CDCl_3$ (101 MHz).

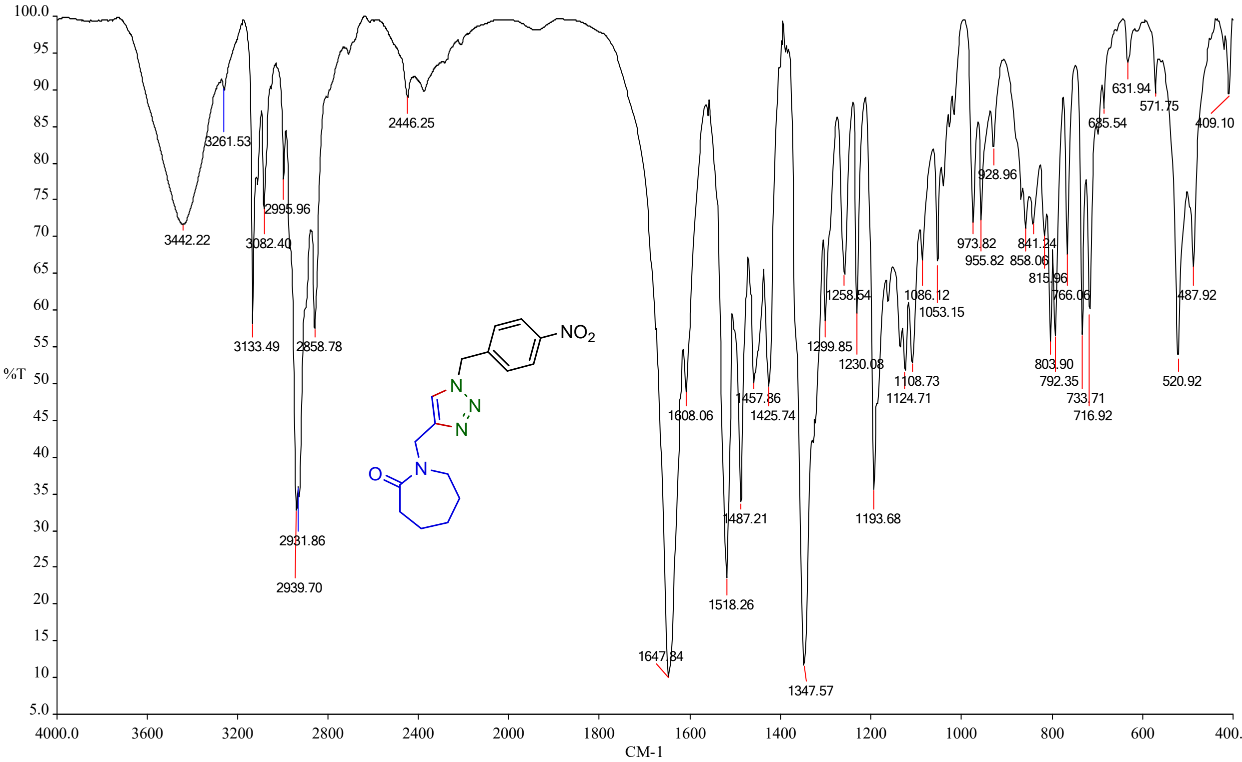

Figure S63. FT-IR spectrum of (**4s**) in KBr

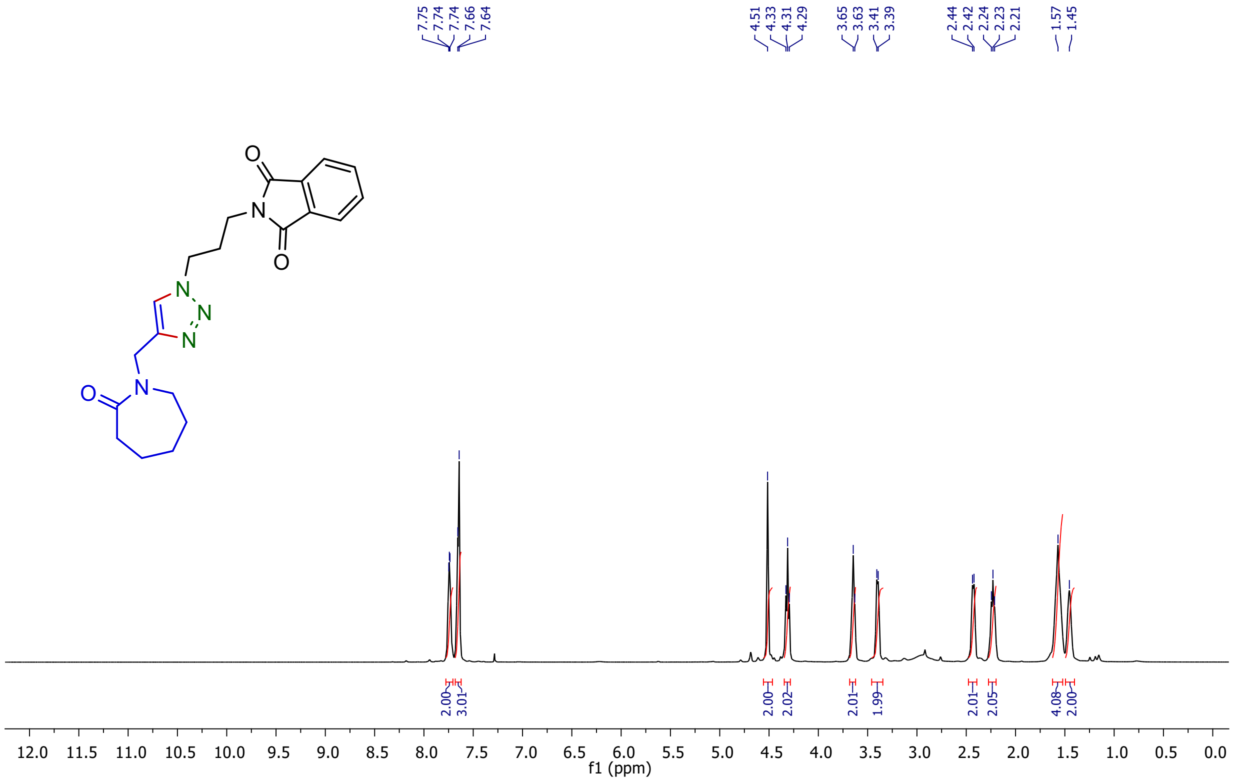

Figure S64. ^{1}H NMR spectrum of (**4t**) in $CDCl_3$ (400 MHz).

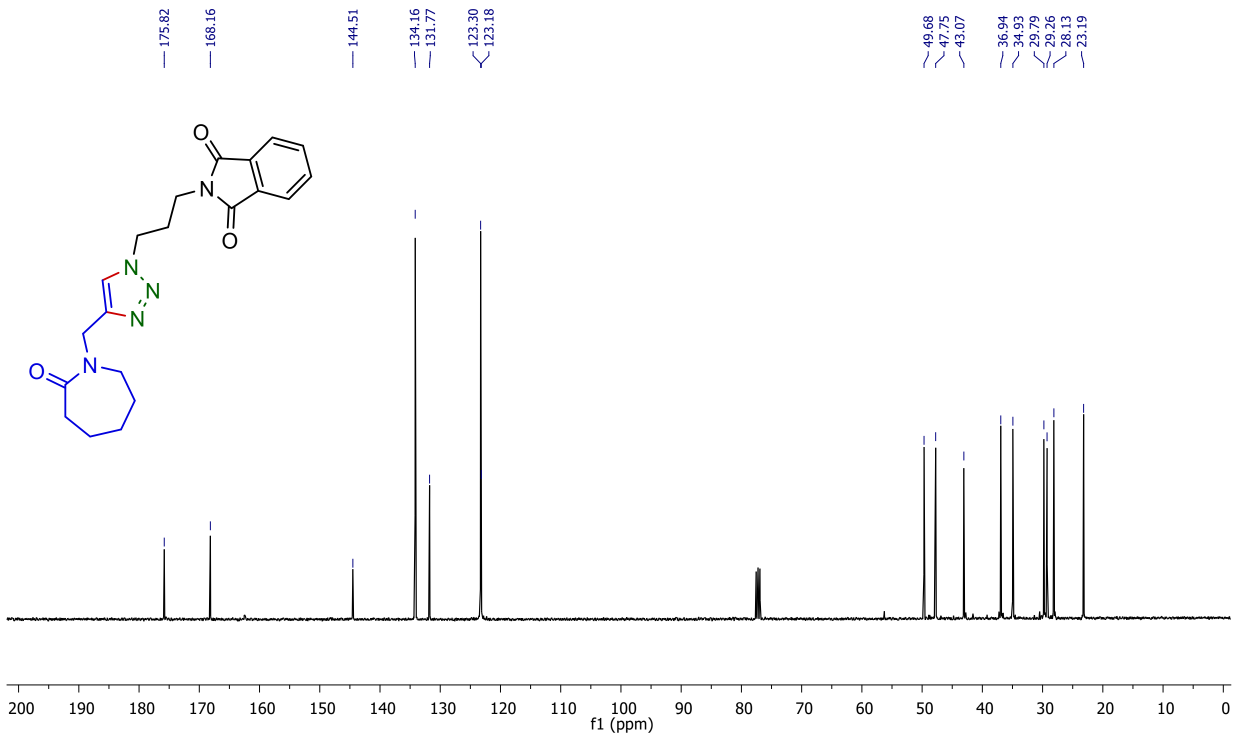

Figure S65. $^{13}C\{^1H\}$ NMR spectrum of (**4t**) in $CDCl_3$ (101 MHz).

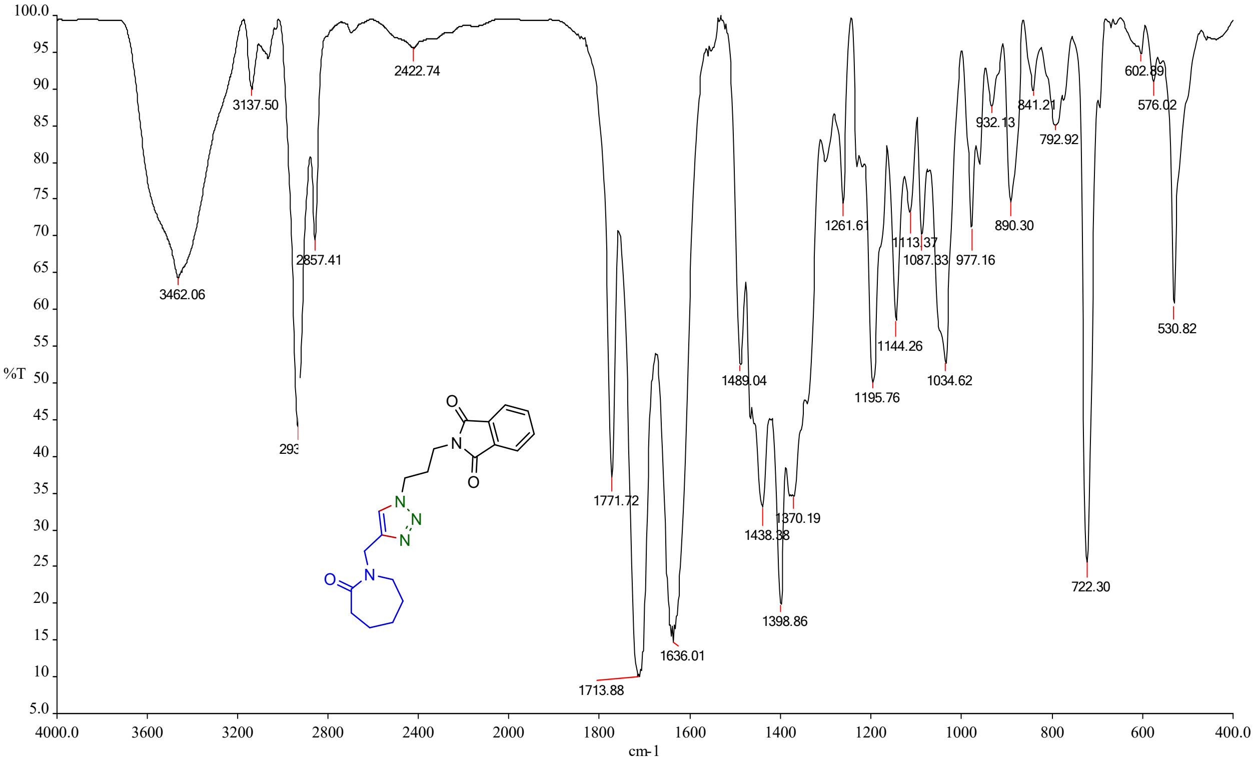

Figure S66. FT-IR spectrum of (**4t**) in KBr

References

[1] S. P. Santoso, V. Bundjaja, A. E. Angkawijaya, C. Gunarto, A. Woo Go, M. Yuliana, P. L. Tran-Nguyen, C.W. Hsieh, Y. H. Ju, *Sci Rep.*, **2021**, *11*, 12021.

[2] (a) N. Bagherzadeh, A. R. Sardarian and I. Dindarloo Inaloo, *New J. Chem.*, **2021**, *45*, 11852-11858; (b) Z. Maugeri, P. D. de María, *RSC Adv.*, **2012**, *2*, 421-425; (c) Q. Zhang, K. D. O. Vigier, S. Royer and F. Jérôme, *Chem.Soc.Rev.,* **2012**, *41*, 7108-7146.

[3] M. Erdoğan and A. Daştan, *Synth. Commun.* **2020**, *50*, 3845-3853.

[4] G. Li, C. Wang, Y. Li, K. Shao, G. Yu, S. Wang, X. Guo, W. Zhao and H. Nakamura, *Chem. Commun.* **2020**, *56*, 7333-7336.

[5] B. Rajagopal, C. H. Chou, C. C. Chung and P. C. Lin, *Org. Lett.* **2014**, *16*, 3752-3755.

[6] G. Mohammadnezhad, A. A. Amirian, H. Görls, W. Plass, A. Sandleben, S. Schäfer and A. Klein, *Eur. J. Inorg. Chem.* **2021**, *2021*, 1140-1151.

[7] S. P. D. O. Assis, M. T. D. Silva, F. T. D. Silva, M. P. Sant'Anna, C. D. A. Tenório, C. F. B. D. Santos, C. S. M. D. Fonseca, G. Seabra, V. L. Lima and R. D. Oliveira, *Chem. Pharm. Bull.* **2019**, *67*, 96-105.

[8] M. A. Topchiy, A. A. Ageshina, P. S. Gribanov, S. M. Masoud, T. R. Akmalov, S. E. Nefedov, S. N. Osipov, M. S. Nechaev and A. F. Asachenko, *Eur. J. Org. Chem.* **2019** *2019*, 1016-1020.
